# Synthesis of Jacaranone-Derived Nitrogenous Cyclohexadienones and Their Antiproliferative and Antiprotozoal Activities

**DOI:** 10.3390/molecules23112902

**Published:** 2018-11-07

**Authors:** Armin Presser, Gunda Lainer, Nadine Kretschmer, Wolfgang Schuehly, Robert Saf, Marcel Kaiser, Marc-Manuel Kalt

**Affiliations:** 1 Institute of Pharmaceutical Sciences, Pharmaceutical Chemistry, University of Graz, Schubertstrasse 1, 8010 Graz, Austria; gunda.lainer@edu.uni-graz.at (G.L.); marc.kalt@edu.uni-graz (M.-M.K.); 2 Institute of Pharmaceutical Sciences, Pharmacognosy, University of Graz, Universitaetsplatz 4, 8010 Graz, Austria; nadine.kretschmer@uni-graz.at (N.K.); wolfgang.schuehly@uni-graz.at (W.S.); 3 Institute for Chemistry and Technology of Materials (ICTM), Graz University of Technology, Stremayrgasse 9, 8010 Graz, Austria; robert.saf@tugraz.at; 4 Swiss Tropical and Public Health Institute, Socinstrasse 57, 4002 Basel, Switzerland; marcel.kaiser@swisstph.ch; 5 University of Basel, Petersplatz 1, 4003 Basel, Switzerland

**Keywords:** antiproliferative activity, antiprotozoal activity, green chemistry, natural products, jacaranone

## Abstract

The cytotoxic and antiprotozoal activities of the phytoquinoide, jacaranone, and related compounds have been an ongoing topic in recent drug discovery. Starting from the natural product-derived cyclohexadienone scaffold, a series of nitrogen-containing derivatives were synthesized and subsequently evaluated for their antiproliferative and antiprotozoal activity. Anticancer potency was analyzed using different types of cancer cell lines: MDA-MB-231 breast cancer, CCRF-CEM leukemia, HCT-116 colon cancer, U251 glioblastoma, and, in addition, non-tumorigenic MRC-5 lung fibroblasts. Antiproliferative activities at micromolar concentrations could be shown. Antiprotozoal activity was assessed against *Plasmodium falciparum* NF54 and *Trypanosoma brucei rhodesiense* STIB900. For all compounds, selectivity indices (SI) were calculated based on assessed cytotoxicity towards L6 cells. In addition, the structure-activity-relationships and physicochemical parameters of these compounds are discussed.

## 1. Introduction

Natural products (NPs) play vital roles in drug discovery. More than half of the drugs that have been approved over the past 30 years are natural compounds or compounds based on these [1]. Approximately 68% of anti-infectives are classified as nature-derived or inspired, and 80% of all anticancer compounds fall into this category [2]. Examples of well-known drugs derived from natural products are paclitaxel or doxorubicin (anticancer), artemisinin (antimalarial), daptomycin (antibacterial), and morphine (analgesic). The most striking feature of many natural products is their structural diversity, which is still largely untapped. About 40% of the chemical scaffolds found in NPs are still absent in today’s medicinal chemistry [3]. The importance of NPs in drug development has been described in a number of reviews and reports [2,4,5,6,7,8,9,10].

Jacaranone (**1**) and its derivatives, phytoquinoids isolated from several *Jacaranda* and *Senecio* species, exhibited promising pharmacophore qualities in previous investigations. The remarkable cytotoxic and antiprotozoal properties of jacaranone have been especially well-studied both in vitro and in vivo [11,12,13,14]. Related nitrogenous NPs (e.g., verongiaquinol (**2**) or melodamide A (**3**)) are also valuable drug candidates that possess antiproliferative, antibiotic, antiviral, antiprotozoal, and anti-inflammatory activities [15,16,17,18,19,20].

**Figure 1:**
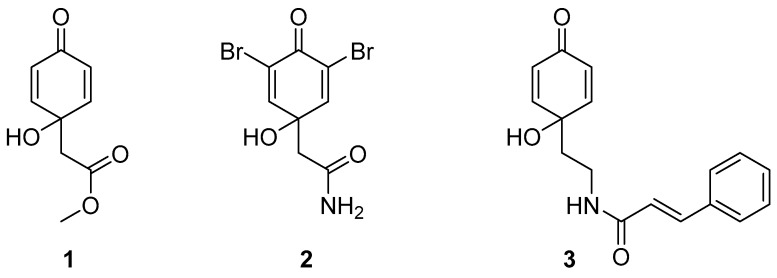
Naturally occurring quinols with remarkable biological activity.

Herein, we report the design and synthesis of jacaranone-inspired *N*-containing cyclohexadienones and our findings on the antiproliferative, antiplasmodial, and antitrypanosomal activities of these NP-derived quinols (Figure 1).

## 2. Results and Discussion

### 2.1. Chemistry

Imide derivatives are a valuable group of bioactive compounds. In spite of their wide applicability, available procedures for their synthesis are limited [21]. During the course of our work on jacaranone imides, we searched for an efficient method that we could use to construct the *N*-dienone scaffold.

**Scheme 1:**
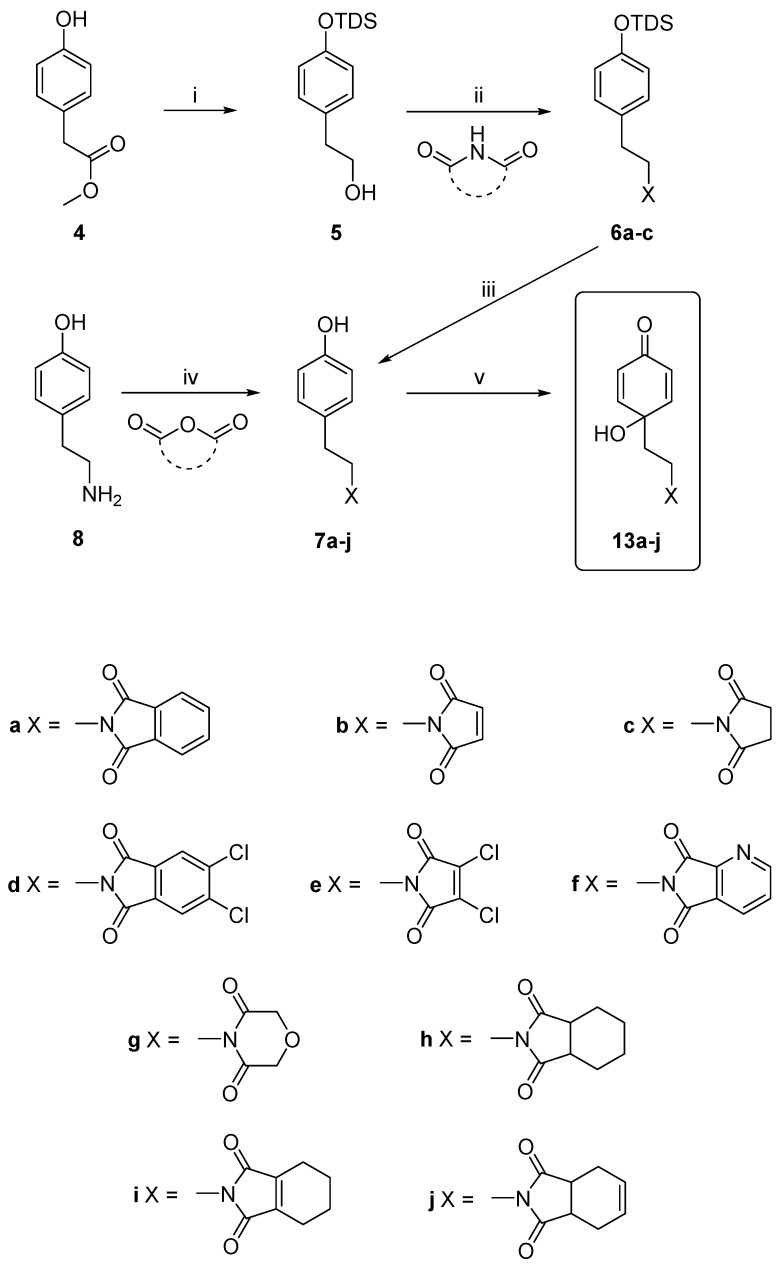
Reagents and conditions: method A: (i) 1. TDSCl, DBU, RT, 1.5 h; 2. LiAlH_4_, THF, 0 °C, 2 h (98%); (ii) imide, PPh_3_, DIAD, THF, 0 °C→RT, 2.5–24 h (**6a**: 90%, **6b**: 70%, **6c**: 77%); (iii) TBAF, CH_2_Cl_2_, RT, 1 h (**7a**: 80%, **7b**: 85%, **7c**: 82%); (iv) anhydride, AcOH, 120 °C, 1.5 h (method B: **7a**: 73%, **7b**: 55%, **7c**: 58%, **7d**: 89%, **7e**: 90%, **7f**: 0%, **7g**: 0%, **7h**: 86%, **7i**: 92%, **7j**: 79%) or PEG 400, 140 °C, 4 h (method C: **7a**: 23%, **7b**: 51%, **7c**: 98%, **7d**: 67%, **7e**: 67%, **7f**: 86%, **7g**: 71%, **7h**: 79%, **7i**: 98%, **7j**: 98%); (v) PhI(OAc)_2_, CH_3_CN/H_2_O (12:5), 0 °C, 7 min (**13a**: 67%, **13b**: 17%, **13c**: 55%, **13d**: 40%, **13e**: 64%, **13f**: 19%, **13g**: 18%, **13h**: 79%, **13i**: 88%, **13j**: 49%.

During our initial attempts, we followed a Mitsunobu route [22] as summarized in Scheme 1 (method A). Starting from the commercially available compound, methyl 4-hydroxyphenyl acetate (**4**), the temporarily protected alcohol **5** was obtained in two steps in almost quantitative yields. The introduced thexyldimethylsilyl (TDS) group is superior in comparison with other commonly used silyl protecting strategies because of its greater stability and ease of handling [23].

Subsequently, the intermediates **6a**–**c** were prepared from TDS ether **5** under Mitsunobu conditions and then treated with an excess amount of tetrabutylammonium fluoride (TBAF) [24] to afford the unprotected imides **7a**–**c** in moderate overall yields (58–71%).

Walker [25] reported that yields of the crucial Mitsunobu reaction could be increased by simply altering the order in which the reagents were combined. Although we observed these instructions, following the Mitsunobu route still did not allow us to produce a wide range of imide derivatives.

Therefore, we focused on tyramine (**8**) as a commercially available and more suitable starting material for our imide design. A previously described method [26] used **8** for the chemoselective condensation with phthalic anhydride in refluxing acetic acid. Following this published protocol as shown in Scheme 1, a series of cyclic imides (**7a**–**j**) were synthesized in generally good to excellent yields (method B). Furthermore, tyramine has an advantage in that it is significantly more reactive than the formerly used alcohol **5**, and the tedious process of protecting the phenolic group is not necessary. Interestingly, the preparation of **7f** and **7g** failed using this procedure, perhaps due to its basicity (**7f**) or hydrolytic degradation (**7g**).

Recently, we have become interested in the development of synthetic methods using polyethylene glycol (PEG) as a novel, environmentally and industrially friendly medium and promoter [27]. It is known that PEG can act as an excellent reaction medium for the synthesis of *N*-alkyl and *N*-arylphthalimides [28]. For this reason, we investigated whether the substitution of the established solvent acetic acid (method B) by the nontoxic, inexpensive, nonionic liquid PEG 400 (method C) was a viable alternative procedure for the condensation of various anhydrides with **8**.

Initially, the modified procedure provided only poor yields because PEG acted as a solubilizer. This led to difficulties during the workup of the highly water-soluble imides. By referring to a PEG-assisted solvent and catalyst-free synthesis of 3,4-dihydropyrimidinones [29], we significantly decreased the amount of PEG used in our synthesis. In fact, the results obtained by modifying the method demonstrated that we could considerably increase the product yield in most cases, including the cases of the elusive imides, **7f** and **7g**.

Overall, upon comparing the yields achieved with methods A and B/C, respectively, we could clearly demonstrate the great advantage of using tyramine (**8**) as a starting material to prepare the cyclic imides, **7a**–**j**, as well as the potential of using PEG as an excellent reagent to promote organic reactions.

**Scheme 2:**
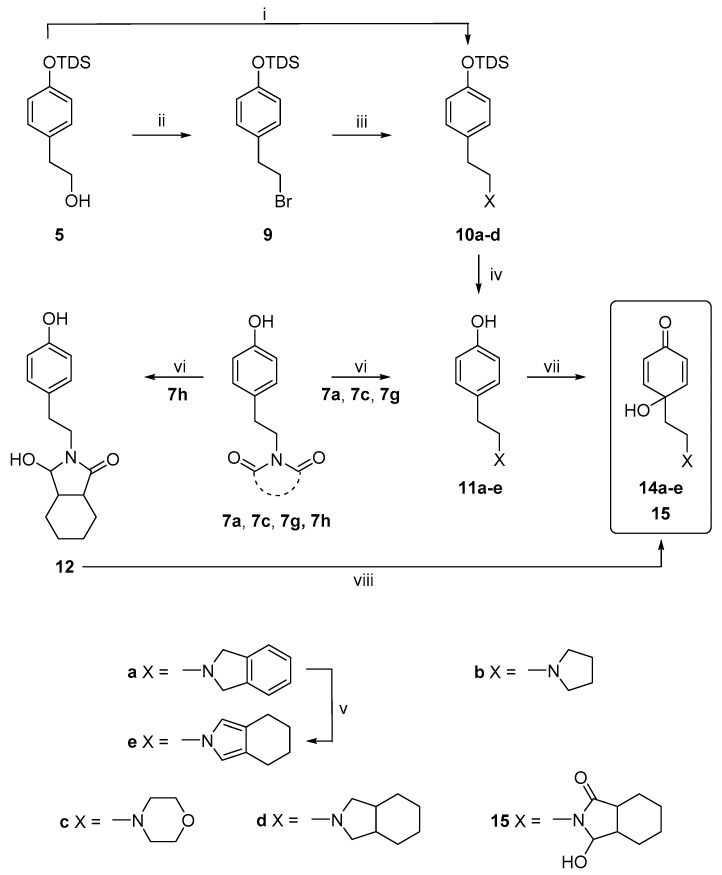
Reagents and conditions: (i) Pyrrolidine, [Cp*IrCl_2_]_2_ (5 mol% Ir), NaHCO_3_, toluene, 110 °C, 25 h (method E: **10b**: 45%); (ii) PPh_3_, DDQ, TBAB, CH_2_Cl_2_, RT, 50 min (81%); (iii) secondary amine, NaI, proton sponge^®^, CH_3_CN, 20 h (method F: **10a**: 72%, **10b**: 87%, **10c**: 87%, **10d**: 94%); (iv) LiOH, DMF, RT, 3–17 h (**11a**: 84%, **11b**: 93%, **11c**: 100%, **11d**: 82%); (v) ammonium formate, Pd(OH)_2_/C, MeOH, reflux, 16 h (100%); (vi) LiAlH_4_, THF, 0 °C, 90 min (method D: **11a**: 72%, **11b**: 0%, **11c**: 0%, **11d**: 0%, **12**: 88%); (vii) PhI(OAc)_2_, CH_3_CN/H_2_O/phosphate buffer (12:3:2), pH = 6.4, 0 °C, 7 min (**14a**: 16%, **14b**: 0%, **14c**: 28%, **14d**: 0%, **14e**: 0%); (viii) PhI(OAc)_2_, CH_3_CN/H_2_O (12:5), 0 °C, 7 min (**15**: 65%).

Next, we turned our attention to the preparation of the jacaranone-derived amines. *N*-alkyl amines can be obtained by the catalytic amination of alcohols [30] or by the reaction of the respective amine with alkyl halides and an auxiliary base [31]. Heterocyclic *N*-imides have also been smoothly converted into the corresponding amines with LiAlH_4_ in excellent yields [32,33]. This route was initially adopted for the synthesis of amines **11a**–**d** from the previous synthesized imides (Scheme 2, method D).

Watson et al. [32] showed that the reduction of the phthalimide functionality might be problematic due to the comparatively facile oxidation of the emerging isoindoline to the respective isoindole. Surprisingly, the conversion of phthalimide **7a** with LiAlH_4_ gave essentially the isoindoline, **11a,** in good yields, whereas the reduction to the amines, **11b**–**d**, failed. In contrast, the partially reduced derivative, **12**, was exclusively obtained when **7h** was treated with LiAlH_4_.

As an alternative route to the desired jacaranone-derived amines, we examined the frequently used iridium-catalyzed alkylation of alcohols [34] (method E). However, treatment of the primary alcohol, **5**, with pyrrolidine in the presence of [Cp*IrCl_2_]_2_ [35] delivered **10b** in only moderate yields; thus, this synthetic route was abandoned.

Finally, we applied the traditional amination of alkyl halogenides for the preparation of jacaranone amines (method F). For this purpose, alcohol **5** was first converted with tetrabutyl ammonium bromide (TBAB), PPh_3_, and 2,3-dichloro-5,6-dicyanobenzoquinone (DDQ) [36] to the corresponding bromide **9**. This intermediate was then coupled with the appropriate secondary amine in the presence of NaI and proton sponge^®^ [37] to obtain the amines, **10b**–**d**, in good to excellent yields. Only the *N*-alkylation of isoindoline (**10a**) led to slightly lower yields under these conditions. Deprotection of the obtained amines, **10a**–**d**, with LiOH in DMF [38] was successful, resulting in the desired derivatives, **11a**–**d**.

Previous studies have demonstrated that isoindolines can be readily converted to tetrahydroisoindoles by palladium hydroxide-catalyzed hydrogenation [39,40]. To broaden the range of usable amines, we examined the reported procedure and observed a quantitative conversion of **11a** to the expected tetrahydroisoindole, **11e**.

Finally, we investigated the oxidative dearomatization of the synthesized *N*-containing phenols into the respective p-alkyl quinols. Such cyclic dienones exhibit not only promising pharmacophores [3,41], but are also attractive intermediates for enantioselective natural product synthesis [42,43,44]. Phenol dearomatization processes are generally mediated by hypervalent iodine reagents, and are well-documented in the literature [43,45]. The commonly used protocol using phenyliodine(III) diacetate (PIDA) in aqueous CH_3_CN [46] appeared to be most suitable for our purpose.

The conversion of the compounds, **7a**–**j** and **12,** with PIDA afforded the respective p-substituted cyclohexadienones, **13a**–**j** and **15**, in satisfactory yields, whereas the oxidation of all tertiary amines (**11a**–**e**) failed. To improve the rate of conversion for these compounds, we examined the impact of varying the pH values on the reaction outcome. The best results were obtained applying a 1 M phosphate buffer that adjusted the pH to 6.4 during the oxidation. This variation led to the availability of a few additional tertiary jacaranone amines (**14a**,**14c**), but the yields still remained far from being satisfactory.

Subsequently, the synthesized dienones were evaluated for their antiproliferative and antiprotozoal activity, following the method described in the Experimental section.

### 2.2. Physicochemical Properties

Physicochemical parameters play crucial roles in the selection process of drug candidates for product development. The properties of small molecules, especially those that are orally bioavailable, are concentrated in a relatively narrow range of physicochemical space known as the “drug-like space” [47]. An optimal lipophilicity range, along with low molecular weight and small polar surface area, are major prerequisites that lead to good absorption of chemicals by the intestine through passive diffusion [48,49]. For this reason, an assessment of drug-likeness was made, and various physicochemical properties were calculated for all tested compounds (Table 1 and Supplementary Materials).

All compounds had relatively low molecular weights within the range of 223–352 g·mol^−1^. Their polar surface areas are low and well within the range where good central nervous system (CNS) penetration is plausible [50,51]. The latter parameter is especially important to treat the CNS-persistent second stage of human African trypanosomiasis (HAT) [52]. All derivatives fulfill the Lipinski rule of five [53] and the Veber rule [54]. Except for imides, **13c** and **13g**, the logP data of which lie slightly outside the proposed region, all mentioned compounds also comply with the drug-likeness classifier defined by Ghose et al. [55].

The adequate application of ligand efficiency (LE) metrics is of utmost relevance in guiding lead discovery—and, more importantly, lead optimization—towards drug-like chemical space [56,57]. The ligand efficiency metrics of our synthesized compounds (LE > ~0.3), lipophilic ligand efficiency (LLE) (LLE > ~5), and lipophilicity-corrected ligand efficiency (LELP) (−10 < LELP < 10) agree closely with the values proposed for drug candidates [56] (see Supplementary Materials, Table S3).

In addition, all synthesized dienones were subjected to the BOILED-Egg analysis [58,59], an improvement upon the well-known Egan egg model [60]. This tool utilizes the computed lipophilicity and polarity of small drugs as input, allowing researchers to predict brain and intestinal permeation efficacies. This model is frequently used in industrial and academic contexts for drug discovery and development. All computed substances are within the thresholds of the model and, therefore, are predicted to show good gastrointestinal absorption. Furthermore, compounds, **13d** and **14a**, also lie within the physicochemical space of molecules that have a high probability of permeating the blood-brain barrier (BBB) (see Supplementary Materials, Figure S1).

### 2.3. Biological Evaluation

#### 2.3.1. Antiproliferative Activity

**Figure 2:**
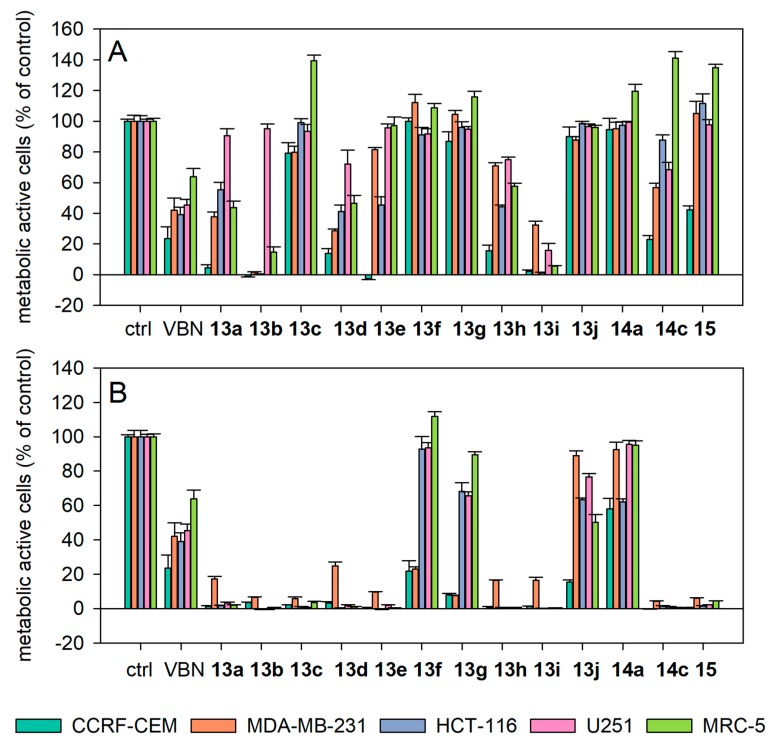
Results of the XTT^a^ viability assay using leukemia (CCRF-CEM), breast cancer (MDA-MB-231), colon cancer (HCT-116), and glioblastoma cells (U251) as well as non-tumorigenic lung fibroblasts (MRC-5). Cells were treated with 5 µg/mL (**A**) or 50 µg/mL (**B**) of the derivatives for 72 h. Afterwards, the metabolic activities of the cells were measured. Vinblastine (VBN) served as the positive control (0.01 µg/mL). The results are expressed as a percentage of vehicle-treated (0.5% DMSO) control cells (ctrl) (mean ± s.e.m., *n* = 6). ^a^ (2,3-Bis-(2-methoxy-4-nitro-5-sulfophenyl)-2H-tetrazolium-5-carboxanilide).

All synthesized dienones (**13a***–***j**, **14a**, **14c**, and **15**) were evaluated for their cytotoxic activity against human (cancer) cell lines. To cover a range of different tumor entities, we used a panel of cancer cell lines (CCRF-CEM leukemia, MDA-MB-231 breast cancer, HCT-116 colon cancer, and U251 glioblastoma cells) as well as a non-tumorigenic human cell line (MRC-5 lung fibroblasts). All derivatives were screened for their cytotoxicity at 5 µg/mL and 50 µg/mL to cover a broad concentration range. Cells were exposed to the derivatives for 72 h. As can be seen in Figure 2, the highest cytotoxicity was found for **13b** and **13i**. Both compounds reduced the metabolic activity below 40% of the control at 5 µg/mL in all (**13i**) or almost all (**13b**) cell lines. At 50 µg/mL, almost all tested compounds displayed activities against leukemia and breast cancer cells lines, but the overall cytotoxicity of compounds **13f**, **13g**, **13j**, and **14a** was negligible. Furthermore, the 50 µg/mL dilution corresponds to concentrations in the 140–215 µM range, which is not considered to be active. Interestingly, the morpholine derivative, **14c**, displayed only minor cytotoxicity against most cell lines at 5 µg/mL, but subsequently exhibited the highest antitrypanosomal activity. No clear correlation between cytotoxic and antiprotozoal effects could be drawn. Against human MRC-5 cells, **13f**, **13g**, **13j**, and **14a** did not show any cytotoxicity at 5 µg/mL and only a weak activity at 50 µg/mL. These results essentially match those of the subsequent L6 cytotoxicity assay, which was performed during the antiprotozoal screening. In that series, the highest IC_50_ values (and, therefore, the lowest cytotoxicity) were found for compounds **13b**, **13f**, and **13g** (for detailed results, see Supplementary Materials, Table S4).

#### 2.3.2. Antiprotozoal Activity

The synthesized dienones were also investigated for their activity against *P. falciparum* NF54 and T. brucei rhodesiense STIB900 as well as for their cytotoxicity against L6 rat skeletal myoblasts (Table 1). For each parasite, a selectivity index (SI = IC_50(L6)_/IC_50(parasite)_) was calculated. The TDR (Special Program for Research and Training in Tropical Diseases, World Health Organization) criteria [61] were adopted to interpret antiparasitic activity and selectivity.

All derivatives showed moderate (IC_50_ = 1–10 μM) or high (IC_50_ < 1 μM) activity towards *T. brucei rhodesiense*, except the inactive pyridine-2,3-dicarboximide, **13f**, and the morpholine-3,5-dione, **13g**, with IC_50_ values >10 μM. These results were surprising, especially in the case of compound **13f**, as basic, nitrogen-containing compounds often show favorable activities against protozoal parasites. Interestingly, the corresponding morpholine derivative, **14c**, showed, in contrast, the highest antitrypanosomal activity (IC_50_ = 0.27 μM) of all the tested *N*-dienones. Compared to the antitrypanosomal activity, the antiplasmodial effects of all tested compounds against *P. falciparum* NF54 strains were rather weak. Notable in this series, unfortunately, was the lack of selectivity of most compounds, as the compounds showed selectivity indices (SI) of <13.

#### 2.3.3. Structure-Activity Relationships (SAR) of the Antiproliferative and Antiprotozoal Activity

The mechanism of action of compounds with quinoid structural elements is based on redox cycling with excessive generation of reactive oxygen species (ROS) in the intracellular environment [62]. ROS play central roles in cell signaling and are able to activate the intrinsic pathway of cell apoptosis [63]. It is expected that these processes are responsible for the cytotoxic action of quinoids on microorganisms, as well as on tumor cells [64]. Due to the nature of the presented compounds, target specific effects are few and far between. For instance, the antiproliferative effects of the phytoquinoid, jacaranone, are caused by its interactions with the protein kinase B (AKT) and mitogen-activated protein kinase (p38 MAPK) signaling pathways [13].

**Figure 3:**
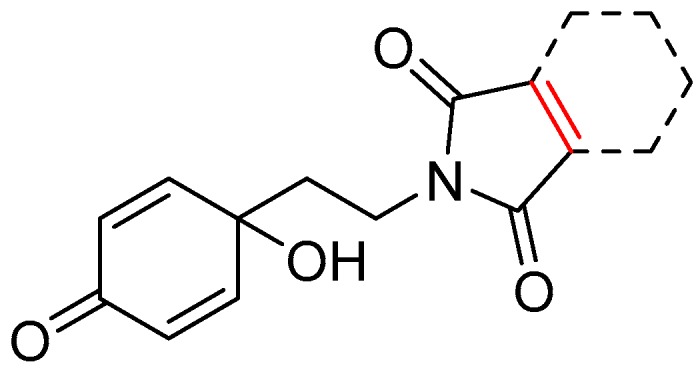
Crucial scaffold for antiproliferative activity.

Nevertheless, a closer examination of the SARs of jacaranone-based nitrogenous cyclohexadienones with regard to their antiproliferative activity (Figure 2) suggests that the most potent compounds, **13b**, **13e**, and **13i**, share an α,β-unsaturated imide as a core structural element (Figure 3).

These compounds showed high activity levels against some (**13b**: CCRF-CEM, MDA-MB-231, HCT-116; **13e**: CCRF-CEM) or all (**13i**) tested cell lines at low concentrations. Compound **13b** also showed a comparably low cytotoxicity against L6 cells with an IC_50_ of 26.8 µM, hinting that it has a target-specific inhibitory effect. In contrast, pyridine-2,3-dicarboximide, **13f**, and the morpholine-3,5-dione, **13g,** exhibited neither cytotoxic nor antiprotozoal activity.

Unfortunately, our antiprotozoal assays revealed no specific effects. However, a certain rank correlation between antiplasmodial activity and logP (r_S_ = −0.772) or ASApho values (r_S_ = −0.711) on one side and antitrypanosomal activity and ASApol (r_S_ = 0.870) on the other side was observed (see also Supplementary Materials, Table S2).

## 3. Experimental Section

### 3.1. Chemicals and Instruments

Melting points were obtained on a digital melting point apparatus (Electrothermal IA 9200, Staffordshire, UK). The NMR spectra were measured on a Unity Inova 400 MHz instrument (Varian, Darmstadt, Germany) and a Avance III 300 MHz NMR Spectrometer (Bruker, Rheinstetten, Germany) at 25 °C using 5 mm tubes. Chemical shifts were given in parts per million (ppm), the tetramethylsilane (TMS) resonance (0.00 ppm) was used as an internal standard. Coupling constants (*J*) were reported in hertz (Hz). ^1^H and ^13^C-resonances were assigned using ^1^H,^1^H, and ^1^H,^13^C correlation spectra. ^1^H and ^13^C resonances are numbered as given in the formulae (see Supplementary Materials).

High-resolution EI mass spectra (70 eV, source temperature 220 °C) were recorded on an orthogonal TOF spectrometer (Waters GCT Premier, Milford, MA, USA) equipped with a direct insertion (DI) probe. Typically, 0.2 µL of a solution of the sample (c = 0.1 mg/mL) were placed in the glass cup used for DI, dried under atmospheric pressure, and transferred into the vacuum. Mass spectra (50–800 Da; 1 spectrum/s; resolution appr. 7500 FWHM) were continuously acquired while the sample was evaporated rapidly. ESI mass spectra were acquired on an Exactive Orbitrap mass spectrometer equipped with a heated ESI II source (ThermoFisher Scientific, Inc., Bremen, Germany).

HPLC separations were performed on an Agilent HPLC instrument 1200 series (Santa Clara, CA, USA) with quaternary pump, autosampler, autoinjector, column oven, and DAD detection. A Eurospher C18 column (particle size 1.8 μm; 2.0 × 125 mm with guard cartridge) (Knauer, Berlin, Germany) was used for analysis of the compounds at a flow rate of 150 µL/min and at a constant temperature of 25 °C. The chromatographic method was performed with a gradient of acetonitrile (A) in millipore water (B), both with each 0.1% HCOOH, from 10% to 90% A in B within 20 min, then to 100% A within 5 min, followed by returning to starting conditions within 1 min, and re-equilibration for 8 min. 5.0 µL of sample dissolved in methanol were injected and detection was done at 205, 220, and 254 nm. As assayed through HPLC-DAD analysis, all tested compounds possessed a purity higher than 95%.

Materials: TLC was carried out on TLC plates (silica gel 60 *F*_254_ 0.2 mm, 200 × 200 mm) (Merck, Darmstadt, Germany). TLCs were visualized by spraying with cerium(IV) sulfate/ammonium molybdate and subsequent heating with a heat gun. The phosphate buffer (1 M, pH = 6.4) was prepared as follows: 7.1 g (0.05 mmol) Na_2_HPO_4_ and 6.9 g (0.05 mmol) NaH_2_PO_4_ × H_2_O was dissolved in H_2_O and diluted to 100 mL with the same solvent. The pH was controlled using a pH-meter and, if necessary, adjusted to a pH of 6.4. Solvents were concentrated by rotary evaporation below 50 °C. Purity and homogeneity of compounds were assessed by the TLC and HPLC methods.

The intermediates, **6a**–**c**, were prepared via Mitsunobu reaction according to the literature [22], and the subsequent deprotection step was accomplished with TBAF according to the literature [24]. Proton sponge^®^ (1,8-bis(dimethylamino)naphthalene) and other chemicals were purchased from Sigma-Aldrich (Vienna, Austria). All reagents and chemicals were used without any further purification.

### 3.2. Synthesis

*2-[4-(Thexyldimethylsilyloxy)phenyl]ethanol* (**5**). To a stirred solution of methyl 4-hydroxyphenylacetate **4** (1.2 g, 7.2 mmol) in anhydrous CH_2_Cl_2_ (12 mL), 1.3 mL 1,8-diazabicyclo[5.4.0]undec-7-ene (8.7 mmol) and 1.6 mL thexyldimethylsilyl chloride (8.0 mmol) were added successively at 0 °C. The mixture was allowed to reach ambient temperature until TLC showed complete consumption of the starting material (1.5 h). The reaction mixture was diluted with H_2_O and extracted three times with EtOAc. The combined organic layers were dried over Na_2_SO_4_ and evaporated to dryness. The crude product was purified by flash chromatography using cyclohexane (CH)/EtOAc (3:1) to obtain 2-[4-(thexyldimethylsilyloxy)phenyl]-acetate in quantitative yield as a colourless oil.

The residual oil was dissolved in 12 mL of anhydrous THF and then slowly treated with 7.2 mL LiAlH_4_ (1 M solution in THF) at 0 °C. After the addition of LiAlH_4_ was completed, the reaction was allowed to reach ambient temperature for 2 h. A 2 M aqueous solution of Na/K tartrate (40 mL) was slowly added at 0 °C to quench the reaction and the mixture was stirred for 1 h. Then, the solution was extracted three times with MTBE, the combined organic layers were dried over Na_2_SO_4_, and concentrated in vacuo to yield 2.0 g (98%) of crude **5** as a clear, colourless oil, which was used without further purification. *R*_f_ = 0.27 (CH:EtOAc = 2:1); ^1^H-NMR (400 MHz, CDCl_3_) δ 7.08 (d, *J* = 8.5 Hz, 2H, H-2/6), 6.78 (d, *J* = 8.5 Hz, 2H, H-3/5), 3.82 (t, *J* = 6.5 Hz, 2H, H-8), 2.80 (t, *J* = 6.5 Hz, 2H, H-7), 1.73 (hept, *J* = 6.9 Hz, 1H, CH-(CH_3_)_2_), 0.94 (d, *J* = 6.9 Hz, 6H, (CH_3_)_2_-CH), 0.94 (s, 6H, (CH_3_)_2_-C), 0.21 (s, 6H, (CH_3_)_2_-Si) ppm; ^13^C-NMR (100 MHz, CDCl_3_) δ 154.1 (C-4), 130.8 (C-1), 129.9 (C-2/6), 120.2 (C-3/5), 63.8 (C-8), 38.4 (C-7), 34.1 (CH-(CH_3_)_2_), 25.0 (C-(CH_3_)_2_), 20.1 ((CH_3_)_2_-C), 18.6 ((CH_3_)_2_-CH), −2.5 ((CH_3_)_2_-Si) ppm; HRMS (ESI) calcd. for C_16_H_29_O_2_Si [M + H]^+^ = 281.1937; Found: 281.1931.

#### 3.2.1. General Procedure for the Synthesis of the Compounds, **7a**–**7j**

*AcOH-assisted condensation*. A mixture of tyramine **8** (274 mg, 2.0 mmol) and the corresponding anhydride (1.9 mmol) in glacial acetic acid (3 mL) were refluxed for 1.5 h. After cooling of the reaction mixture to ambient temperature, cold H_2_O (10 mL) was added and the resultant precipitate was filtered, washed several times with cold water, and dried under reduced pressure (**7a**, **7e**). When the product did not precipitate from the solution (**7b**–**d**, **7h**–**j**), H_2_O (15 mL) was added, and the aqueous phase was extracted several times with EtOAc. The combined organic layers were washed with 1 M NaHCO_3_, dried over Na_2_SO_4_, and concentrated in vacuo to yield the crude products, which were used without further purification.

*PEG 400-assisted condensation*. A mixture of tyramine **8** (274 mg, 2.0 mmol) and the corresponding anhydride (1.9 mmol) in PEG 400 (0.3 mL) was heated under stirring at 140 °C for 4 h. After cooling to ambient temperature, a large quantity of ice-water (~30 mL) was added. The resultant precipitate was filtered, washed several times with cold water, and dried under reduced pressure (**7a**, **7e**). For **7b**–**d** and **7f**–**j**, the aqueous phase was extracted several times with EtOAc, the combined organic layers were dried over Na_2_SO_4_, and evaporated to dryness. The crude products were purified by flash chromatography (**7a**–**c** and **7e**–**j**) or recrystallization from EtOAc/acetone/EtOH (30:5:5) (**7d**).

*4-(Thexyldimethylsilyloxy)phenethyl bromide* (**9**). To a stirred solution of PPh_3_ (1.3 g, 5.0 mmol) in anhydrous CH_2_Cl_2_ (10 mL), DDQ (1.1 g, 5.0 mmol) was added slowly at room temperature. Then, (*n*-butyl)_4_NBr (1.6 g, 5.0 mmol) and 1.2 g (4.2 mmol) of alcohol **5** (dissolved in an additional 5 mL of anhydrous CH_2_Cl_2_) were added in 10 min intervals to the thick, beige-coloured mixture. After the addition of **5**, the colour of the reaction mixture immediately changed to deep red. The reaction was stirred for 50 min at an ambient temperature until TLC showed complete consumption of the starting material. The solvent was evaporated to dryness, and the crude product was purified by flash chromatography using CH/EtOAc (1:1) to obtain 1.4 g (81%) of **9** as yellow oil. *R*_f_ = 0.70 (CH:EtOAC = 1:1); ^1^H-NMR (400 MHz, CDCl_3_) δ 7.05 (d, *J* = 8.4 Hz, 2H, H-2/6), 6.77 (d, *J* = 8.5 Hz, 2H, H-3/5), 3.52 (t, *J* = 7.8 Hz, 2H, H-8), 3.08 (t, *J* = 7.8 Hz, 2H, H-7), 1.72 (hept, *J* = 6.9 Hz, 1H, CH-(CH_3_)_2_), 0.94 (d, *J* = 6.9 Hz, 6H, (CH_3_)_2_-CH), 0.94 (s, 6H, (CH_3_)_2_-C), 0.21 (s, 6H, (CH_3_)_2_-Si) ppm; ^13^C-NMR (100 MHz, CDCl_3_) δ 154.4 (C-4), 131.5 (C-1), 129.6 (C-2/6), 120.2 (C-3/5), 38.8 (C-7), 34.1 (CH-(CH_3_)_2_), 33.3 (C-8), 25.0 (C-(CH_3_)_2_), 20.1 ((CH_3_)_2_-C), 18.6 ((CH_3_)_2_-CH), −2.5 ((CH_3_)_2_-Si) ppm; HRMS (EI) Calcd. for C_16_H_27_SiOBr [M]^+^ = 342.1014; Found: 342.1017.

#### 3.2.2. General Procedure for the Synthesis of the Compounds, **11a**–**11d**

A. Nitrogen alkylation

*Conventional method*. 6 mmol of the respective secondary amine (**11a:** isoindoline, **11b:** pyrrolidine, **11c:** morpholine, **11d:** octahydroisoindole) were dissolved in anhydrous EtOH (3 mL). Then, 206 mg (0.6 mmol) of **9** (dissolved in 1 mL anhydrous EtOH) were added and the mixture was refluxed for 72 h. The solvent was evaporated to dryness to give a residue (**10a**–**10d**), which was used in the following deprotection step without further purification.

*Proton-sponge^®^ method*. 275 mg (0.8 mmol) of **9** (dissolved in 4 mL anhydrous CH_3_CN) were mixed with a stirred solution of 120 mg (0.8 mmol) of NaI and 171 mg (0.8 mmol) of proton-sponge^®^ in anhydrous CH_3_CN (3 mL). Then, 1.6 mmol of the respective secondary amine were added and the mixture was refluxed for 20 h. The solvent was evaporated to dryness to give a residue (**10a**–**10d**), which was used in the following deprotection step without further purification.

B. Removal of the TDS-protecting group

LiOH-hydrate (126 mg, 3.0 mmol) was added to a solution of the TDS ether (**10a**–**d**, 1 mmol) in anhydrous DMF (2 mL) and the mixture was stirred at ambient temperature until TLC showed complete consumption of the starting material (3–17 h). The reaction mixture was then diluted with H_2_O (15 mL), neutralised with phosphate buffer (pH = 6.4), and extracted several times with EtOAc. The combined organic layers were dried over Na_2_SO_4_ and concentrated in vacuo to give a residue, which was purified by flash chromatography.

*N-(4-Hydroxyphenethyl)isoindoline* (**11a**). Compound **11a** was prepared from **9** via **10a** as a white amorphous solid and purified by flash chromatography using CH/EtOAc (1:3). Yield: 60% (proton-sponge^®^ method), 0% (conventional method); *R*_f_ = 0.30 (CH:EtOAC = 1:3); ^1^H-NMR (400 MHz, DMSO-*d*_6_) δ 9.15 (s, 1H, 4-OH), 7.24–7.16 (m, 4H, ArH), 7.05 (d, *J* = 8.4 Hz, 2H, H-2/6), 6.67 (d, *J* = 8.3 Hz, 2H, H-3/5), 3.87 (s, 4H, CH_2_-N), 2.86–2.81 (m, 2H, H-8), 2.69 (t, *J* = 7.7 Hz, 2H, H-7) ppm; ^13^C-NMR (100 MHz, DMSO-*d*_6_) δ 155.9 (C-4), 140.5 (ArC), 130.7 (C-1), 129.9 (C-2/6), 127.0 (ArC), 122.6 (ArC), 115.4 (C-3/5), 58.9 (CH_2_-N), 57.9 (C-8), 34.3 (C-7) ppm; HRMS (EI) calcd. for C_16_H_17_NO [M]^+^ = 239.1310; Found: 239.1303.

*N-(4-Hydroxyphenethyl)pyrrolidine* (**11b**). Compound **11b** was prepared from **9** via **10b** as a white amorphous solid and purified by flash chromatography using CHCl_3_/MeOH (1:1). Yield: 81% (proton-sponge^®^ method), 86% (conventional method); *R*_f_ = 0.22 (CHCl_3_:MeOH = 1:1); ^1^H-NMR (400 MHz, CDCl_3_) δ 6.99 (d, *J* = 8.3 Hz, 2H, H-2/6), 6.63 (d, *J* = 8.3 Hz, 2H, H-3/5), 2.75 (s, 4H, H-7/8), 2.69–2.62 (m, 4H, CH_2_-N), 1.88–1.79 (m, 4H, CH_2_-CH_2_-N) ppm; ^13^C-NMR (100 MHz, CDCl_3_) δ 155.2 (C-4), 130.6 (C-1), 129.5 (C-2/6), 115.7 (C-3/5), 58.6 (C-8), 54.0 (CH_2_-N), 34.2 (C-7), 23.3 (CH_2_-CH_2_-N) ppm; HRMS (EI) calcd. for C_12_H_17_NO [M]^+^ = 191.1310; Found: 191.1304.

*N-(4-Hydroxyphenethyl)morpholine* (**11c**). Compound **11c** was prepared from **9** via **10c** as a white amorphous solid and purified by flash chromatography using CHCl_3_/MeOH (15:1). Yield: 87% (proton-sponge^®^ method), 87% (conventional method); *R*_f_ = 0.27 CHCl_3_:MeOH (15:1); ^1^H-NMR (400 MHz, DMSO-*d*_6_) δ 9.14 (s, 1H, 4-OH), 6.99 (d, *J* = 8.5 Hz, 2H, H-2/6), 6.65 (d, *J* = 8.5 Hz, 2H, H-3/5), 3.56 (t, *J* = 4.6 Hz, 4H, CH_2_-O), 2.62–2.57 (m, 2H, H-7), 2.44–2.39 (m, 2H, H-8), 2.41–2.35 (m, 4H, CH_2_-N) ppm; ^13^C-NMR (100 MHz, DMSO-*d*_6_) δ 155.9 (C-4), 130.7 (C-1), 129.9 (C-2/6), 115.5 (C-3/5), 66.6 (CH_2_-O), 61.1 (C-8), 53.8 (CH_2_-N), 32.1 (C-7) ppm; HRMS (EI) calcd. for C_12_H_17_NO_2_ [M]^+^ = 207.1259; Found: 207.1255.

*N-(4-Hydroxyphenethyl)octahydroisoindole* (**11d**). Compound **11d** was prepared from **9** via **10d** as a white amorphous solid and purified by flash chromatography using CHCl_3_/EtOH (5:2). Yield: 77% (proton-sponge^®^ method), 79% (conventional method); *R*_f_ = 0.19 (CHCl_3_:EtOH = 5:2); ^1^H-NMR (400 MHz, CDCl_3_) δ 6.93 (d, *J* = 8.3 Hz, 2H, H-2/6), 6.77 (d, *J* = 8.3 Hz, 2H, C-3/5), 3.18 (dd, *J* = 10.7, 6.3 Hz, 2H, CH_2(a)_-N), 3.06–3.00 (m, 2H, H-8), 2.93–2.87 (m, 2H, CH_2(b)_-N), 2.86–2.80 (m, 2H, H-7), 2.34–2.24 (m, 2H, CH-CH_2_), 1.67–1.58 (m, 2H, CH_2(a)_-CH), 1.54–1.45 (m, 2H, CH_2(a)_-CH_2_-CH), 1.52–1.43 (m, 2H, CH_2(b)_-CH), 1.39–1.31 (m, 2H, CH_2(b)_-CH_2_-CH) ppm; ^13^C-NMR (100 MHz, CDCl_3_) δ 156.4 (C-4), 129.5 (C-2/6), 128.1 (C-1), 115.9 (C-3/5), 59.0 (C-8), 57.1 (CH_2_-N), 36.7 (CH-CH_2_), 32.3 (C-7), 25.9 (CH_2_-CH), 22.5 (CH_2_-CH_2_-CH) ppm; HRMS (EI) calcd. for C_16_H_23_NO [M]^+^ = 245.1780; Found: 245.1772.

*N-(4-Hydroxyphenethyl)-4,5,6,7-tetrahydroisoindole* (**11e**). A mixture of **11a** (239 mg, 1 mmol), ammonium formate (631 mg, 10 mmol), and palladium hydroxide on carbon, 20 wt. % loading (64 mg) in anhydrous MeOH (8 mL) was refluxed for 16 hrs. The mixture was filtered through celite^®^, diluted with H_2_O, and extracted three times with EtOAc. The combined organic layers were dried over Na_2_SO_4_ and concentrated in vacuo to give 241 mg of crude **11e** as a slightly yellow oil in quantitative yields, which can be used without further purification. *R*_f_ = 0.69 (EtOAc); ^1^H-NMR (400 MHz, CDCl_3_) δ 7.00 (d, *J* = 8.2 Hz, 2H, H-2/6), 6.75 (d, *J* = 8.2 Hz, 2H, H-3/5), 6.31 (s, 2H, CH-N), 3.97–3.91 (m, 2H, H-8), 3.01–2.90 (m, 2H, H-7), 2.59–2.52 (m, 4H, CH_2_-C=), 1.76–1.68 (m, 4H, CH_2_-CH_2_-C=) ppm; ^13^C-NMR (100 MHz, CDCl_3_) δ 154.3 (C-4), 130.7 (C-1), 129.8 (C-2/6), 119.4 (C=C(H)-N), 115.9 (CH-N), 115.4 (C-3/5), 51.3 (C-8), 37.6 (C-7), 24.2 (CH_2_-CH_2_-C=), 22.0 (CH_2_-C=) ppm; HRMS (EI) calcd. for C_16_H_19_NO [M]^+^ = 241.1467; Found: 241.1465.

#### 3.2.3. General Procedure for the Reduction of Heterocyclic N-Imides

The corresponding imide, **7a/7h**, (2 mmol) was dissolved in 8 mL of anhydrous THF and then treated dropwise with 4 mL LiAlH_4_ (1 M solution in THF) at 0 °C. After the addition of LiAlH_4_ was complete, the reaction was allowed to reach an ambient temperature for 2 h. Then, a 2 M aqueous solution of Na/K tartrate (50 mL) was added at 0 °C to quench the reaction and the mixture was stirred for 1 h. The solution was extracted four times with MTBE, the combined organic layers were dried over Na_2_SO_4_, and concentrated in vacuo to give a residue, which was purified by flash chromatography.

*N-(4-Hydroxyphenethyl)isoindoline* (**11a**). Compound **11a** was prepared from **7a** as a white amorphous solid and purified by flash chromatography using CH/EtOAc (1:3). Yield: 72%; *R*_f_ = 0.30 (CH:EtOAC = 1:3).

*3-Hydroxy-N-(4-hydroxyphenethyl)octahydroisoindole-1-one* (**12**). Compound **12** was prepared from **7h** as a white amorphous solid and purified by flash chromatography using CH/EtOAc (1:3). Yield: 88%; *R*_f_ = 0.28 (CH:EtOAc = 1:3); HRMS (EI) calcd. for C_16_H_21_NO_3_ [M]^+^ = 275.1521; Found: 275.1527.

#### 3.2.4. Procedures for the Synthesis of Dienones, **13a**–**j**, **14a**, **14c**, **15**

*Conventional method*. A solution of the cyclic imide, **7a**–**j,** or amide, **12**, (1.5 mmol) in CH_3_CN (20 mL) and H_2_O (8 mL) at 0 °C was treated with PIDA (644 mg, 2.0 mmol), and stirred for 7 min at this temperature. The reaction mixture was diluted with EtOAc and washed with a 1 M aqueous solution of NaHCO_3_. The aqueous phase was re-extracted three times with EtOAc. The combined organic layers were dried over Na_2_SO_4_ and concentrated in vacuo to give a residue, which was purified by flash chromatography.

*Phosphate buffer method*. 1 mmol of the respective tertiary amine, **11a/11c**, was dissolved in 1.5 mL HCl (1 M in dioxane) and concentrated in vacuo to yield the corresponding hydrochloride. A solution of the hydrochloride in CH_3_CN (12 mL), H_2_O (3 mL) and phosphate buffer (1 M, pH = 6.4, 2 mL) at 0 °C was then treated with PIDA (644 mg, 2.0 mmol) and stirred for 7 min at this temperature. The solvent was evaporated to dryness, and the crude product was purified by flash chromatography.

*N-[2-(1-Hydroxy-4-oxocyclohexa-2,5-dien-1-yl)ethyl]phthalimide* (**13a**). Compound **13a** was prepared from **7a** as white crystals and purified by flash chromatography using CH/EtOAc (1:3). Yield: 67%; *R*_f_ = 0.42 (CH:EtOAc = 1:3); m.p.: 161–162 °C; ^1^H-NMR (300 MHz, DMSO-*d*_6_) δ 7.88–7.80 (m, 4H, ArH), 6.97 (d, *J* = 10.2 Hz, 2H, H-2/6), 6.10 (d, *J* = 10.2 Hz, 2H, H-3/5), 5.88 (s, 1H, 1-OH), 3.68–3.50 (m, 2H, H-8), 2.08–1.88 (m, 2H, H-7) ppm; ^13^C-NMR (100 MHz, DMSO-*d*_6_) δ 185.0 (C-4), 167.7 ((CO)N), 152.2 (C-2/6), 134.4 (ArC), 131.7 (ArC), 127.1 (C-3/5), 123.0 (ArC), 67.5 (C-1), 37.8 (C-7), 33.1 (C-8) ppm; HRMS (EI) calcd. for C_16_H_13_NO_4_ [M]^+^ = 283.0845; Found: 283.0845.

*N-[2-(1-Hydroxy-4-oxocyclohexa-2,5-dien-1-yl)ethyl]maleimide* (**13b**). Compound **13b** was prepared from **7b** as yellow crystals and purified by flash chromatography using CH/EtOAc (1:5). Yield: 17%; *R*_f_ = 0.40 (CH:EtOAc = 1:5); m.p.: 151–152 °C; ^1^H-NMR (300 MHz, DMSO-*d*_6_) δ 6.99 (s, 2H, CH-(CO)N), 6.91 (d, *J* = 10.1 Hz, 2H, H-2/6), 6.08 (d, *J* = 10.1 Hz, 2H, H-3/5), 5.85 (s, 1H, 1-OH), 3.45–3.38 (m, 2H, H-8), 1.93–1.85 (m, 2H, H-7) ppm; ^13^C-NMR (100 MHz, DMSO-*d*_6_) δ 185.0 (C-4), 170.8 ((CO)N), 152.1 (C-2/6), 134.6 (CH-(CO)N), 127.1 (C-3/5), 67.4 (C-1), 37.9 (C-7), 32.8 (C-8) ppm; HRMS (EI) calcd. for C_12_H_11_NO_4_ [M]^+^ = 233.0688; Found: 233.0686.

*N-[2-(1-Hydroxy-4-oxocyclohexa-2,5-dien-1-yl)ethyl]succinimide* (**13c**). Compound **13c** was prepared from **7c** as a white solid and purified by flash chromatography using CHCl_3_/CH_3_CN (1:3). Yield: 55%; *R*_f_ = 0.51 (CHCl_3_:CH_3_CN = 1:3); m.p.: 128–129 °C; ^1^H-NMR (400 MHz, DMSO-*d*_6_) δ 6.93 (d, *J* = 10.2 Hz, 2H, H-2/6), 6.10 (d, *J* = 10.2 Hz, 2H, H-3/5), 5.86 (s, 1H, 1-OH), 2.57 (s br, 4H, CH_2_-(CO)N), 3.38–3.31 (m, 2H, H-8), 1.89–1.77 (m, 2H, H-7) ppm; ^13^C-NMR (100 MHz, DMSO-*d*_6_) δ 185.5 (C-4), 178.0 ((CO)N), 152.6 (C-2/6), 127.5 (C-3/5), 67.9 (C-1), 37.5 (C-7), 33.9 (C-8), 28.4 (CH_2_-(CO)N) ppm; HRMS (EI) calcd. for C_12_H_13_NO_4_ [M]^+^ = 235.0845; Found: 235.0826.

*4,5-Dichloro-N-[2-(1-hydroxy-4-oxocyclohexa-2,5-dien-1-yl)ethyl]phthalimide* (**13d**). Compound **13d** was prepared from **7d** as white crystals and purified by flash chromatography using CH/EtOAc (1:1). Yield: 40%; *R*_f_ = 0.22 (CH:EtOAc = 1:1); m.p.: 213–214 °C; ^1^H-NMR (400 MHz, DMSO-*d*_6_) δ 8.17 (s, 2H, ArH), 6.96 (d, *J* = 10.1 Hz, 2H, H-2/6), 6.10 (d, *J* = 10.1 Hz, 2H, H-3/5), 5.89 (s, 1H, 1-OH), 3.62–3.56 (m, 2H, H-8), 2.02–1.95 (m, 2H, H-7) ppm; ^13^C-NMR (100 MHz, DMSO-*d*_6_) δ 185.5 (C-4), 166.4 ((CO)N), 152.6 (C-2/6), 137.7 (C(Cl)=), 132.1 (C=C(CO)N), 127.6 (C-3/5), 125.6 (ArC), 67.9 (C-1), 38.0 (C-7), 34.0 (C-8) ppm; HRMS (EI) calcd. for C_16_H_11_Cl_2_NO_4_ [M]^+^ = 351.0065; Found: 351.0090.

*3,4-Dichloro-N-[2-(1-hydroxy-4-oxocyclohexa-2,5-dien-1-yl)ethyl]maleimide* (**13e**). Compound **13e** was prepared from **7e** as yellowish crystals and purified by flash chromatography using CH/EtOAc (1:1). Yield: 64%; *R*_f_ = 0.30 (CH:EtOAc = 1:1); m.p.: 168–169 °C; ^1^H-NMR (400 MHz, DMSO-*d*_6_) δ 6.95 (d, *J* = 10.1 Hz, 2H, H-2/6), 6.11 (d, *J* = 10.1 Hz, 2H, H-3/5), 5.91 (s br, 1H, 1-OH), 3.53–3.46 (m, 2H, H-8), 1.95–1.90 (m, 2H, H-7) ppm; ^13^C-NMR (100 MHz, DMSO-*d*_6_) δ 185.4 (C-4), 163.3 ((CO)N), 152.5 (C-2/6), 132.9 (C(Cl)=), 127.6 (C-3/5), 67.8 (C-1), 37.9 (C-7), 34.8 (C-8) ppm; HRMS (EI) calcd. for C_12_H_9_Cl_2_NO_4_ [M]^+^ = 300.9909; Found: 300.9914.

*N-[2-(1-Hydroxy-4-oxocyclohexa-2,5-dien-1-yl)ethyl]pyridine-2,3-dicarboximide* (**13f**). Compound **13f** was prepared from **7f** as white crystals and purified by flash chromatography using CHCl_3_/CH_3_CN (1:1). Yield: 19%; *R*_f_ = 0.37 (CHCl_3_:CH_3_CN = 1:1); m.p.: 167–168 °C; ^1^H-NMR (400 MHz, DMSO-*d*_6_) δ 8.95 (dd, *J* = 5.0, 1.5 Hz, 1H, ArH), 8.27 (dd, *J* = 7.7, 1.5 Hz, 1H, ArH), 7.77 (dd, *J* = 7.7, 5.0 Hz, 1H, ArH), 6.98 (d, *J* = 10.1 Hz, 2H, C-2/6), 6.11 (d, *J* = 10.1, 2H, H-3/5), 5.88 (s, 1H, 1-OH), 3.69–3.57 (m, 2H, C-8), 2.05–1.94 (m, 2H, H-7) ppm; ^13^C-NMR (100 MHz, DMSO-*d*_6_) δ 185.5 (C-4), 166.6 ((CO)N), 155.2 (ArC), 152.6 (C-2/6), 152.0 (ArC), 131.6 (ArC), 128.3 (ArC), 127.7 (ArC), 127.6 (C-3/5), 68.0 (C-1), 38.1 (C-7), 33.7 (C-8) ppm; HRMS (EI) calcd. for C_15_H_12_N_2_O_4_ [M]^+^ = 284.0797; Found: 284.0792.

*N-[2-(1-Hydroxy-4-oxocyclohexa-2,5-dien-1-yl)ethyl]morpholine-3,5-dione* (**13g**). Compound **13g** was prepared from **7g** as a yellowish solid and purified by flash chromatography using CH/EtOAc (1:5). Yield: 18%; *R*_f_ = 0.33 (CHCl_3_:EtOAc = 1:5); m.p.: 142–143 °C; ^1^H-NMR (400 MHz, DMSO-*d*_6_) δ 6.94 (d, *J* = 10.1 Hz, 2H, H-2/6), 6.12 (d, *J* = 10.0 Hz, 2H, H-3/5), 4.36 (s, 4H, CH_2_-(CO)N), 3.70–3.56 (m, 2H, H-8), 1.89–1.78 (m, 2H, H-7) ppm; ^13^C-NMR (100 MHz, DMSO-*d*_6_) δ 185.5 (C-4), 170.1 ((CO)N), 152.7 (C-2/6), 127.5 (C-3/5), 68.0 (C-1), 67.4 (CH_2_-(CO)N), 37.8 (C-7), 33.8 (C-8) ppm; HRMS (EI) calcd. for C_12_H_13_NO_5_ [M]^+^ = 251.0794; Found: 251.0794.

*N-[2-(1-Hydroxy-4-oxocyclohexa-2,5-dien-1-yl)ethyl]hexahydrophthalimide* (**13h**). Compound **13h** was prepared from **7h** as yellow crystals and purified by flash chromatography using CH/EtOAc (1:3). Yield: 79%; *R*_f_ = 0.29 (CH:EtOAc = 1:3); m.p.: 135–136 °C; ^1^H-NMR (400 MHz, DMSO-*d*_6_) δ 6.94 (d, *J* = 10.1 Hz, 2H, H-2/6), 6.10 (d, *J* = 10.1 Hz, 2H, H-3/5), 5.84 (s, 1H, 1-OH), 3.40–3.35 (m, 2H, H-8), 2.93–2.82 (m, 2H, CH-(CO)N), 1.85–1.80 (m, 2H, H-7), 1.71 (s, 2H, CH_2(a)_-CH), 1.60–1.51 (m, 2H, CH_2(b)_-CH), 1.42–1.32 (m, 2H, CH_2(a)_-CH_2_-CH), 1.31–1.21 (m, 2H, CH_2(b)_-CH_2_-CH) ppm; ^13^C-NMR (100 MHz, DMSO-*d*_6_) δ 185.4 (C-4), 179.7 ((CO)N), 152.7 (C-2/6), 127.5 (C-3/5), 67.9 (C-1), 39.3 (CH-(CO)N), 37.6 (C-7), 33.8 (C-8), 23.5 (CH_2_-CH), 21.6 (CH_2_-CH_2_-CH) ppm; HRMS (EI) calcd. for C_16_H_19_NO_4_ [M]^+^ = 289.1314; Found: 289.1310.

*N-[2-(1-Hydroxy-4-oxocyclohexa-2,5-dien-1-yl)ethyl]-3,4,5,6-tetrahydrophthalimide* (**13i**). Compound **13i** was prepared from **7i** as an orange solid and purified by flash chromatography using CH/EtOAc (1:1). Yield: 88%; *R*_f_ = 0.18 (CH:EtOAc = 1:1); m.p.: 93–94 °C; ^1^H-NMR (400 MHz, DMSO-*d*_6_) δ 6.92 (d, *J* = 10.1 Hz, 2H, H-2/6), 6.08 (d, *J* = 10.1 Hz, 2H, H-3/5), 5.85 (s, 1H, 1-OH), 3.42–3.36 (m, 2H, H-8), 2.24–2.17 (m, 4H, CH_2_-C=), 1.86 (dd, *J* = 8.5, 6.8 Hz, 2H, H-7), 1.69–1.62 (m, 4H, CH_2_-CH_2_-C=) ppm; ^13^C-NMR (100 MHz, DMSO-*d*_6_) δ 185.5 (C-4), 170.9 ((CO)N), 152.6 (C-2/6), 141.5 (C=C(CO)), 127.5 (C-3/5), 67.9 (C-1), 38.6 (C-7), 33.0 (C-8), 21.3 (CH_2_-CH_2_-C=), 19.9 (CH_2_-C=) ppm; HRMS (EI) calcd. for C_16_H_17_NO_4_ [M]^+^ = 287.1158; Found: 287.1160.

*N-[2-(1-Hydroxy-4-oxocyclohexa-2,5-dien-1-yl)ethyl]-1,2,3,6-tetrahydrophthalimide* (**13j**). Compound **13j** was prepared from **7j** as white crystals and purified by flash chromatography using CH/EtOAc (1:1). Yield: 49%; *R*_f_ = 0.28 (CH:EtOAc = 1:1); m.p.: 145–146 °C; ^1^H-NMR (400 MHz, DMSO-*d*_6_) δ 6.90 (d, *J* = 10.1 Hz, 2H, H-2/6), 6.09 (d, *J* = 10.1 Hz, 2H, H-3/5), 5.87 (s, 1H, 1-OH), 5.85–5.82 (m, 2H, CH=CH), 3.31–3.36 (m, 2H, H-8), 3.11–3.06 (m, 2H, CH-(CO)N), 2.39–2.32 (m, 2H, CH_2(a)_-CH), 2.21–2.13 (m, 2H, CH_2(b)_-CH), 1.79 (td, *J* = 7.5, 1.5 Hz, 2H, H-7) ppm; ^13^C-NMR (100 MHz, DMSO-*d*_6_) δ 185.5 (C-4), 180.3 ((CO)N), 152.5 (C-2/6), 128.1 (CH=CH), 127.6 (C-3/5), 67.8 (C-1), 38.9 (CH-(CO)N), 37.8 (C-7), 34.1 (C-8), 23.5 (CH_2_-CH) ppm; HRMS (EI) calcd. for C_16_H_17_NO_4_ [M]^+^ = 287.1158; Found: 287.1154.

*N-[2-(1-Hydroxy-4-oxocyclohexa-2,5-dien-1-yl)ethyl]isoindoline* (**14a**). Compound **14a** was prepared from **11a** applying the phosphate buffer method as a brownish solid and purified by flash chromatography using CHCl_3_/EtOH (1:5). Yield: 16%; *R*_f_ = 0.50 (CHCl_3_:EtOH = 1:5); m.p.: 103–104 °C; ^1^H-NMR (400 MHz, DMSO-*d*_6_) δ 7.24–7.12 (m, 4H, ArH), 6.99 (d, *J* = 10.1 Hz, 2H, H-2/6), 6.06 (d, *J* = 10.1 Hz, 2H, H-3/5), 3.81–3.77 (m, 4H, CH_2_-N), 2.70–2.62 (m, 2H, H-8), 1.93–1.85 (m, 2H, H-7) ppm; ^13^C-NMR (100 MHz, DMSO-*d*_6_) δ 185.3 (C-4), 153.2 (C-2/6), 139.8 (ArC), 126.5 (ArC), 126.3 (C-3/5) 122.0 (ArC), 68.0 (C-1), 58.3 (CH_2_-N), 50.0 (C-8), 38.6 (C-7) ppm; HRMS (EI) calcd. for C_16_H_17_NO_2_ [M]^+^ = 255.1259; Found: 255.1251.

*N-[2-(1-Hydroxy-4-oxocyclohexa-2,5-dien-1-yl)ethyl]morpholine* (**14c**). Compound **14c** was prepared from **11c** applying the phosphate buffer method as a brownish solid and purified by flash chromatography using EtOAc/EtOH (1:1). Yield: 28%; *R*_f_ = 0.37 (EtOAc:EtOH = 1:1); m.p.: 98–99 °C; ^1^H-NMR (400 MHz, DMSO-*d*_6_) δ 6.95 (d, *J* = 10.0 Hz, 2H, H-2/6), 6.04 (d, *J* = 10.0 Hz, 2H, H-3/5), 3.56–3.46 (m, 4H, CH_2_-O), 2.32–2.26 (m, 4H, CH_2_-N), 2.25–2.20 (m, 2H, H-8), 1.79 (t, *J* = 7.6 Hz, 2H, H-7) ppm; ^13^C-NMR (100 MHz, DMSO-*d*_6_) δ 185.8 (C-4), 153.8 (C-2/6), 126.8 (C-3/5), 68.5 (C-1), 66.6 (CH_2_-O), 53.7 (CH_2_-N), 53.4 (C-8), 37.0 (C-7) ppm; HRMS (EI) calcd. for C_12_H_17_NO_3_ [M]^+^ = 223.1208; Found: 223.1201.

*3-Hydroxy-N-[2-(1-hydroxy-4-oxocyclohexa-2,5-dien-1-yl)ethyl]octahydroisoindole-1-one* (**15**). Compound **15** was prepared from **12** as a beige solid and purified by flash chromatography using EtOAc. Yield: 65%; *R*_f_ = 0.14 (EtOAc); m.p.: 127–128 °C; ^1^H-NMR (400 MHz, DMSO-*d*_6_) δ 6.97–6.91 (m, 2H, H-2/6), 6.08 (d, *J* = 11.0 Hz, 2H, H-3/5), 5.90 (d, *J* = 6.6 Hz, 1H, 9′-OH), 5.82 (s, 1H, 1-OH), 4.55 (d, *J* = 6.6 Hz, 1H, H-9′), 3.39–3.31 (m, 1H, H-8_(a)_), 3.05–2.96 (m, 1H, H-8_(b)_), 2.63–2.56 (m, 1H, H-3′), 2.06–1.98 (m, 1H, H-8′), 1.89–1.82 (m, 1H, H-7_(a)_), 1.83–1.77 (m, 1H, H-4′_(a)_), 1.80–1.72 (m, 1H, H-7_(b)_), 1.72–1.66 (m, 1H, H-7′_(a)_), 1.49–1.35 (m, 3H, H-4′_(b)_/5′_(a)_/6′_(a)_), 1.19–1.06 (m, 1H, H-6′_(b)_), 0.95–0.89 (m, 1H, H-5′_(b)_), 0.91–0.83 (m, 1H, H-7′_(b)_) ppm; ^13^C-NMR (100 MHz, DMSO-*d*_6_) δ 185.6 (C-4), 175.2 (C-2′), 153.2 (C-2/6), 127.3 (C-3/5), 85.6 (C-9′), 68.1 (C-1), 40.8 (C-8′), 38.5 (C-3′), 38.1 (C-7), 35.2 (C-8), 26.3 (C-7′), 23.3 (C-6′), 23.2 (C-4′), 23.1 (C-5′) ppm; HRMS (EI) calcd. for C_16_H_21_NO_4_ [M]^+^ = 291.1471; Found: 291.1469.

### 3.3. Cytotoxicity against Human (Cancer) Cells

#### 3.3.1. Cell Culture

Human CCRF-CEM leukemia and MDA-MB-231 breast cancer cells lines were kept in RPMI1640 medium (Gibco^®^, ThermoFisher Scientific Inc., New York, NY, USA), supplemented with 2 mM l-glutamine (Gibco^®^), 10% heat-inactivated foetal bovine serum (FBS, Gibco^®^), 100 units/mL Penicillin (PAA), and 100 µg/mL Streptomycin (Gibco^®^) (1% Pen/Strep). HCT-116 and U251 cells were cultured in high-glucose Dulbecco’s Modified Eagle Medium (DMEM, Gibco^®^) containing 2 mM l-glutamine, 10% FBS, and 1% Pen/Strep. MRC-5 cells were grown in Minimum Essential Medium (MEM, Gibco^®^) supplemented with 2 mM l-glutamine, 10% FBS, and 1% Pen/Strep. All cells were kept in a humidified 5% CO_2_ atmosphere at 37 °C and passaged at 90% confluence.

#### 3.3.2. XTT Viability Assay

A Cell Proliferation Kit II (XTT) was purchased from Sigma-Aldrich and performed as described previously [65] and in accordance with the manufacturer’s protocol. In brief, adherent cell lines were seeded at a density of 50,000 cells/mL or 100,000 cells/mL (MRC-5) in 96 well plates (100 µL, flat bottom) and grown for 24 h before test compounds were added. Suspension cells (CCRF-CEM) were seeded at 100,000 cells/mL and test compounds were added immediately. After 72 h, XTT solution was added for another 90 min or 4 h (CCRF-CEM cells) and absorbance was measured at 490 nm with a reference wave length of 650 nm (Hidex Sense Microplate Reader 425-301, Hidex, Turku, Finland). Results are expressed as a percentage of the vehicle-treated (0.5% DMSO) control cells. Vinblastine served as the positive control (0.01 µg/mL).

### 3.4. In Vitro Growth Inhibition Assay of Plasmodium Falciparum NF54

In vitro activity against erythrocytic stages of *P. falciparum* was determined by a modified [^3^H]-hypoxanthine incorporation assay [66] using the drug-sensitive NF54 strain and the standard drug, chloroquine (Sigma C6628). Briefly, parasite cultures incubated in RPMI 1640 medium with 5% AlbuMAX^TM^ (without hypoxanthine) were exposed to serial drug dilutions in microtiter plates. After 48 h of incubation at 37 °C in a reduced oxygen atmosphere, 0.5 μCi [^3^H]-hypoxanthine was added to each well of the plate. Cultures were incubated for a further 24 h before they were harvested onto glass-fiber filters and washed with distilled water. The radioactivity was counted using a Betaplate^TM^ liquid scintillation counter (Wallac, Zurich). The results were recorded as counts per minute (CPM) per well at each drug concentration and expressed as a percentage of the untreated controls. IC_50_ values were calculated from the sigmoidal inhibition curves using Microsoft Excel. Chloroquine was used as the control.

### 3.5. In vitro Growth Inhibition Assay of Trypanosoma Brucei Rhodesiense

*Trypanosoma brucei rhodesiense*, STIB 900 strain, and the standard drug, melarsoprol, were used for the assay. Minimum Essential Medium (50 μL) supplemented with 25 mM HEPES, 1g/L additional glucose, 1% MEM non-essential amino acids (100×), 0.2 mM 2-mercaptoethanol, 1 mM Na-pyruvate, and 15% heat-inactivated horse serum was added to each well of a 96-well microtiter plate [67]. Serial drug dilutions of 11 three-fold dilution steps covering a range from 100 to 0.002 μg/mL were prepared. Then, 4 × 10^3^ bloodstream forms of *T. b. rhodesiense* (STIB 900) in 50 μL were added to each well and the plate was incubated at 37 °C under a 5% CO_2_ atmosphere for 72 h. 10 μL Alamar Blue (resazurin, 12.5 mg in 100 mL double-distilled water) was then added to each well and incubation continued for a further 2–4 h [68]. Then, the plates were read with a Spectramax Gemini XS microplate fluorometer (Molecular Devices Cooperation, Sunnyvale, CA, USA) using an excitation wavelength of 536 nm and an emission wavelength of 588 nm. The IC_50_ values were calculated from the sigmoidal inhibition curves using the microplate reader software, Softmax Pro (Molecular Devices Cooperation, Sunnyvale, CA, USA). Melarsoprol was used as the control.

### 3.6. Cytotoxicity against L6 Cells

Assays were performed in 96-well microtiter plates, each well containing 100 μL of RPMI 1640 medium supplemented with 1% l-glutamine (200 mM) and 10% foetal bovine serum, and 4000 L6 cells (a primary cell line derived from rat skeletal myoblasts). Serial drug dilutions of 11 threefold dilution steps covering a range from 100 to 0.002 μg/mL were prepared. After 72 h of incubation, the plates were inspected under an inverted microscope to assure growth of the controls and sterile conditions. 10 μL of Alamar Blue solution was then added to each well and the plates incubated for another 2 h. Then, the plates were read with a Spectramax Gemini XS microplate fluorometer (Molecular Devices Cooperation, Sunnyvale, CA, USA) using an excitation wavelength of 536 nm and an emission wavelength of 588 nm. The IC_50_ values were calculated by linear regression from the sigmoidal dose inhibition curves using the microplate reader software, Softmax Pro (Molecular Devices Cooperation, Sunnyvale, CA, USA). Podophyllotoxin (Sigma P4405) was used as the control.

## 4. Conclusions

In conclusion, we have synthesized a series of jacaranone imides and amines with promising antiproliferative activities from commercially available methyl hydroxyphenyl acetate (**4**) and tyramine (**8**). Although the substances showed beneficial physicochemical properties, antiprotozoal effects remained comparatively weak. Imide **13i** showed the highest activity against *P. falciparum* NF54 with an IC_50_ of 1.28 µM, while the lowest IC_50_ against *T. b. rhodesiense* was displayed by the morpholine derivate **14c** with 0.27 µM. The conjugated imides, **13b**, **13e**, and **13i**, exhibited the overall highest cytotoxicity against all tested cancer cell lines.

We are aware that some of the performed chemical modifications did not result in druggable chemical entities. However, we consider that the exploration of a multifaceted chemical lead as present in the jacaranone scaffold through systematic derivatizations may open doors to unexpected biological properties.

## Figures and Tables

**Table 1:** In vitro antiparasitic activity, host toxicity, and key physicochemical properties of tested compounds.

Compd.	*P. falc.* ^a^	SI ^b^	*T.b.rhod.* ^c^	SI ^b^	Cyt. L6 ^d^	Chemical Structure	log*P*	tPSA (7.4)
	IC_50_ (μM)		IC_50_ (μM)		IC_50_ (μM)			
Chl.	0.002							
Mel.			0.004					
Pod.					0.007			
**13a**	3.78	1.3	1.58	3.2	5.08	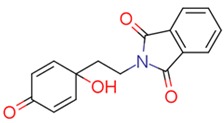	0.85	74.68
**13b**	4.42	6.1	2.15	12.5	26.80	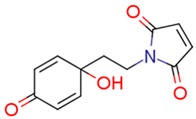	−0.18	74.68
**13c**	8.63	1.1	6.93	1.4	9.90	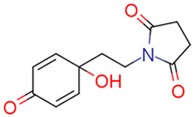	−0.60	74.68
**13d**	3.24	1.9	5.71	1.1	6.28	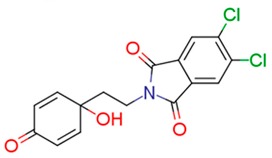	1.89	74.68
**13e**	74.38	0.1	2.26	4.5	10.26	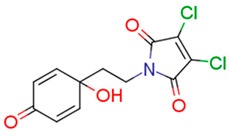	−0.38	74.68
**13f**	28.04	5.5	44.75	3.4	153.48	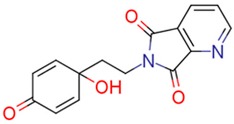	−0.06	87.57
**13g**	11.82	6.6	15.32	5.1	77.54	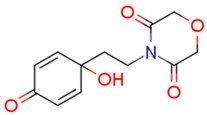	−1.13	83.91
**13h**	3.04	1.0	1.70	1.7	2.96	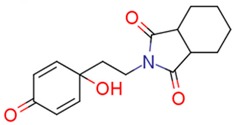	0.81	74.68
**13i**	1.28	2.7	0.80	4.4	3.52	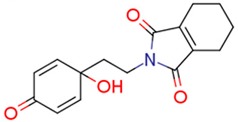	0.66	74.68
**13j**	3.93	2.0	0.97	8.3	8.04	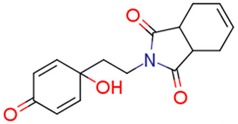	0.55	74.68
**14a**	2.91	1.0	0.57	4.9	2.81	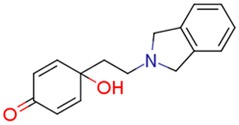	1.89	41.74
**14c**	10.93	0.3	0.27	12.9	3.46	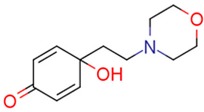	0.04	49.77
**15**	6.18	2.0	1.52	8.3	12.63	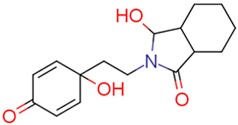	0.58	77.84

^a^*P. falciparum*, strain NF54, erythrocytic stages; ^b^ SI is the ratio: IC_50_ in L6 cells/IC_50_ in each parasite; ^c^ *T. brucei rhodesiense*, strain STIB900 trypomastigote forms; ^d^ cytotoxicity L6 cells rat skeletal myoblasts. Reference drugs: Chloroquine (chl., IC_50_ = 0.002 ± 0.001), melarsoprol (mel., IC_50_ = 0.004 ± 0.003), podophyllotoxin (pod., IC_50_ = 0.007 ± 0.002). The IC_50_ value of each reference drug is the mean from multiple measurements taken in parallel with the compounds of interest. IC_50_ values of the tested compounds are means of two to three independent assays. The individual IC_50_ values within each assay varied <25%. The physical properties were predicted by using Marvin 18.10.0, ChemAxon (https://www.chemaxon.com).

## Supplementary Materials

Supplementary material associated with this article are available online. Contents: Compared overall yields of the key products. Calculated physicochemical parameters and models. Full results of the XTT viability assay. Data and NMR spectra of the prepared compounds.
