# Supplementary material for: Synthesis of Jacaranone-Derived Nitrogenous Cyclohexadienones and Their Antiproliferative and Antiprotozoal Activities
Source: Molecules. 2018 Nov 7;23(11):2902. doi: 10.3390/molecules23112902 (PMC6278284; doi:10.3390/molecules23112902)

Synthesis of Jacaranone-derived Nitrogenous Cyclohexadienones and Their Antiproliferative and Antiprotozoal Activities

Armin Presser[1] ● Gunda Lainer[1] ● Nadine Kretschmer[2] ● Wolfgang Schuehly[2] ● Robert Saf[3]● Marcel Kaiser[4,5] ● Marc-Manuel Kalt[1]

1 Institute of Pharmaceutical Sciences, Pharmaceutical Chemistry, University of Graz, Schubertstraße 1, A-8010 Graz, Austria

2 Institute of Pharmaceutical Sciences, Pharmacognosy, University of Graz, Universitätsplatz 4, A-8010 Graz, Austria

3 Institute for Chemistry and Technology of Materials (ICTM), Graz University of Technology, Stremayrgasse 9, A-8010 Graz, Austria

4 Swiss Tropical and Public Health Institute, Socinstrasse 57, CH-4002 Basel, Switzerland

5 University of Basel, Petersplatz 1, 4003 Basel, Switzerland

Supporting Information

Table of contents

1. Compared overall yields of the key products S2-S4

2. Calculated physicochemical parameters and models S5-S9

3. Results of the XTT viability assay S10-11

3. Data and NMR spectra of the prepared compounds S12-S45

Table 1: Synthesis and compared overall yields of compounds **7**, **11** and **12**, final oxidation step to the dienones **13 – 15**

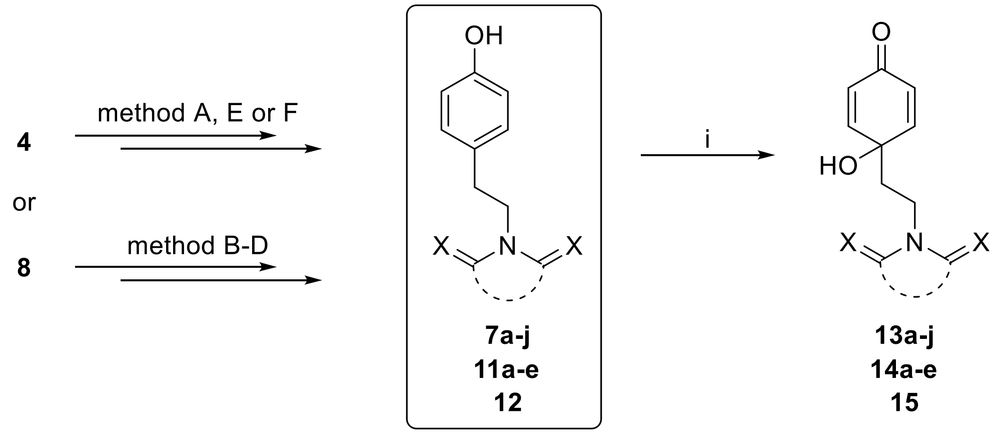

Entry	X	Method[a]	Product	Yield/%[b]
1	O	A	**7a**	71
2	O	B	**7a**	73
3	O	C	**7a**	23
4	O	A	**7b**	58
5	O	B	**7b**	55
6	O	C	**7b**	51
7	O	A	**7c**	62
8	O	B	**7c**	58
9	O	C	**7c**	98
10	O	B	**7d**	89
11	O	C	**7d**	67
12	O	B	**7e**	90
13	O	C	**7e**	67
14	O	B	**7f**	0

15	O	C	**7f**	86
16	O	B	**7g**	0
17	O	C	**7g**	71
18	O	B	**7h**	86
19	O	C	**7h**	79
20	O	B	**7i**	92
21	O	C	**7i**	98
22	O	B	**7j**	79
23	O	C	**7j**	98
24	H, H	B→D	**11a**	53
25	H, H	F	**11a**	48
27	H, H	D	**11b**	0
28	H, H	E	**11b**	42
29	H, H	F	**11b**	64
30	H, H	D	**11c**	0
31	H, H	F	**11c**	69
32	H, H	D	**11d**	0
33	H, H	F	**11d**	61
34	H, H	B→D	**11e**	53
35	O, OH	B→D	**12**	76

Reaction conditions: (*i*) **13a-j**, **15**: PhI(OAc)$_2$, CH_3CN/H_2O (12:5), 0 °C, 7 min (**13a**: 67%, **13b**: 17%, **13c**: 55%, **13d**: 40%, **13e**: 64%, **13f**: 19%, **13g**: 18%, **13h**: 79%, **13i**: 88%, **13j**: 49%, **15**: 65%); **14a-e**: PhI(OAc)$_2$,

CH_3CN/H_2O/phosphate buffer (12:3:2), pH = 6.4, 0 °C, 7 min (**14a**: 16%, **14b**: 0%, **14c**: 28%, **14d**: 0%, **14e**: 0%).

[a]Method A: preparation of imides via Mitsunobu reaction; method B: AcOH-assisted condensation of tyramine; method C: PEG 400-assisted condensation of tyramine; method D: preparation of amines from imides; method E: preparation of amines by catalytic amination; method F: preparation of amines via alkyl bromides

[b]Isolated yield.

Table 2: Calculated physicochemical properties of the tested compounds.

compd	MW	log*P*	log*S*	HBD	HBA	tPSA (A^2)	ASA (A^2)	ASA*pho* (A^2)	ASA*pol* (A^2)
						pH 7.4	pH 7.4	pH 7.4	pH 7.4
**13a**	283.28	0.85	-4.38	1	4	74.68	398.81	297.48	101.33
**13b**	233.22	-0.18	-3.11	1	4	74.68	309.98	200.20	109.77
**13c**	235.24	-0.60	-2.09	1	4	74.68	347.12	241.44	105.68
**13d**	352.17	1.89	-5.79	1	4	74.68	433.57	332.23	101.34
**13e**	302.11	-0.38	-4.12	1	4	74.68	348.25	231.55	116.70
**13f**	284.27	-0.06	-1.76	1	5	87.57	391.80	273.57	118.23
**13g**	251.24	-1.13	-2.34	1	5	83.91	361.20	242.04	119.16
**13h**	289.33	0.81	-3.86	1	4	74.68	395.92	302.04	93.88
**13i**	287.32	0.66	-4.34	1	4	74.68	400.77	305.51	95.26
**13j**	287.32	0.55	-3.15	1	4	74.68	371.43	276.99	94.44
**14a**	255.32	1.89	0.00	1	3	41.74	401.42	337.80	63.62
**14c**	223.27	0.04	0.58	1	4	49.77	363.97	285.49	78.49
**15**	291.35	0.58	-3.83	2	4	77.84	397.62	303.73	93.89

The molecular weight (MW), log*P*, log*S*, hydrogen bond donor (HBD), hydrogen bond acceptor (HBA), topological polar surface area (tPSA), accessible surface area (ASA), hydrophobic accessible surface area (ASA*pho*) and polar accessible surface area (ASA*pol*) were calculated using Marvin 18.10.0, ChemAxon (https://www.chemaxon.com).

Table 3: Calculated ligand efficiency metrics of the tested compounds.

compd	*P. falciparum*			*T. brucei rhodesiense*		
	LE	LLE	LELP	LE	LLE	LELP
**13a**	0.35426	4.3071	3.1496	0.37897	4.6853	2.9443
**13b**	0.43214	5.9852	-1.4583	0.4574	6.2982	-1.3778
**13c**	0.40866	5.4188	-0.86821	0.41635	5.5141	-0.85216
**13d**	0.32745	3.1621	7.1088	0.31276	2.9158	7.4427
**13e**	0.2981	3.9202	0.6991	0.4076	5.4367	0.51128
**13f**	0.29739	4.3834	0.56794	0.28413	4.1803	0.59445
**13g**	0.37554	6.1041	-3.1336	0.36695	5.9914	-3.207
**13h**	0.36037	4.9418	1.5945	0.37696	5.1957	1.5243
**13i**	0.38487	5.2504	1.6655	0.3984	5.4575	1.6089
**13j**	0.35311	5.1061	0.84732	0.3928	5.7136	0.76171
**14a**	0.39973	4.7611	1.9388	0.45074	5.4676	1.7194
**14c**	0.42541	5.0059	-0.10437	0.56335	6.6146	-0.078814
**15**	0.3403	4.4189	2.3224	0.38014	5.0287	2.079

The ligand efficiency (LE), lipophilic ligand efficiency (LLE) and ligand efficiency lipophilic price (LELP) are based on IC_{50} values in nmol/L and were calculated using the DataWarrior software, version 4.7.2 (http://www.openmolecules.org/datawarrior.html).

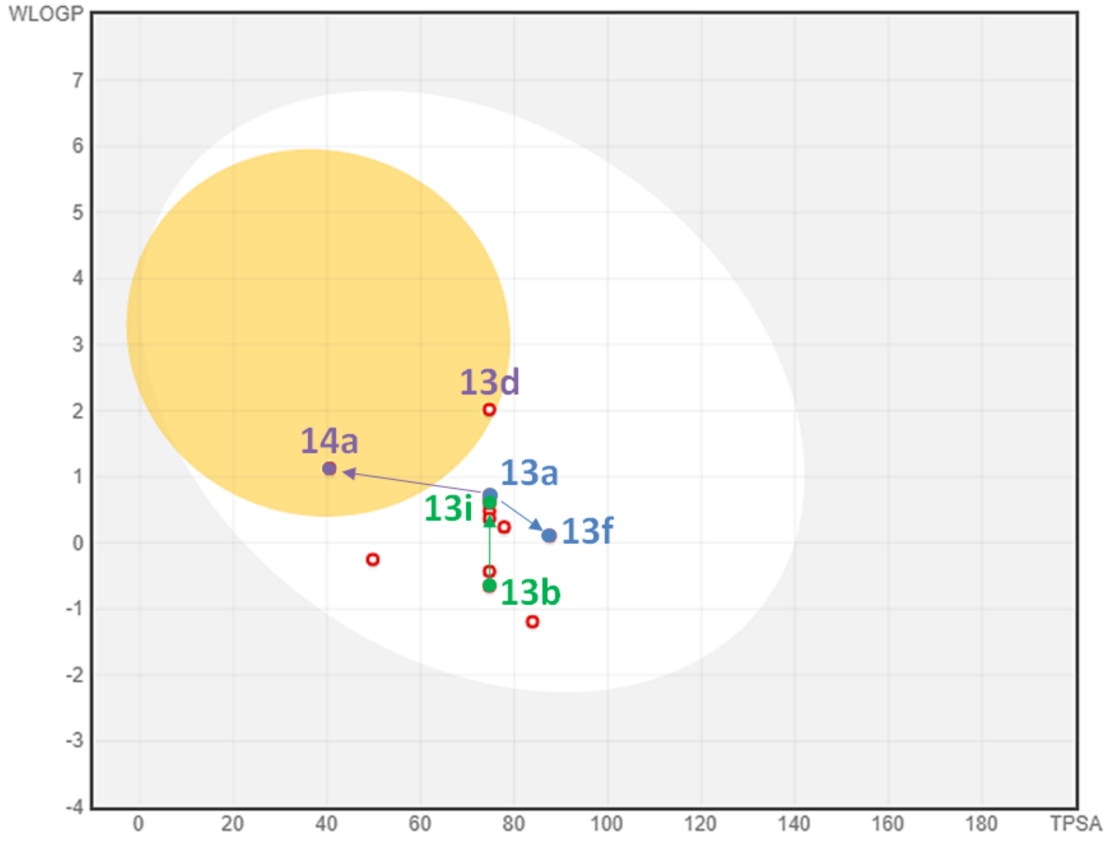

Fig. 1 BOILED-Egg analysis of all synthesized dienones. Substances within the white ellipse (egg white) are anticipated to have good intestinal absorption (passive absorption); the yellow region (yolk) is the physicochemical space of molecules with high probability to permeate the blood-brain barrier. The BOILED–Egg model also reflects the variability in IC_{50} values of our evaluated compounds after altering the dienone skeleton. The plot was prepared by using the free web tool SwissADME (www.swissadme.ch).

Table 4: Results of the XTT viability assay.

compd	CCRF-CEM		MDA-MB-231		HCT 116		U251		MRC-5	
	5 μg/mL	50 μg/mL	5 μg/mL	50 μg/mL	5 μg/mL	50 μg/mL	5 μg/mL	50 μg/mL	5 μg/mL	50 μg/mL
**13a**	4.42 ± 1.95	1.15 ± 0.57	37.76 ± 3.13	17.38 ± 1.50	55.36 ± 4.79	1.76 ± 0.15	90.65 ± 4.53	3.21 ± 0.65	43.74 ± 4.17	1.93 ± 0.27
**13b**	-1.08 ± 0.41	3.46 ± 0.46	1.71 ± 0.23	6.61 ± 0.28	0.13 ± 0.22	-0.32 ± 0.14	95.21 ± 2.89	-0.41 ± 0.13	14.61 ± 3.58	0.62 ± 0.05
**13c**	79.06 ± 7.04	2.15 ± 0.08	79.79 ± 4.02	5.72 ± 0.95	99.04 ± 2.69	1.10 ± 0.14	93.33 ± 4.65	0.59 ± 0.06	139.35 ± 3.57	3.55 ± 0.63
**13d**	13.80 ± 3.18	3.33 ± 0.82	28.59 ± 1.24	24.84 ± 2.30	41.24 ± 4.07	0.44 ± 0.13	72.11 ± 9.10	1.79 ± 0.51	46.44 ± 5.26	1.01 ± 0.14
**13e**	-2.60 ± 0.50	0.59 ± 0.16	81.32 ± 1.60	9.53 ± 0.37	45.33 ± 5.40	-0.43 ± 0.12	95.64 ± 2.66	1.95 ± 0.25	97.01 ± 5.81	0.38 ± 0.05
**13f**	99.83 ± 2.36	21.74 ± 6.20	112.14 ± 5.31	23.02 ± 1.33	90.99 ± 5.01	92.81 ± 7.25	91.76 ± 3.22	93.66 ± 2.82	108.57 ± 2.95	111.87 ± 2.68
**13g**	86.92 ± 6.14	8.05 ± 0.88	104.42 ± 2.43	7.63 ± 0.24	96.00 ± 3.74	68.10 ± 5.16	94.70 ± 1.74	65.60 ± 2.29	115.85 ± 3.68	89.59 ± 1.80

**13h**	15.70 ± 3.62	0.69 ± 0.47	70.84 ± 2.20	16.36 ± 0.39	44.30 ± 1.15	0.49 ± 0.19	74.92 ± 1.64	0.18 ± 0.07	57.62 ± 1.85	0.76 ± 0.07
**13i**	2.26 ± 0.96	1.23 ± 0.31	32.44 ± 2.46	16.56 ± 1.75	1.04 ± 0.50	0.15 ± 0.16	15.92 ± 4.35	0.00 ± 0.06	5.69 ± 0.18	0.51 ± 0.06
**13j**	89.92 ± 6.44	15.39 ± 1.27	87.73 ± 2.33	89.02 ± 2.76	98.62 ± 1.23	63.27 ± 1.00	96.54 ± 1.54	76.65 ± 1.92	96.08 ± 1.24	50.27 ± 4.44
**14a**	94.58 ± 7.31	57.97 ± 6.28	95.02 ± 4.23	92.47 ± 4.39	97.28 ± 2.36	62.12 ± 1.80	99.49 ± 0.50	95.54 ± 2.33	119.49 ± 4.68	95.12 ± 2.52
**14c**	22.81 ± 2.79	-0.18 ± 0.21	56.75 ± 2.79	4.33 ± 0.17	87.72 ± 3.25	1.58 ± 0.21	68.30 ± 4.96	1.11 ± 0.23	141.03 ± 4.17	0.79 ± 0.07
**15**	42.39 ± 2.45	0.57 ± 0.14	105.06 ± 7.90	6.05 ± 0.29	111.43 ± 6.45	1.44 ± 0.31	97.58 ± 3.54	2.24 ± 0.14	134.88 ± 2.14	4.47 ± 0.18

VBN	CCRF-CEM	MDA-MB-231	HCT 116	U251	MRC-5
(0.01 µg/mL)	23.60 ± 7.62	42.05 ± 7.97	38.99 ± 5.10	45.31 ± 3.81	63.82 ± 5.29

The XTT viability assay included leukemia (CCRF-CEM), breast cancer (MDA-MB-231), colon cancer (HCT-116) and glioblastoma cells (U251) as well as non-tumorigenic lung fibroblasts (MRC-5), the results are expressed as metabolic active cells in % of control, vinblastine (VBN) was used as reference compound.

2-[4-(Thexyldimethylsilyloxy)phenyl]ethanol (5): Colourless oil, 98%, R_f = 0.27 (CH:EtOAC = 2:1); ^{1}H NMR (400 MHz, $CDCl_3$): δ = 7.08 (d, *J* = 8.5 Hz, 2H, H-2/6), 6.78 (d, *J* = 8.5 Hz, 2H, H-3/5), 3.82 (t, *J* = 6.5 Hz, 2H, H-8), 2.80 (t, *J* = 6.5 Hz, 2H, H-7), 1.73 (hept, *J* = 6.9 Hz, 1H, CH-$(CH_3)_2$), 0.94 (d, *J* = 6.9 Hz, 6H, $(CH_3)_2$-CH), 0.94 (s, 6H, $(CH_3)_2$-C), 0.21 (s, 6H, $(CH_3)_2$-Si) ppm; ^{13}C NMR (100 MHz, $CDCl_3$): δ = 154.1 (C-4), 130.8 (C-1), 129.9 (C-2/6), 120.2 (C-3/5), 63.8 (C-8), 38.4 (C-7), 34.1 (CH-$(CH_3)_2$), 25.0 (C-$(CH_3)_2$), 20.1 ($(CH_3)_2$-C), 18.6 ($(CH_3)_2$-CH), -2.5 ($(CH_3)_2$-Si) ppm; HRMS (ESI) calcd. for $C_{16}H_{29}O_2Si$ ($[M+H]^+$): 281.1937; Found: 281.1931.

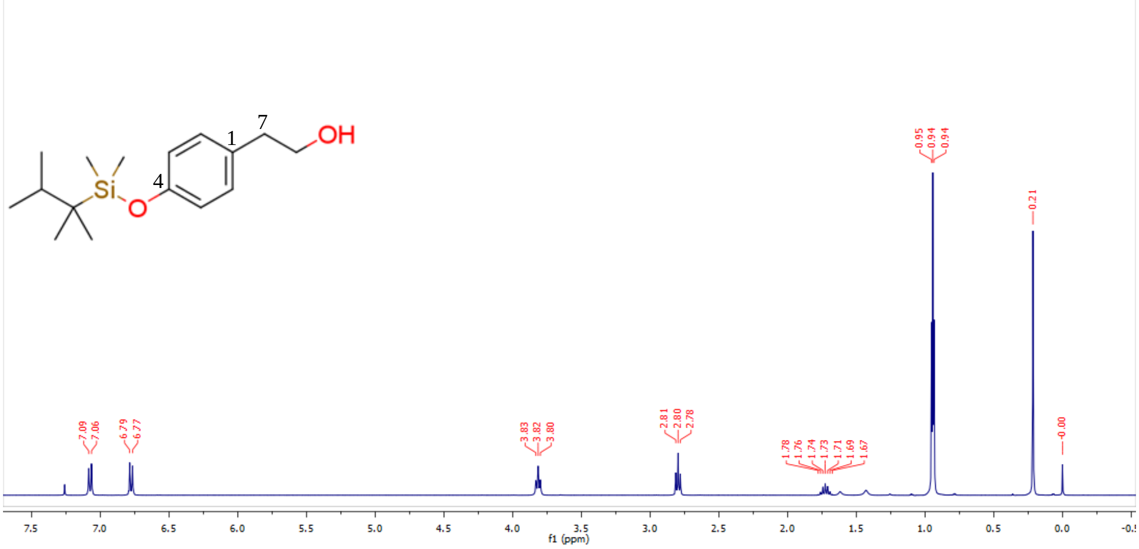

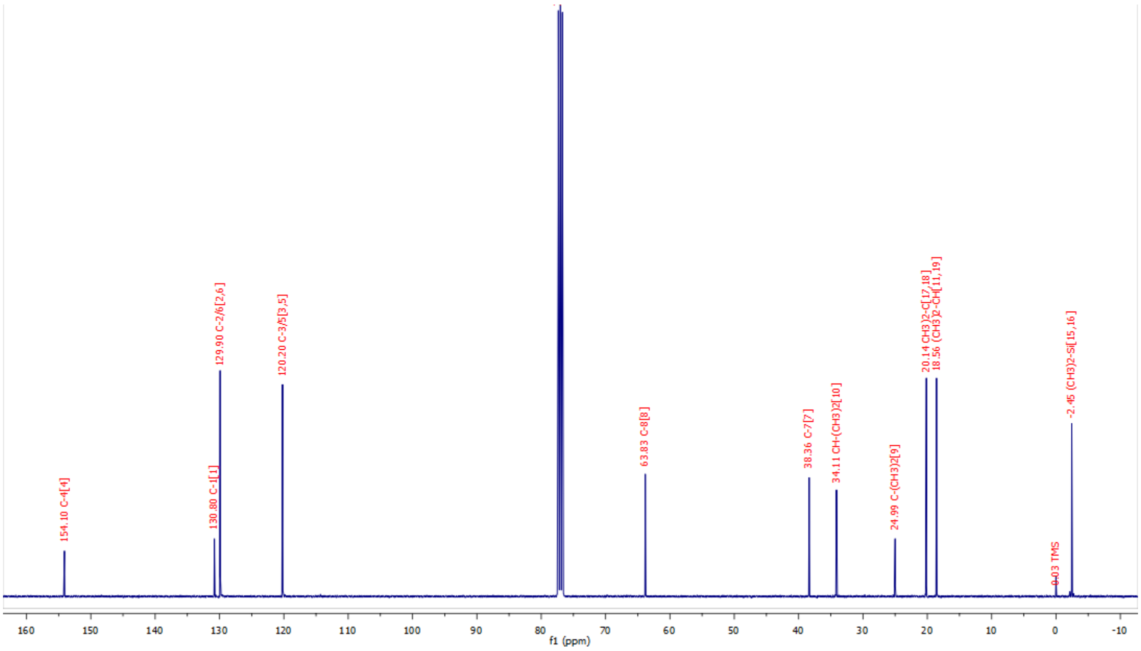

N-[4-(Thexyldimethylsilyloxy)phenethyl]phthalimide **(6a)**

Yellowish solid; Yield 90%; R_f = 0.26 (CH:EtOAC = 7:1).

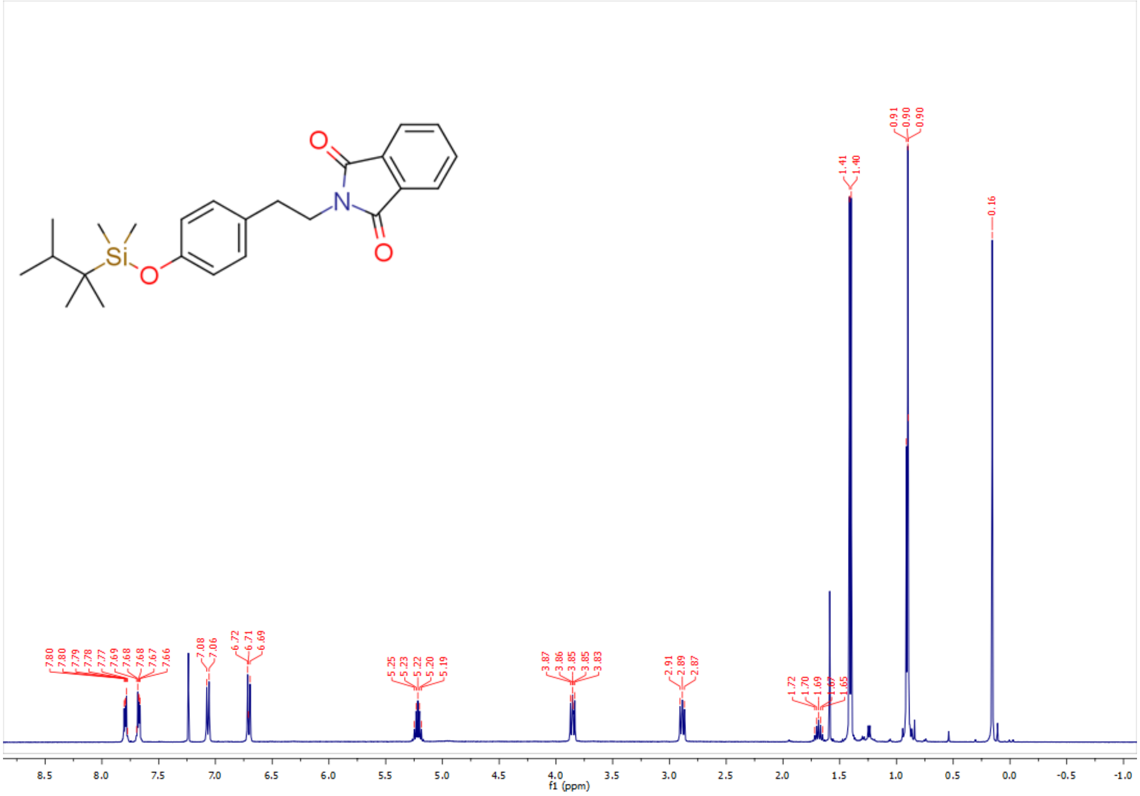

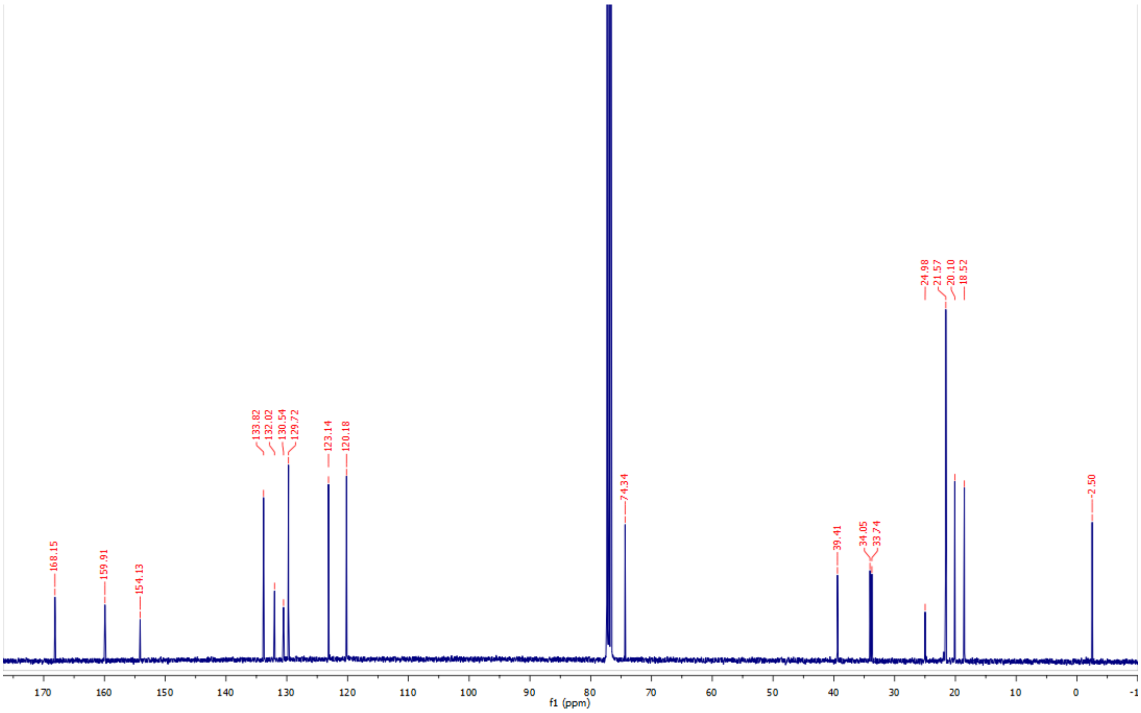

N-[4-(Thexyldimethylsilyloxy)phenethyl]maleimide **(6b)**

Yellowish solid; Yield 70%; R_f = 0.64 ($CHCl_3$:EtOAC = 9:1).

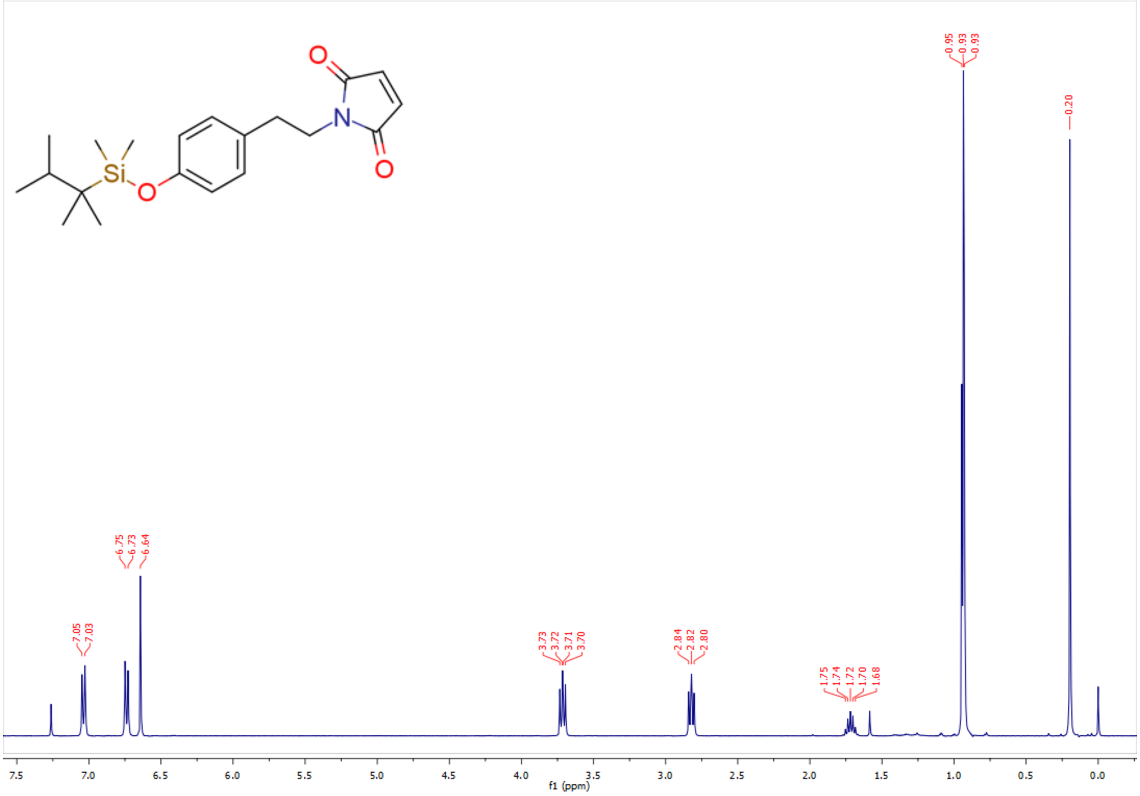

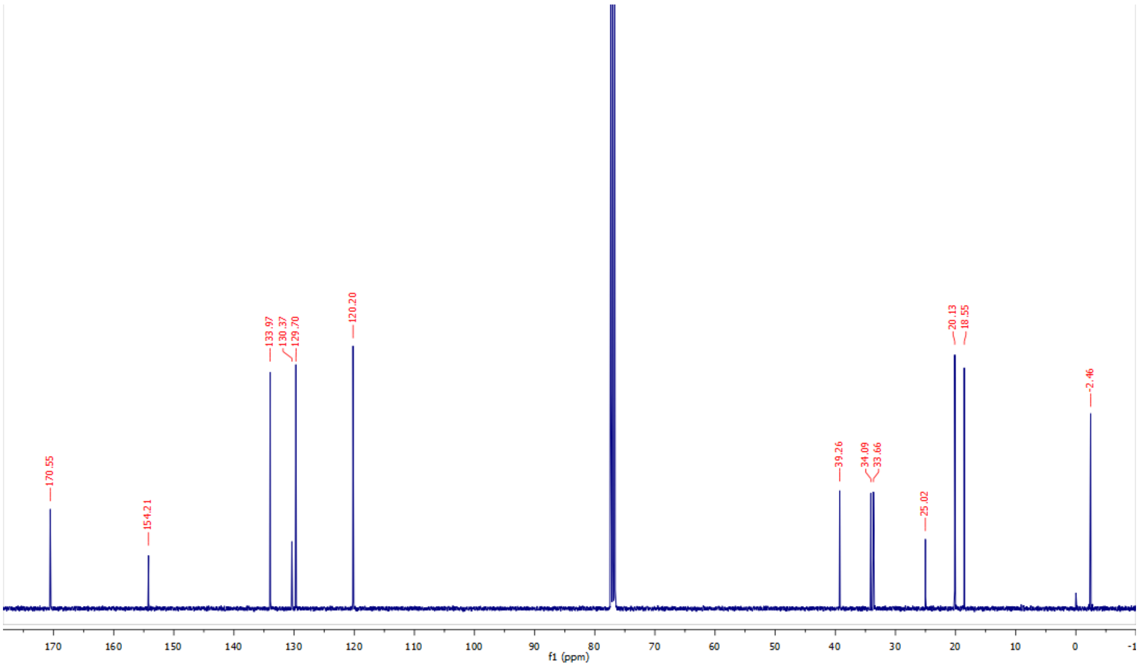

N-[4-(Thexyldimethylsilyloxy)phenethyl]succinimide **(6c)**

White solid; Yield 77%; R_f = 0.43 ($CHCl_3$:EtOAC = 9:1).

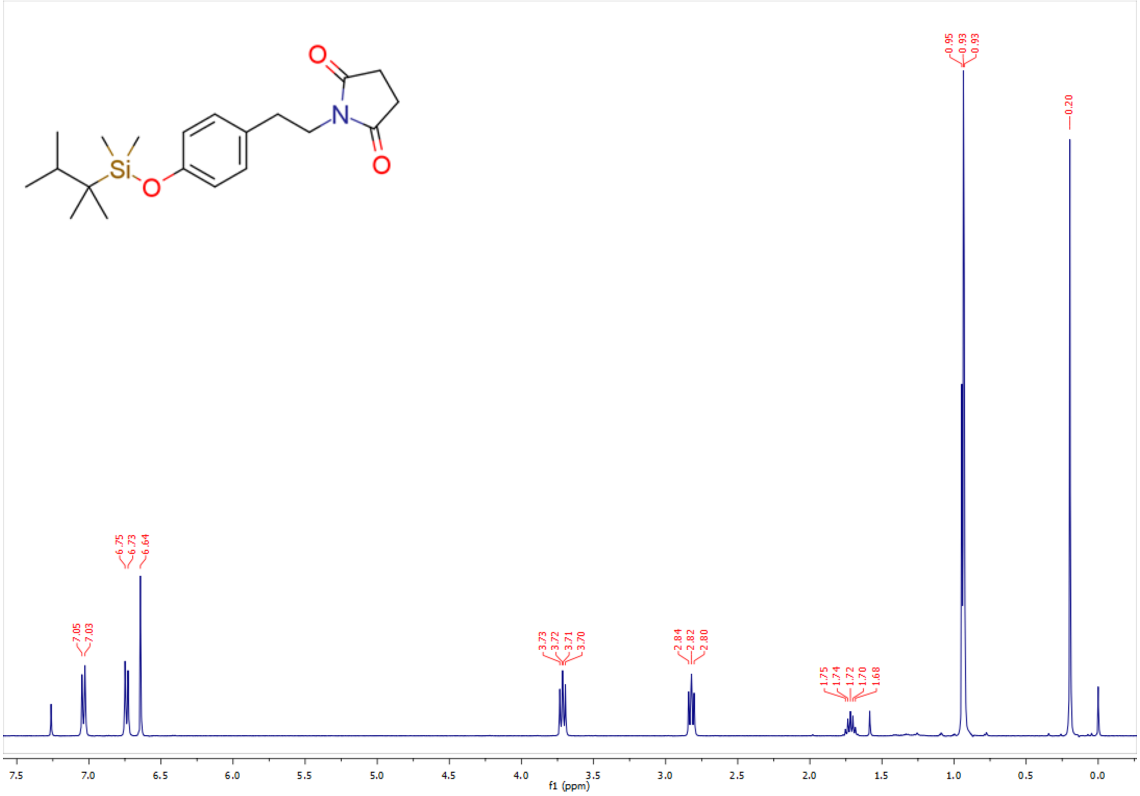

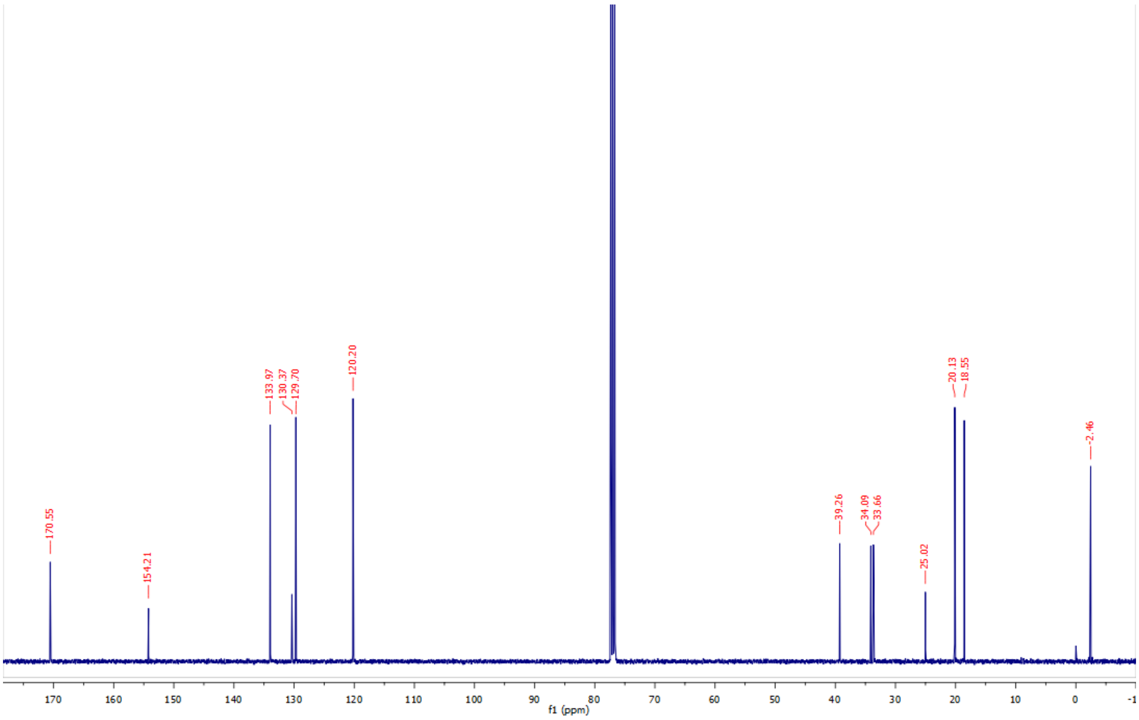

N-(4-Hydroxyphenethyl)phthalimide **(7a)**

White solid; Yield 73% (AcOH), 23% (PEG 400); R_f = 0.44 (CH:EtOAC = 1:1).

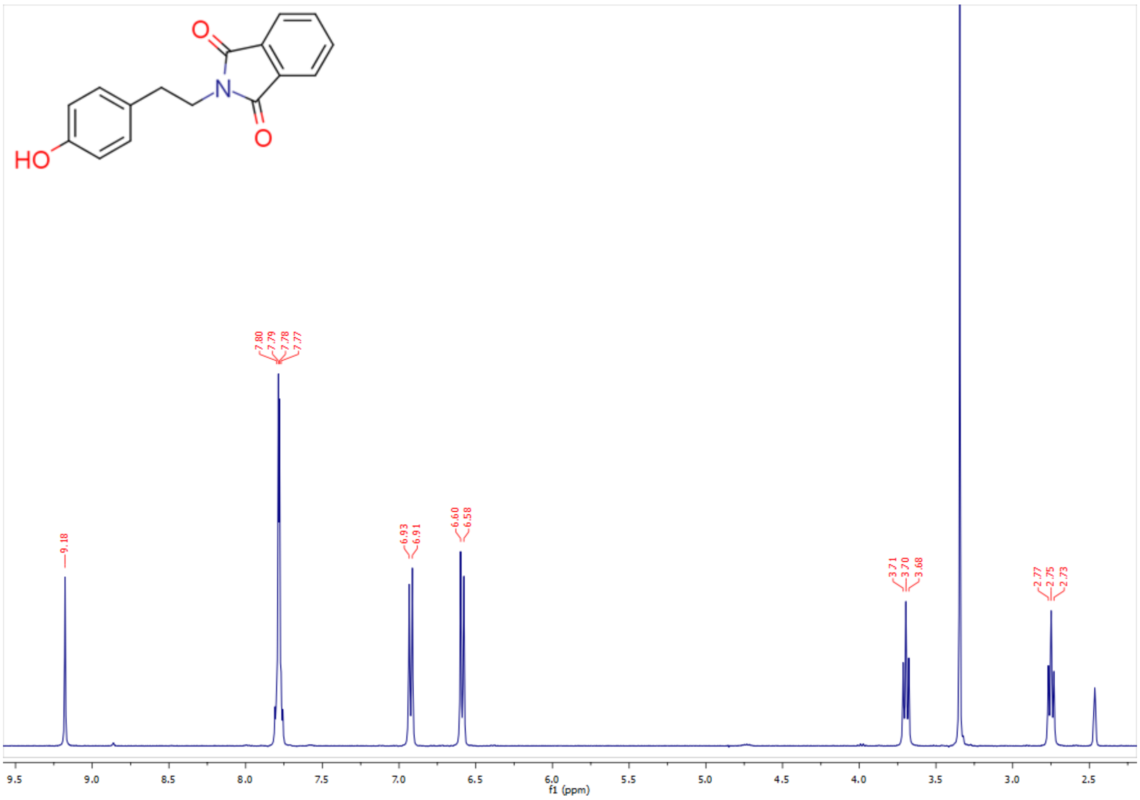

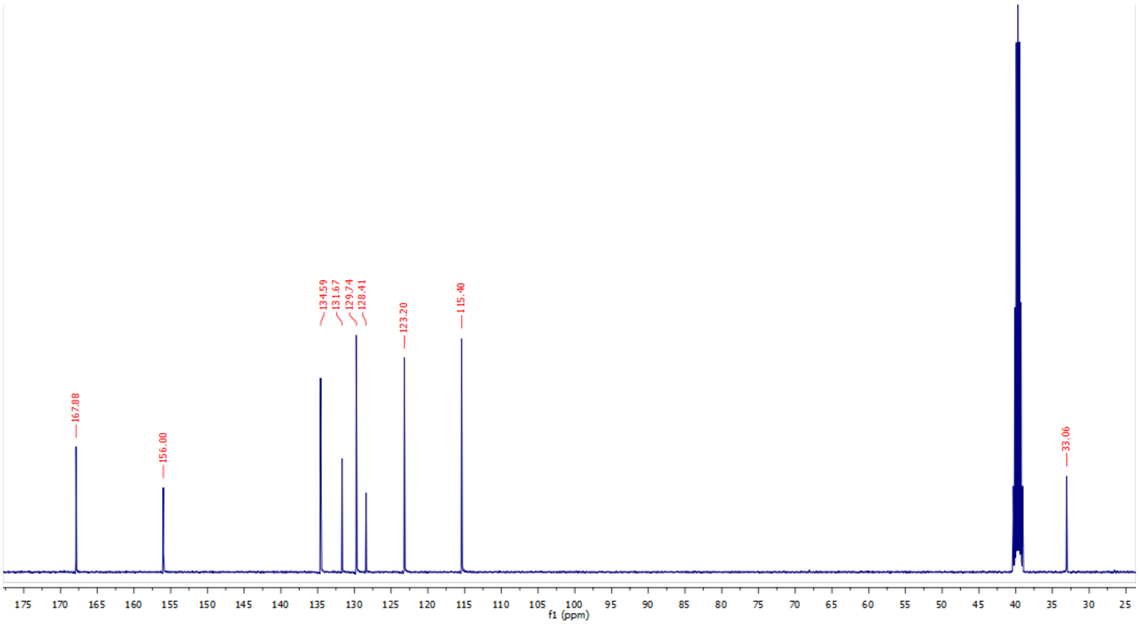

N-(4-Hydroxyphenethyl)maleimide **(7b)**

Slightly yellow solid; Yield 55% (AcOH), 51% (PEG 400); R_f = 0.38 (CH:EtOAC = 1:1).

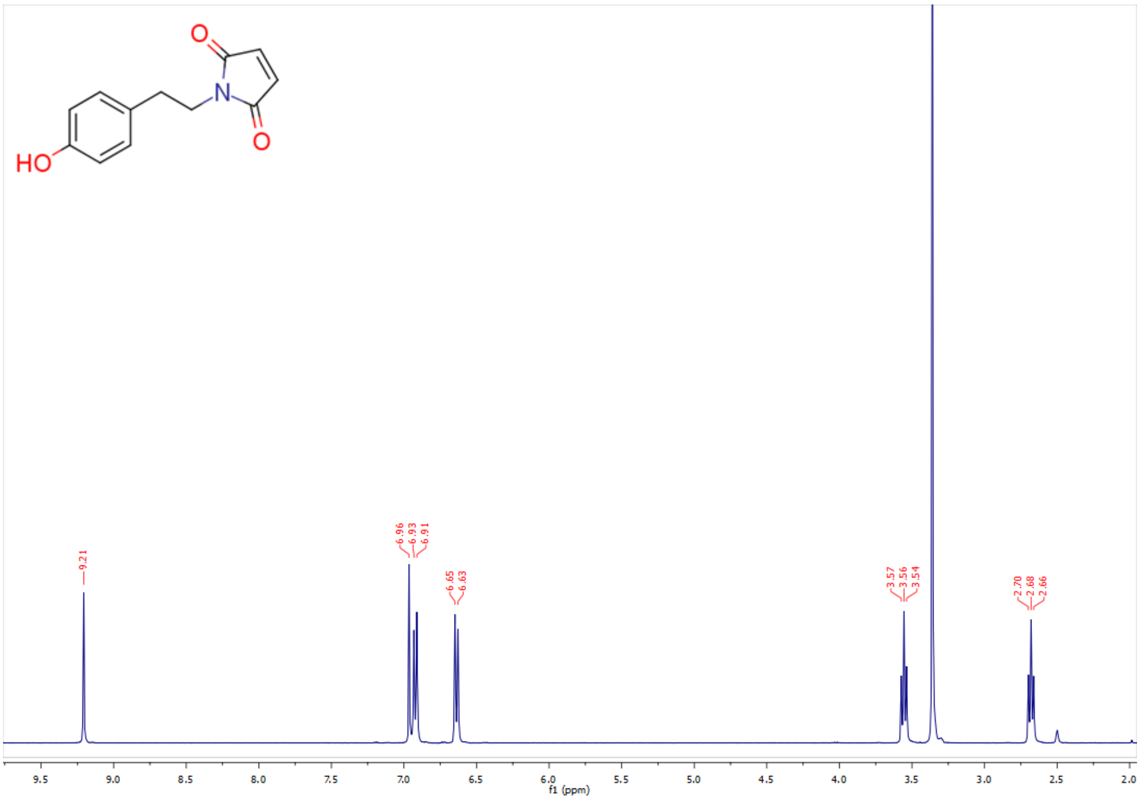

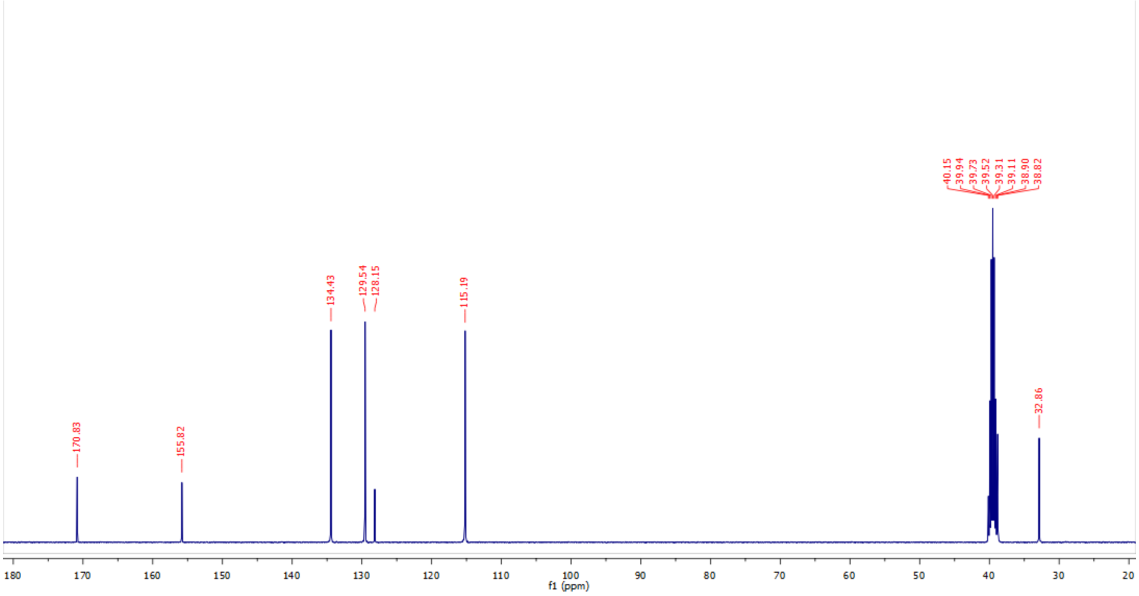

N-(4-Hydroxyphenethyl)succinimide **(7c)**

White solid; Yield 58% (AcOH), 98% (PEG 400); R_f = 0.17 (CH:EtOAC = 1:1).

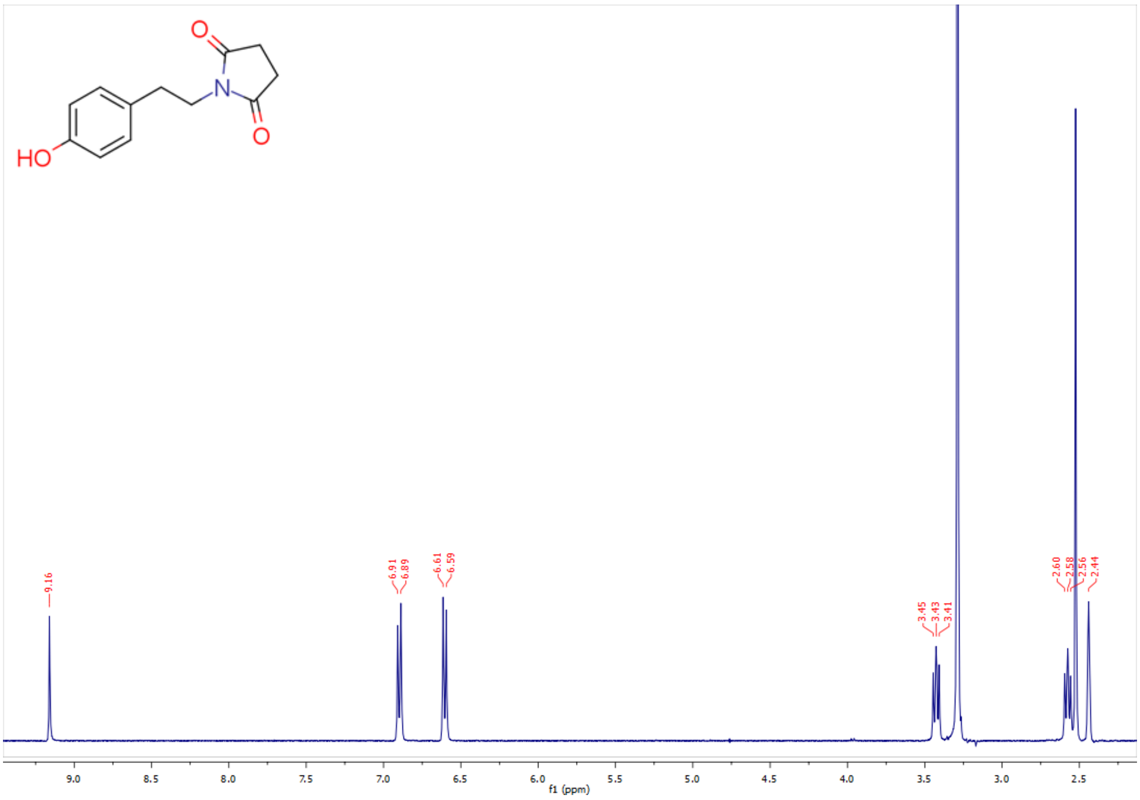

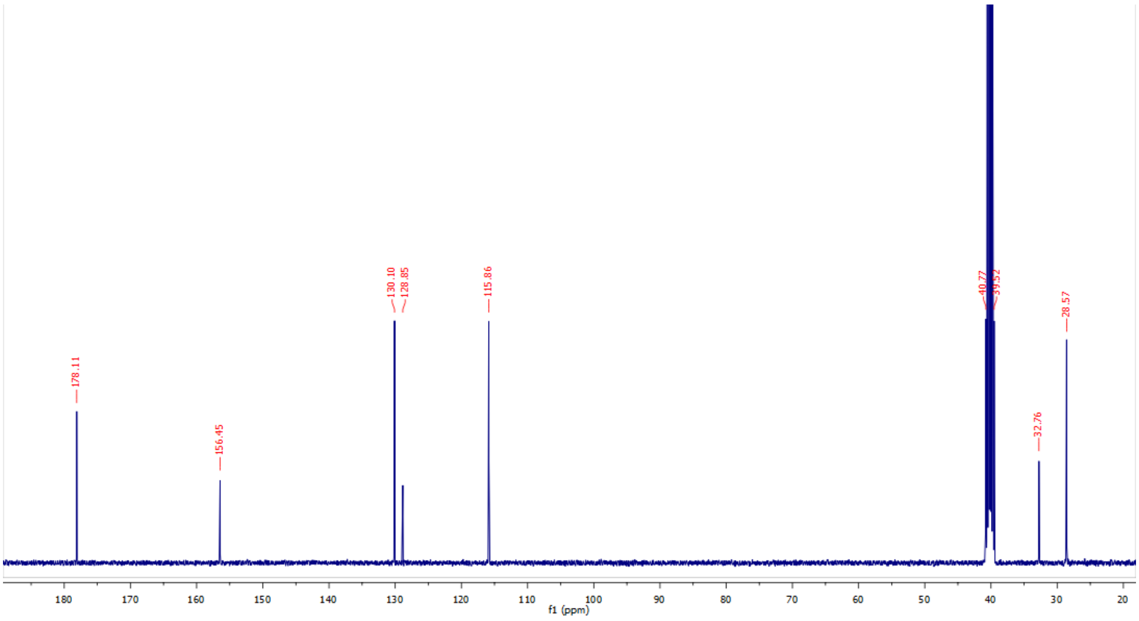

4,5-Dichloro-N-(4-hydroxyphenethyl)phthalimide **(7d)**

White solid; Yield 89% (AcOH), 67% (PEG 400); R_f = 0.52 (CH:EtOAC = 1:1).

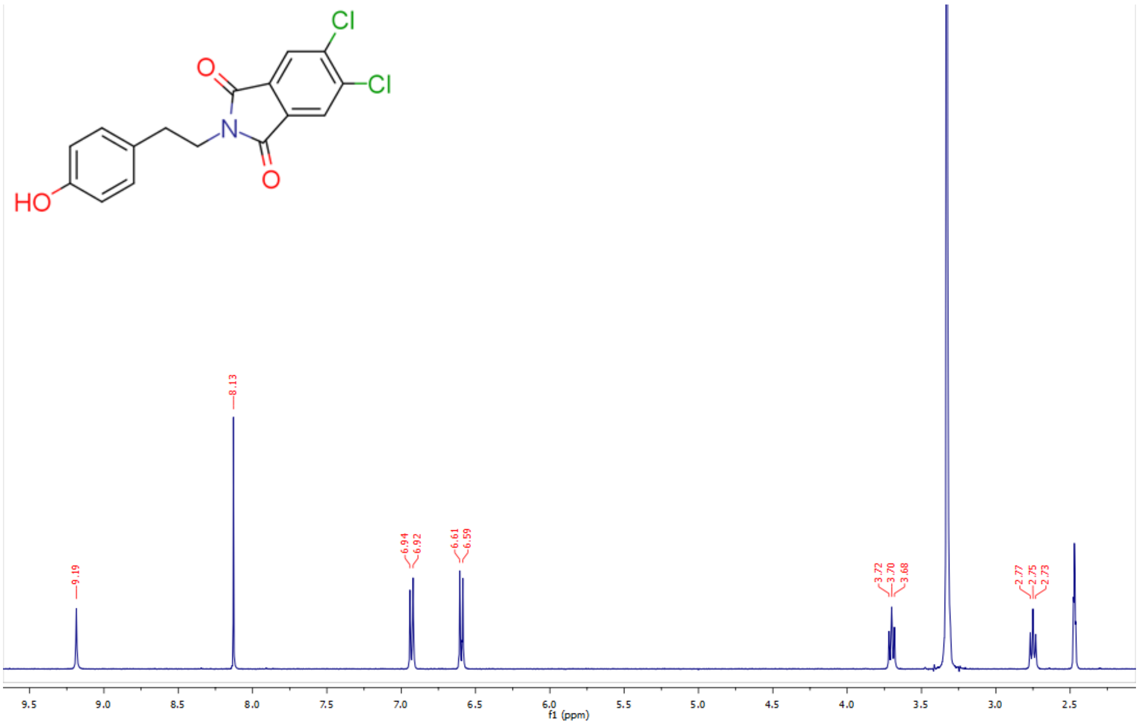

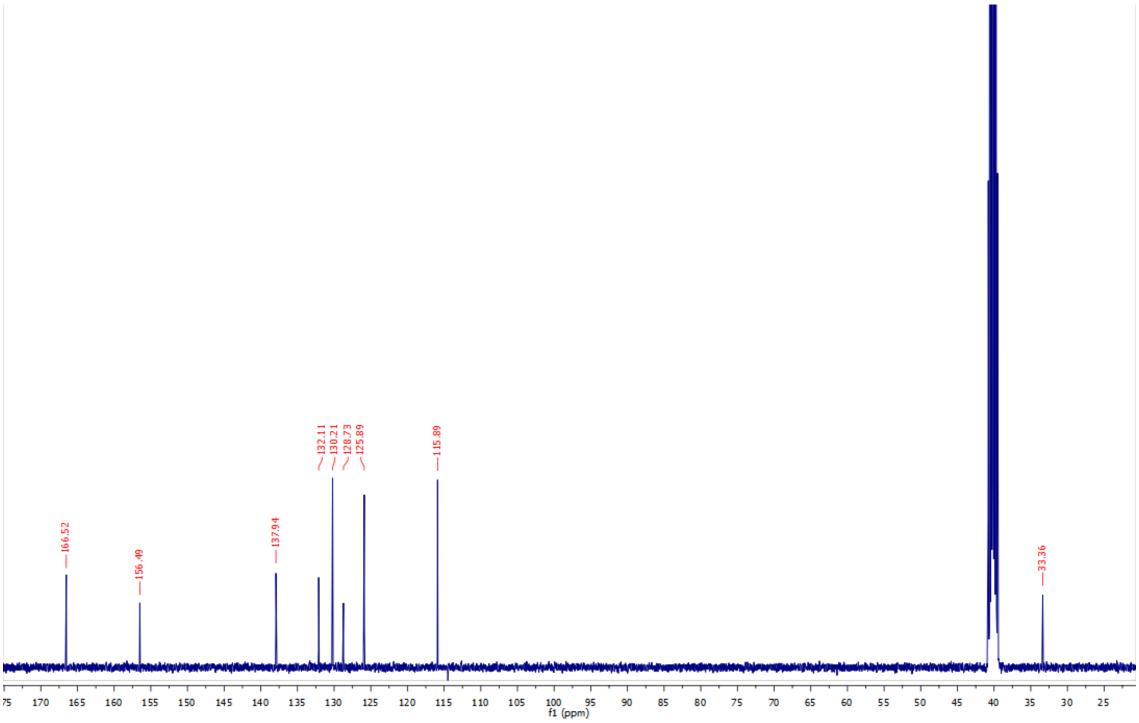

3,4-Dichloro-N-(4-hydroxyphenethyl)maleimide **(7e)**

White solid; Yield 90% (AcOH), 67% (PEG 400); R_f = 0.47 (CH:EtOAC = 1:1).

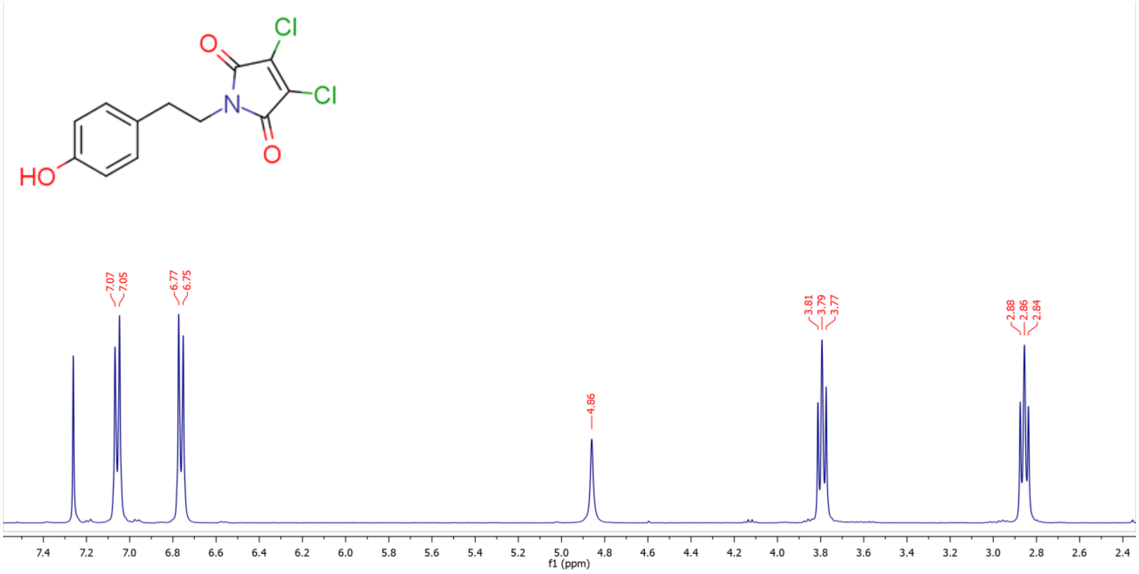

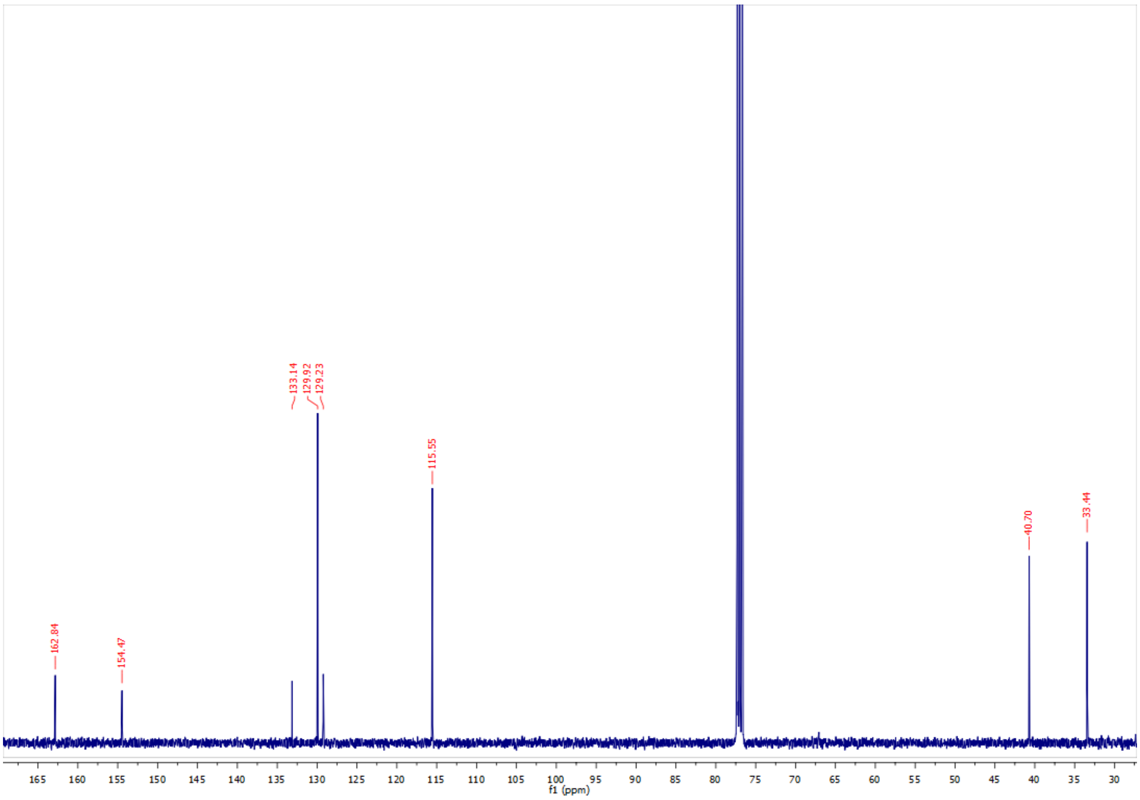

N-(4-Hydroxyphenethyl)pyridine-2,3-dicarboximide **(7f)**

White solid; Yield 0% (AcOH), 86% (PEG 400); R_f = 0.40 (CH:EtOAC = 1:1).

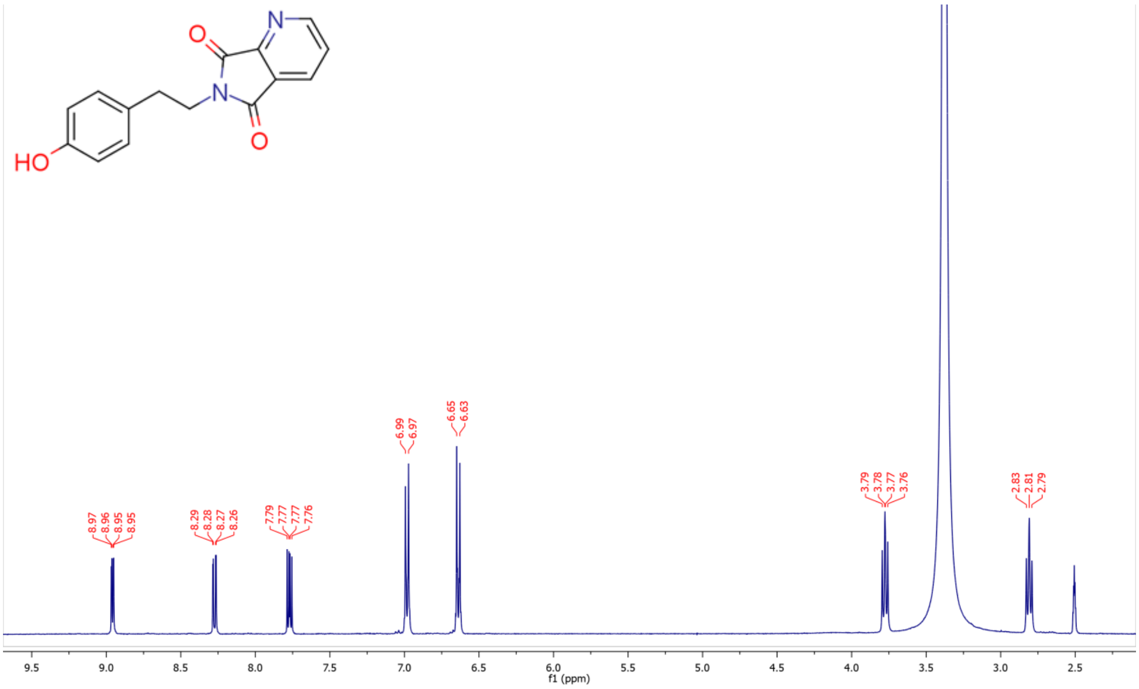

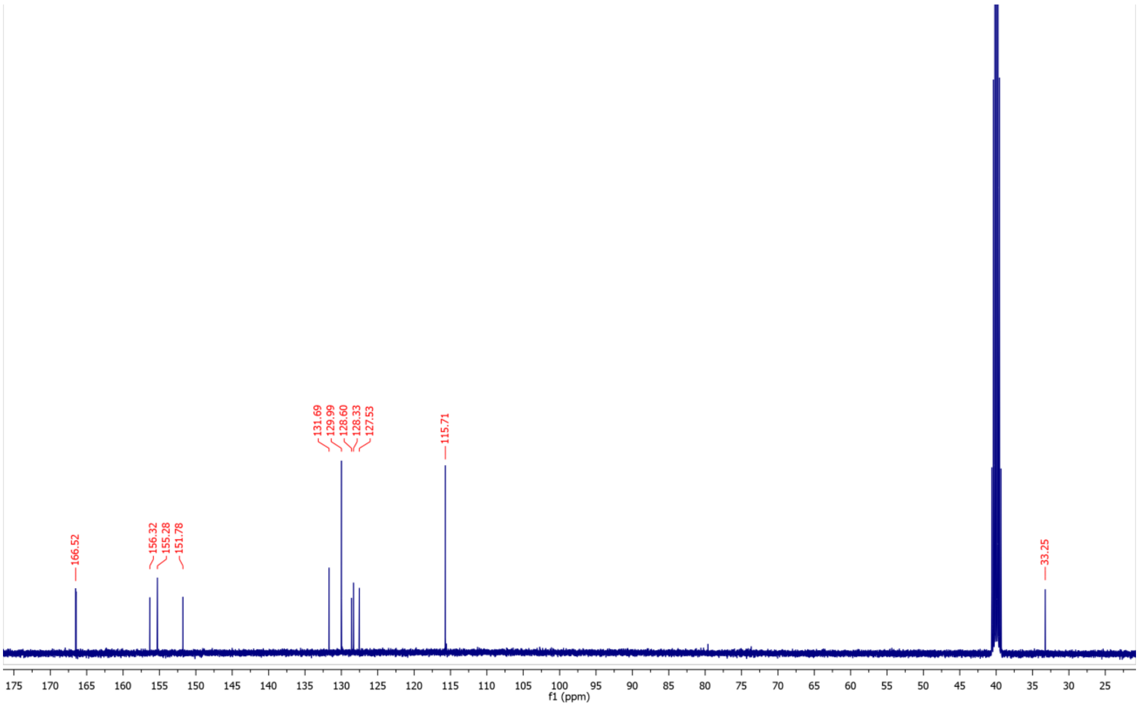

N-(4-Hydroxyphenethyl)morpholine-3,5-dione **(7g)**

White solid; Yield 0% (AcOH), 71% (PEG 400); R_f = 0.36 (CH:EtOAC = 1:1).

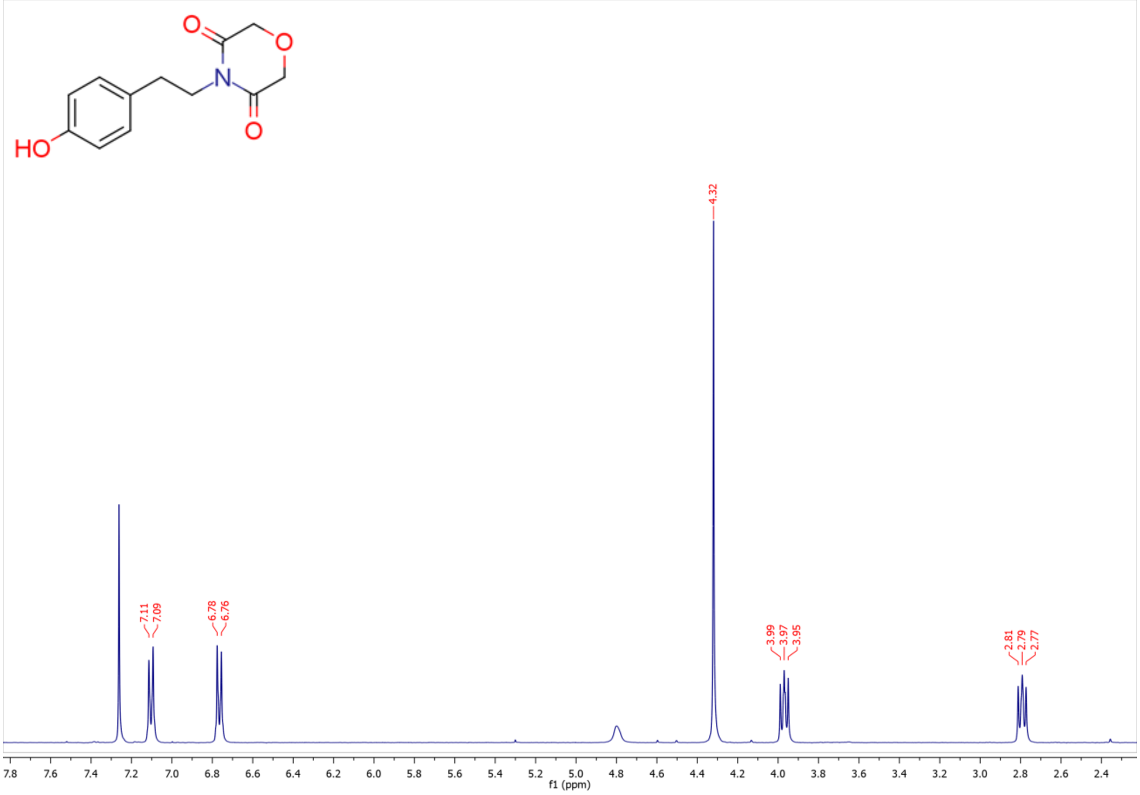

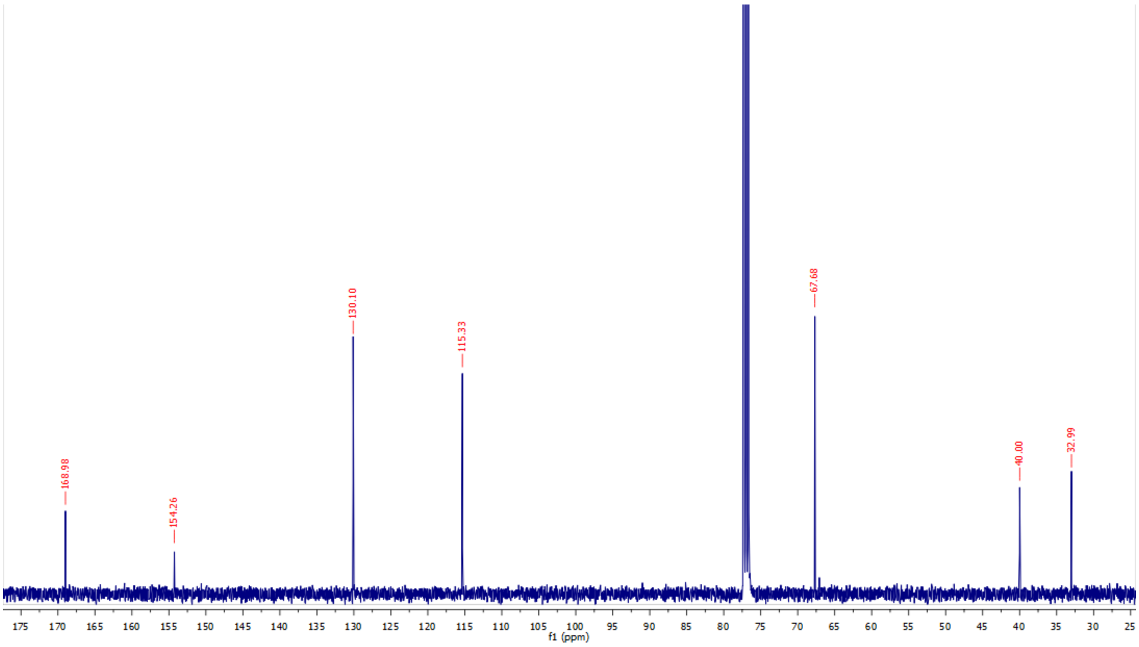

N-(4-Hydroxyphenethyl)hexahydrophthalimide **(7h)**

White solid; Yield 86% (AcOH), 79% (PEG 400); R_f = 0.50 (CH:EtOAC = 1:1).

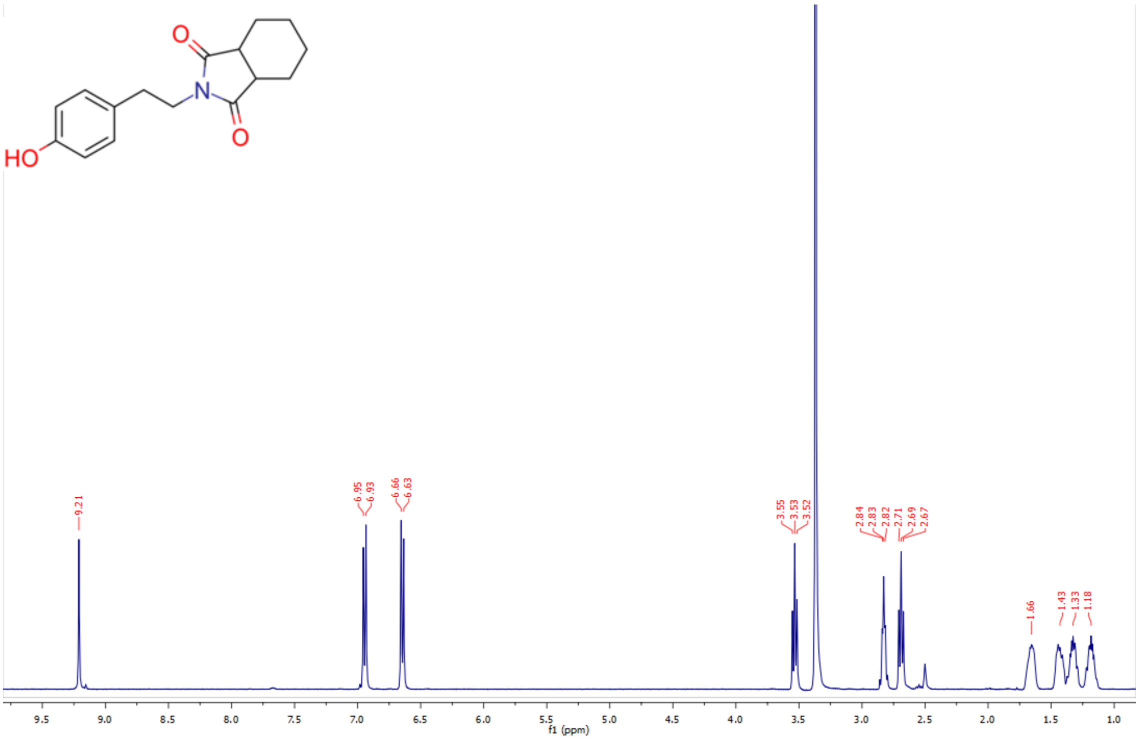

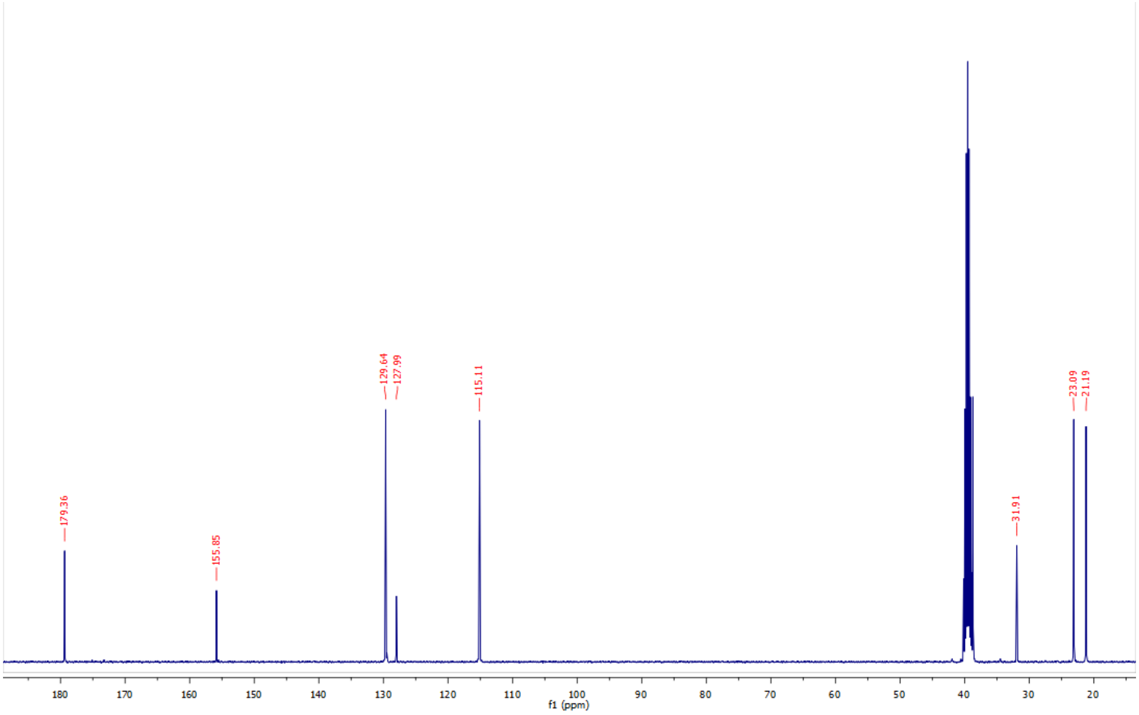

N-(4-Hydroxyphenethyl)-3,4,5,6-tetrahydrophthalimide **(7i)**

White solid; Yield 92% (AcOH), 98% (PEG 400); R_f = 0.41 (CH:EtOAC = 3:1).

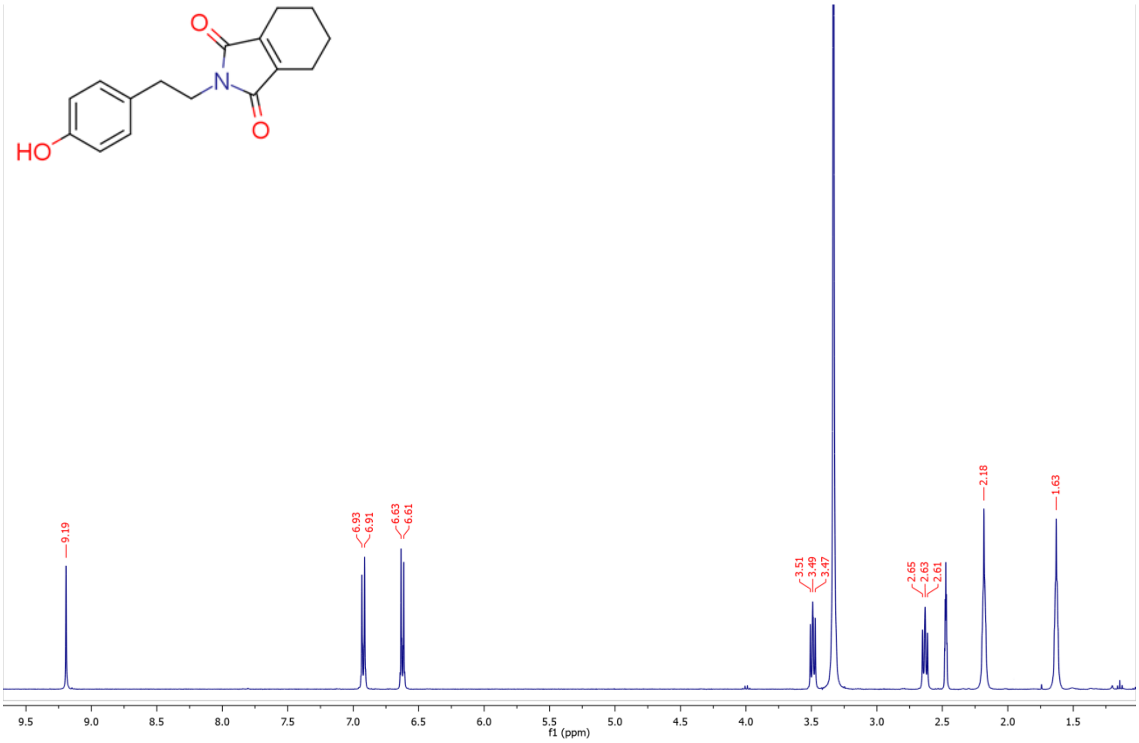

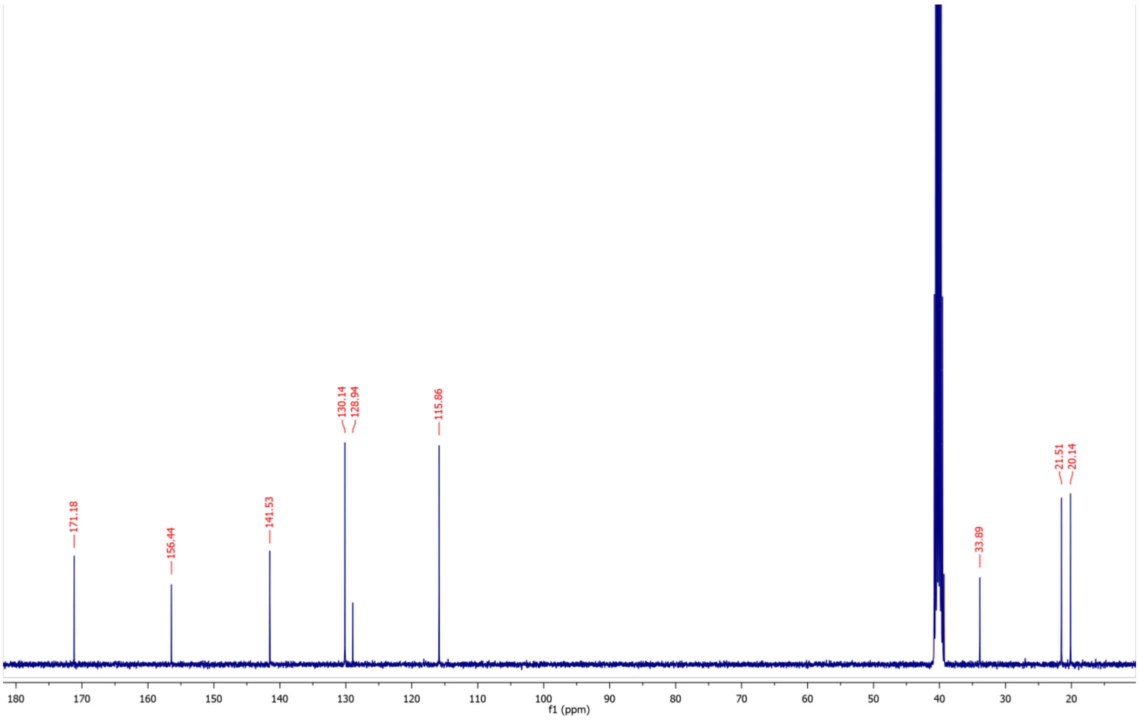

N-(4-Hydroxyphenethyl)-1,2,3,6-tetrahydrophthalimide **(7j)**

White solid; Yield 79% (AcOH), 98% (PEG 400); R_f = 0.32 (CH:EtOAC = 3:1).

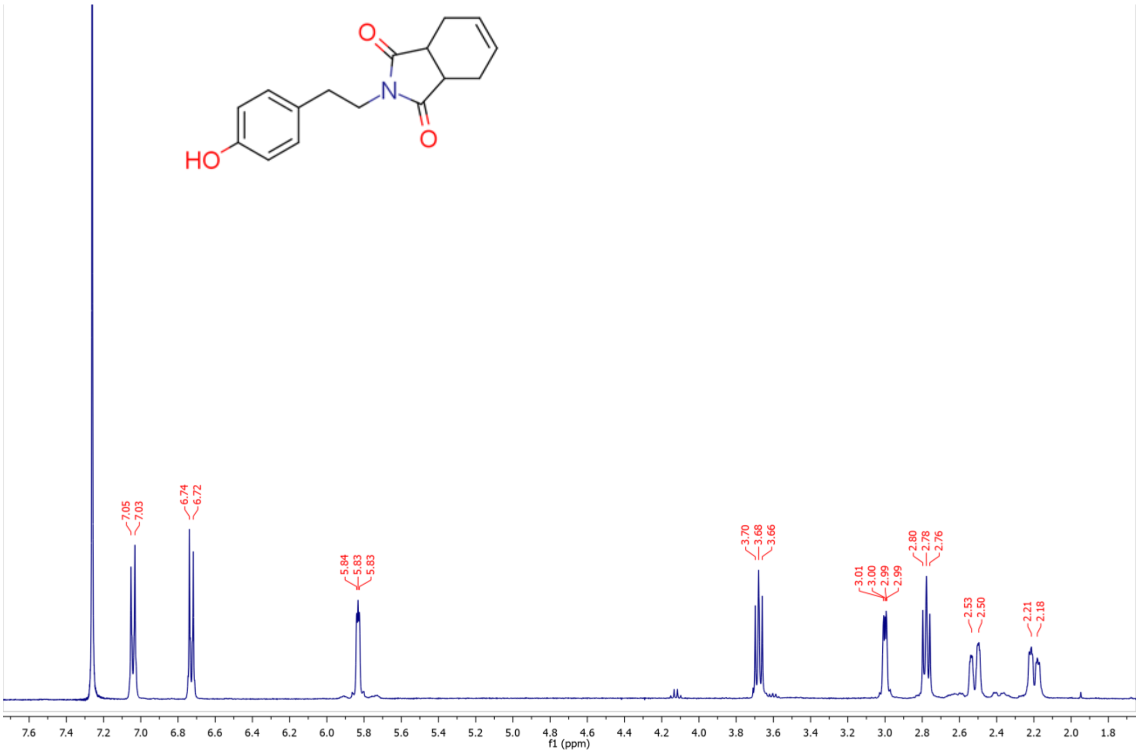

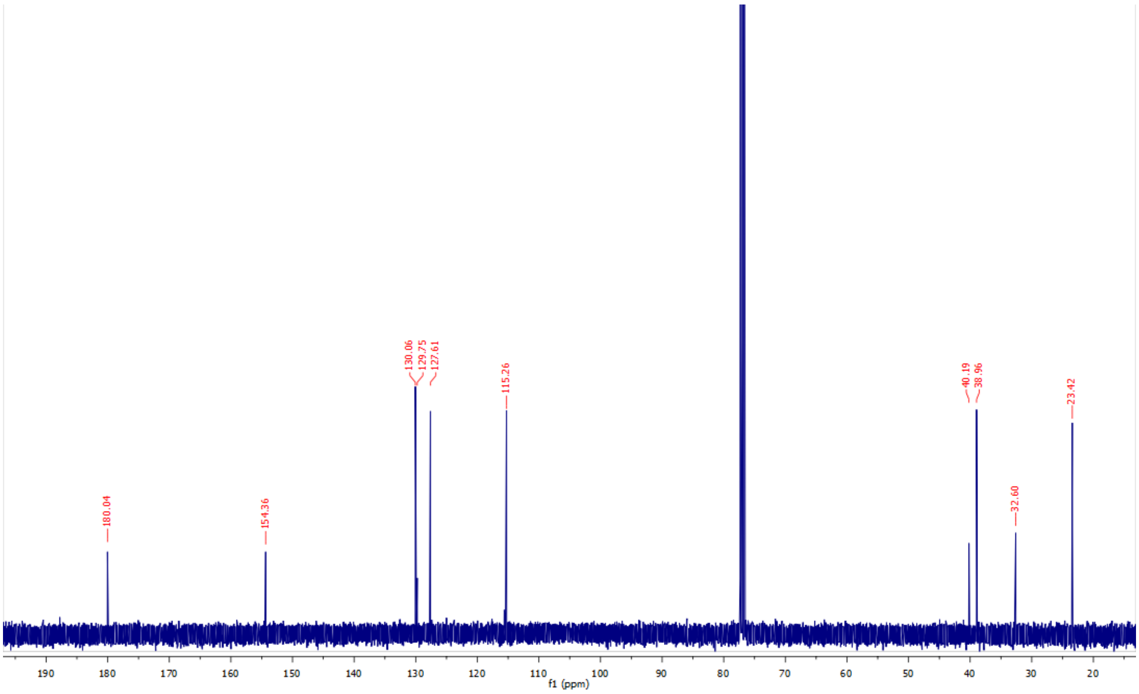

4-(Thexyldimethylsilyloxy)phenethyl bromide **(9)**

Yellow oil, 81%, R_f = 0.70 (CH:EtOAC = 1:1); ^{1}H NMR (400 MHz, $CDCl_3$): δ = 7.05 (d, *J* = 8.4 Hz, 2H, H-2/6), 6.77 (d, *J* = 8.5 Hz, 2H, H-3/5), 3.52 (t, *J* = 7.8 Hz, 2H, H-8), 3.08 (t, *J* = 7.8 Hz, 2H, H-7), 1.72 (hept, *J* = 6.9 Hz, 1H, CH-$(CH_3)_2$), 0.94 (d, *J* = 6.9 Hz, 6H, $(CH_3)_2$-CH), 0.94 (s, 6H, $(CH_3)_2$-C), 0.21 (s, 6H, $(CH_3)_2$-Si) ppm; ^{13}C NMR (100 MHz, $CDCl_3$): δ = 154.4 (C-4), 131.5 (C-1), 129.6 (C-2/6), 120.2 (C-3/5), 38.8 (C-7), 34.1 (CH-$(CH_3)_2$), 33.3 (C-8), 25.0 (C-$(CH_3)_2$), 20.1 ($(CH_3)_2$-C), 18.6 ($(CH_3)_2$-CH), -2.5 ($(CH_3)_2$-Si) ppm; HRMS (EI) Calcd. for $C_{16}H_{27}SiOBr$ $[M]^+$ = 342.1014; Found: 342.1017.

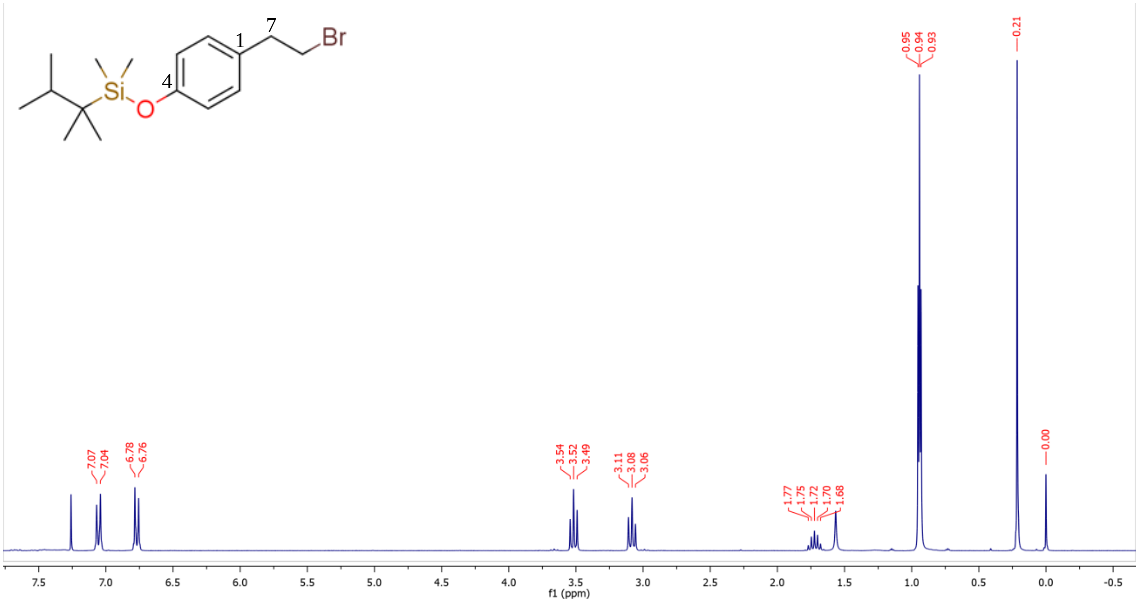

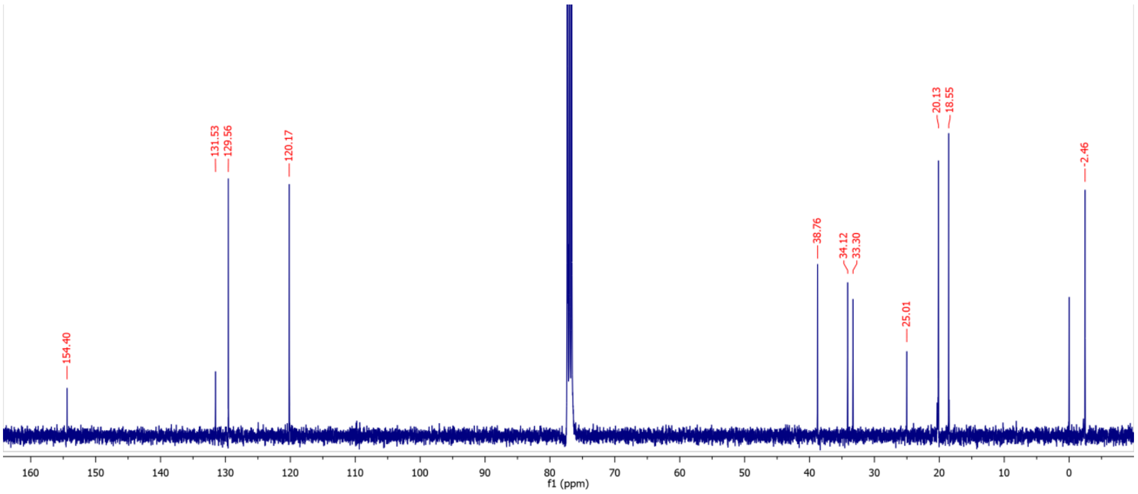

N-(4-Hydroxyphenethyl)isoindoline **(11a)**

White solid; Yield 60% (proton-sponge®), 0% (conventional); R_f = 0.30 (CH:EtOAC = 1:3). ^{1}H NMR (400 MHz, DMSO-d_6): δ = 9.15 (s, 1H, 4-OH), 7.24 – 7.16 (m, 4H, ArH), 7.05 (d, *J* = 8.4 Hz, 2H, H-2/6), 6.67 (d, *J* = 8.3 Hz, 2H, H-3/5), 3.87 (s, 4H, CH_2-N), 2.86 – 2.81 (m, 2H, H-8), 2.69 (t, *J* = 7.7 Hz, 2H, H-7) ppm; ^{13}C NMR (100 MHz, DMSO-d_6): δ = 155.9 (C-4), 140.5 (ArC), 130.7 (C-1), 129.9 (C-2/6), 127.0 (ArC), 122.6 (ArC), 115.4 (C-3/5), 58.9 (CH_2-N), 57.9 (C-8), 34.3 (C-7) ppm; HRMS (EI) calcd. for $C_{16}H_{17}NO$ $[M]^+$ = 239.1310; Found: 239.1303.

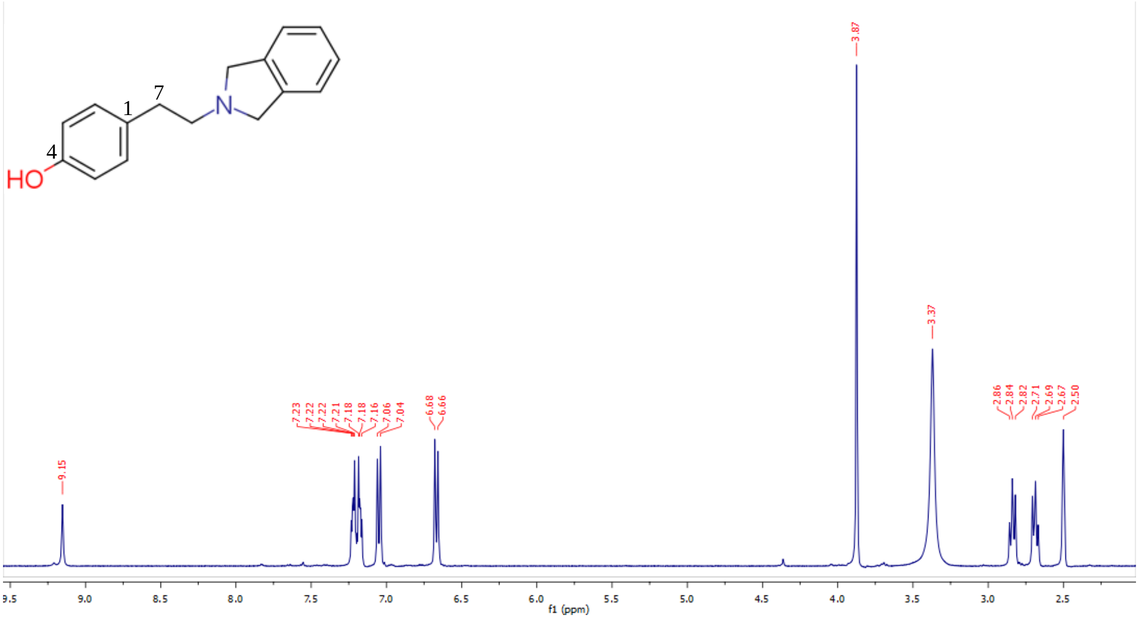

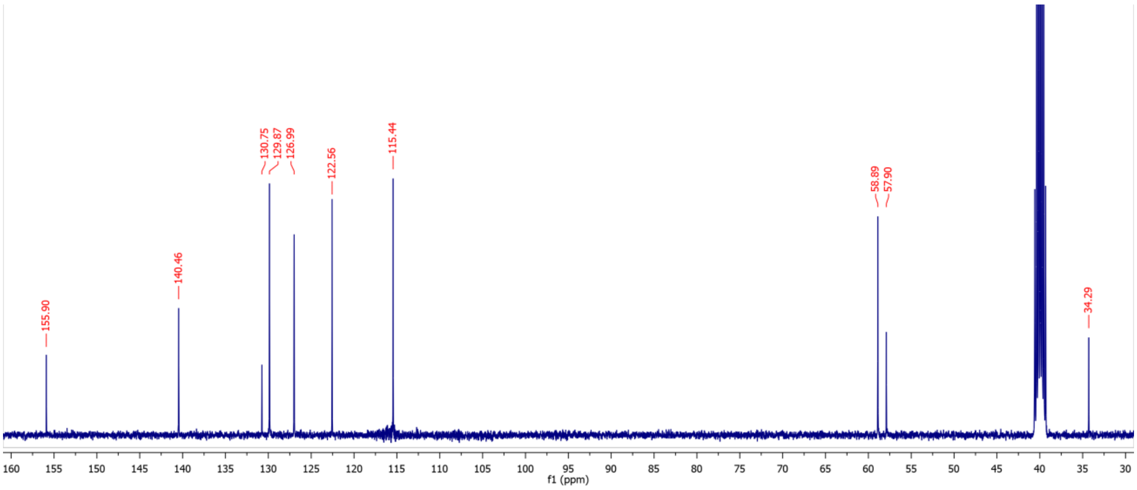

N-(4-Hydroxyphenethyl)pyrrolidine **(11b)**

White solid; Yield 81% (proton-sponge®), 86% (conventional); R_f = 0.22 ($CHCl_3$:MeOH = 1:1); ^{1}H NMR (400 MHz, $CDCl_3$): δ = 6.99 (d, *J* = 8.3 Hz, 2H, H-2/6), 6.63 (d, *J* = 8.3 Hz, 2H, H-3/5), 2.75 (s, 4H, H-7/8), 2.69 – 2.62 (m, 4H, CH_2-N), 1.88 – 1.79 (m, 4H, CH_2-CH_2-N) ppm; ^{13}C NMR (100 MHz, $CDCl_3$): δ = 155.2 (C-4), 130.6 (C-1), 129.5 (C-2/6), 115.7 (C-3/5), 58.6 (C-8), 54.0 (CH_2-N), 34.2 (C-7), 23.3 (CH_2-CH_2-N) ppm; HRMS (EI) calcd. for $C_{12}H_{17}NO$ $[M]^+$ = 191.1310; Found: 191.1304.

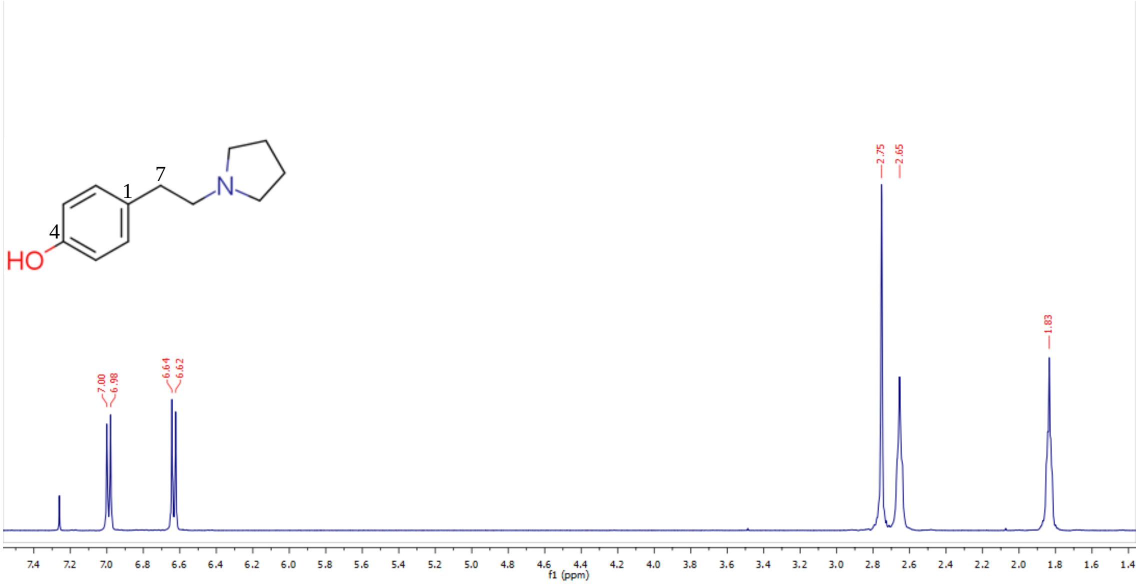

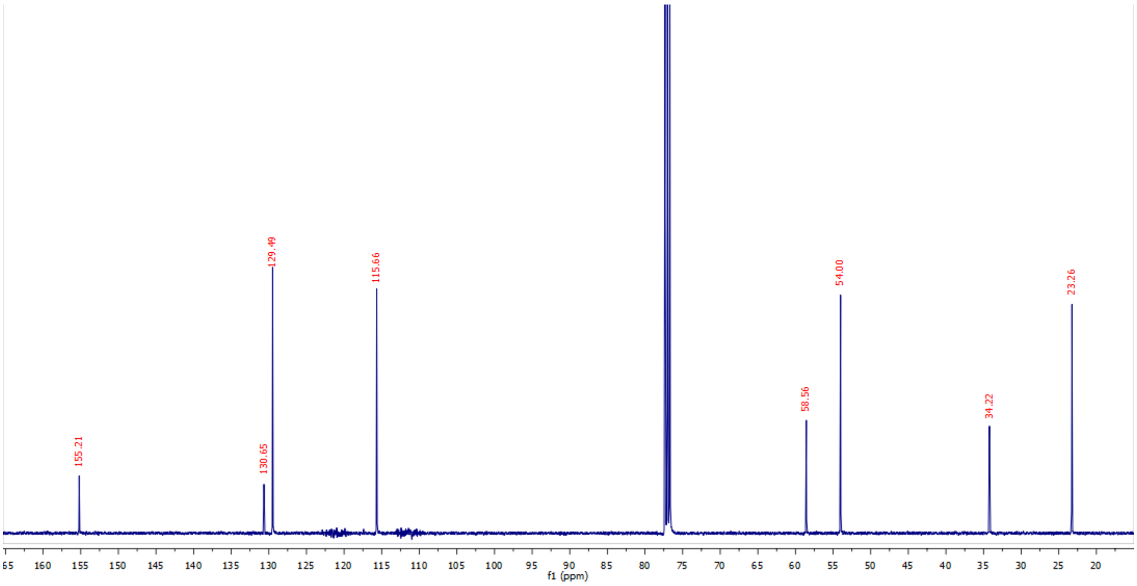

N-(4-Hydroxyphenethyl)morpholine **(11c)**

White solid; Yield 87% (proton-sponge®), 87% (conventional); R_f = 0.27 $CHCl_3$:MeOH (15:1); ^{1}H NMR (400 MHz, DMSO-d_6): δ = 9.14 (s, 1H, 4-OH), 6.99 (d, *J* = 8.5 Hz, 2H, H-2/6), 6.65 (d, *J* = 8.5 Hz, 2H, H-3/5), 3.56 (t, *J* = 4.6 Hz, 4H, CH_2-O), 2.62 – 2.57 (m, 2H, H-7), 2.44 – 2.39 (m, 2H, H-8), 2.41 – 2.35 (m, 4H, CH_2-N) ppm; ^{13}C NMR (100 MHz, DMSO-d_6): δ = 155.9 (C-4), 130.7 (C-1), 129.9 (C-2/6), 115.5 (C-3/5), 66.6 (CH_2-O), 61.1 (C-8), 53.8 (CH_2-N), 32.1 (C-7) ppm; HRMS (EI) calcd. for $C_{12}H_{17}NO_2$ $[M]^+$ = 207.1259; Found: 207.1255.

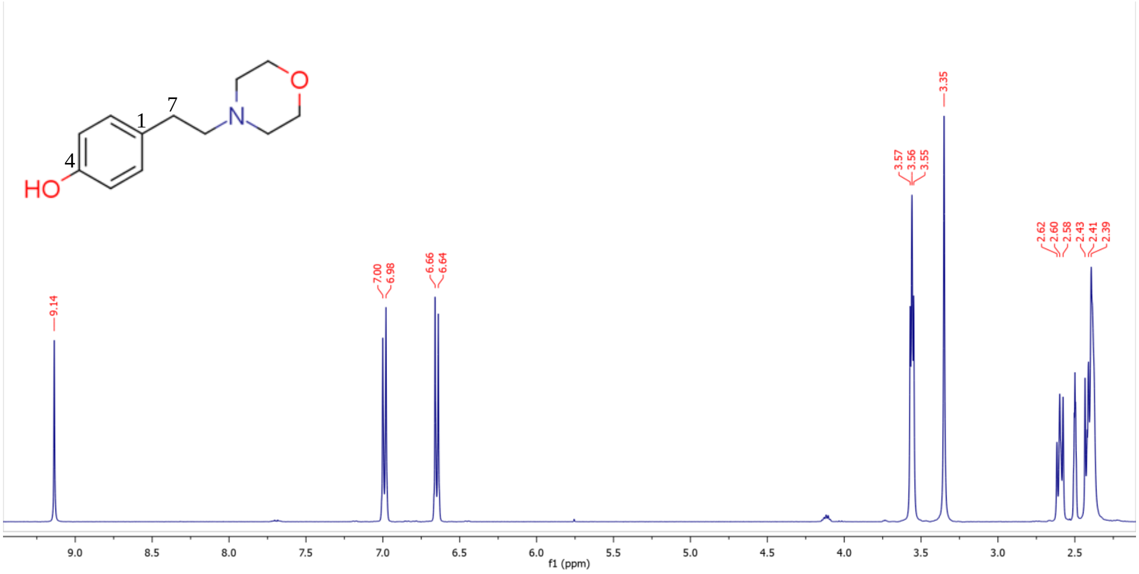

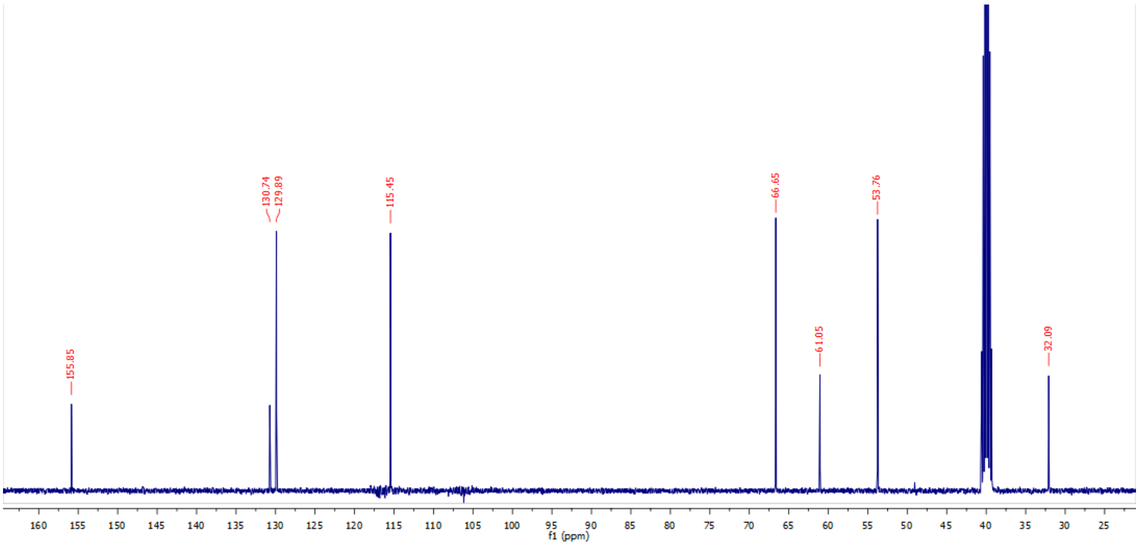

N-(4-Hydroxyphenethyl)octahydroisoindole **(11d)**

White solid; Yield 77% (proton-sponge®), 79% (conventional); R_f = 0.19 ($CHCl_3$:EtOH = 5:2 ^{1}H NMR (400 MHz, $CDCl_3$): δ = 6.93 (d, *J* = 8.3 Hz, 2H, H-2/6), 6.77 (d, *J* = 8.3 Hz, 2H, C-3/5), 3.18 (dd, *J* = 10.7, 6.3 Hz, 2H, $CH_{2(a)}$-N), 3.06 – 3.00 (m, 2H, H-8), 2.93 – 2.87 (m, 2H, $CH_{2(b)}$-N), 2.86 – 2.80 (m, 2H, H-7), 2.34 – 2.24 (m, 2H, CH-CH_2), 1.67 – 1.58 (m, 2H, $CH_{2(a)}$-CH), 1.54 – 1.45 (m, 2H, $CH_{2(a)}$-CH_2-CH), 1.52 – 1.43 (m, 2H, $CH_{2(b)}$-CH), 1.39 – 1.31 (m, 2H, $CH_{2(b)}$-CH_2-CH) ppm; ^{13}C NMR (100 MHz, $CDCl_3$): δ = 156.4 (C-4), 129.5 (C-2/6), 128.1 (C-1), 115.9 (C-3/5), 59.0 (C-8), 57.1 (CH_2-N), 36.7 (CH-CH_2), 32.3 (C-7), 25.9 (CH_2-CH), 22.5 (CH_2-CH_2-CH) ppm; HRMS (EI) calcd. for $C_{16}H_{23}NO$ $[M]^+$ = 245.1780; Found: 245.1772.

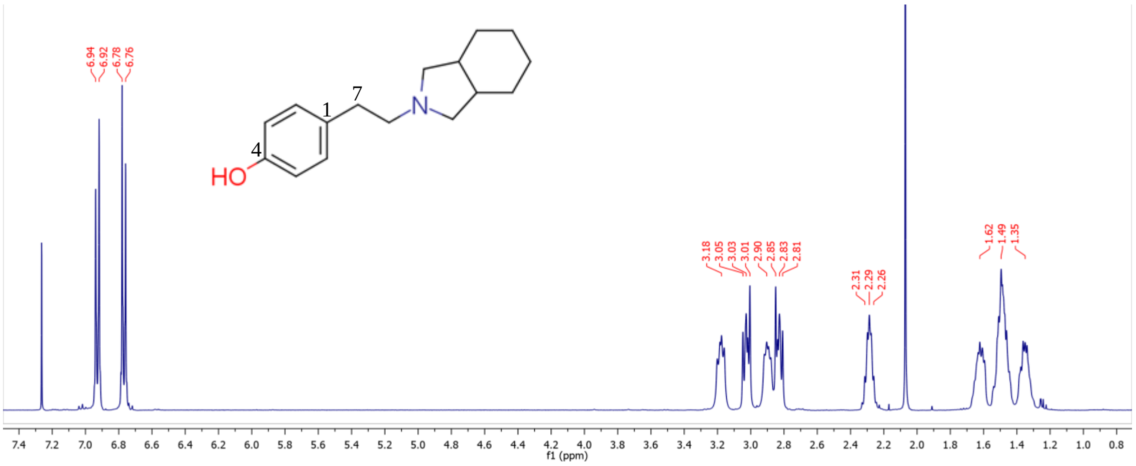

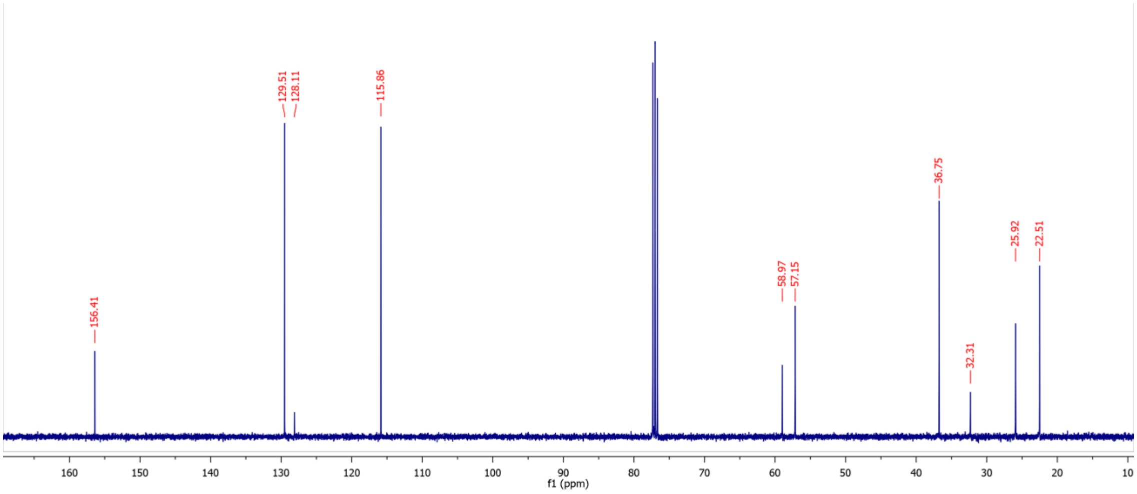

N-(4-Hydroxyphenethyl)-4,5,6,7-tetrahydroisoindole **(11e)**

Slightly yellow oil; Yield 100%; R_f = 0.69 (EtOAc); ^{1}H NMR (400 MHz, $CDCl_3$): δ = 7.00 (d, *J* = 8.2 Hz, 2H, H-2/6), 6.75 (d, *J* = 8.2 Hz, 2H, H-3/5), 6.31 (s, 2H, CH-N), 3.97 – 3.91 (m, 2H, H-8), 3.01 – 2.90 (m, 2H, H-7), 2.59 – 2.52 (m, 4H, CH_2-C=), 1.76 – 1.68 (m, 4H, CH_2-CH_2-C=) ppm; ^{13}C NMR (100 MHz, $CDCl_3$): δ = 154.3 (C-4), 130.7 (C-1), 129.8 (C-2/6), 119.4 (C=C(H)-N), 115.9 (CH-N), 115.4 (C-3/5), 51.3 (C-8), 37.6 (C-7), 24.2 (CH_2-CH_2-C=), 22.0 (CH_2-C=) ppm; HRMS (EI) calcd. for $C_{16}H_{19}NO$ $[M]^+$ = 241.1467; Found: 241.1465.

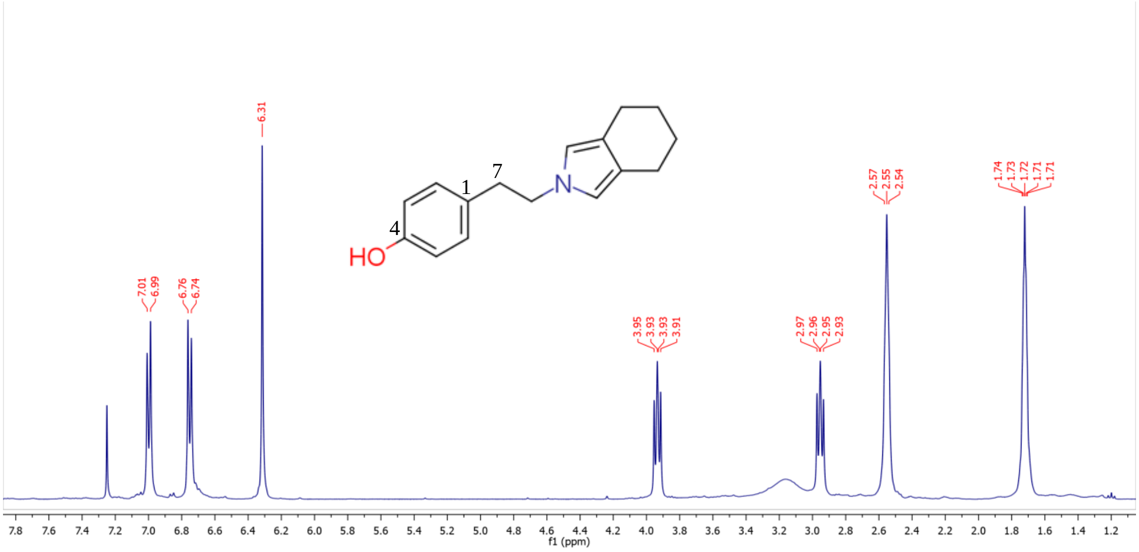

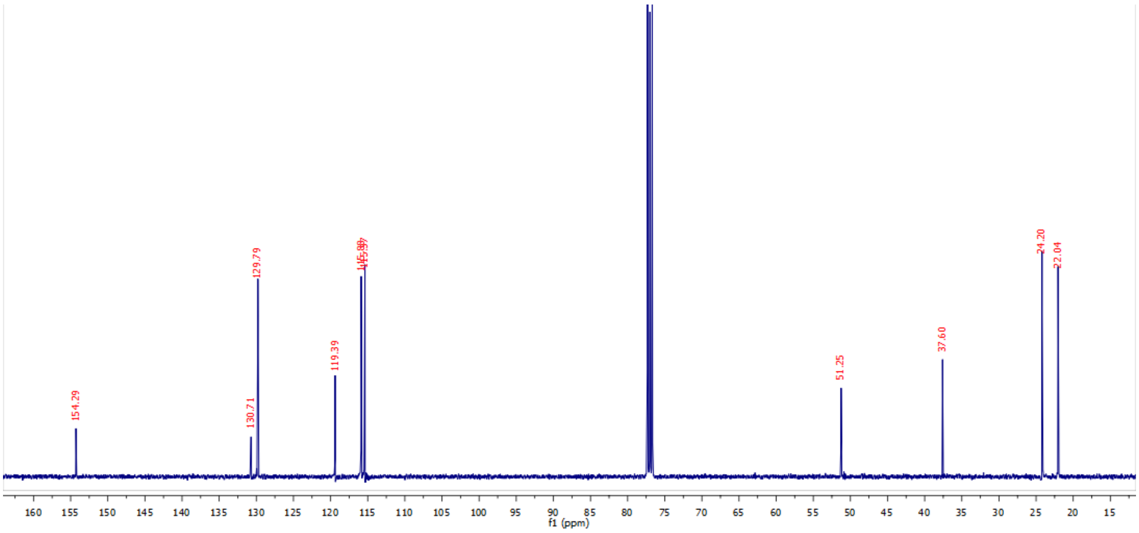

3-Hydroxy-N-(4-hydroxyphenethyl)octahydroisoindole-1-one **(12)**

White solid; Yield 88%; R_f = 0.28 (CH:EtOAc = 1:3).

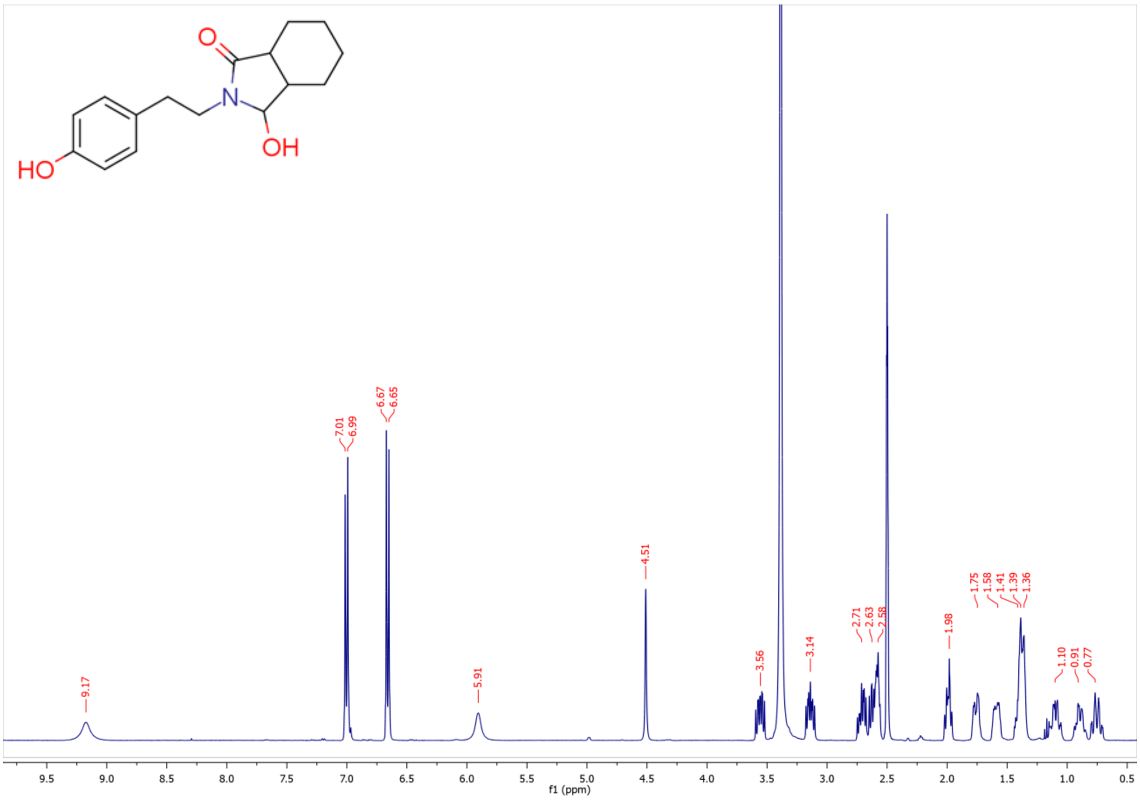

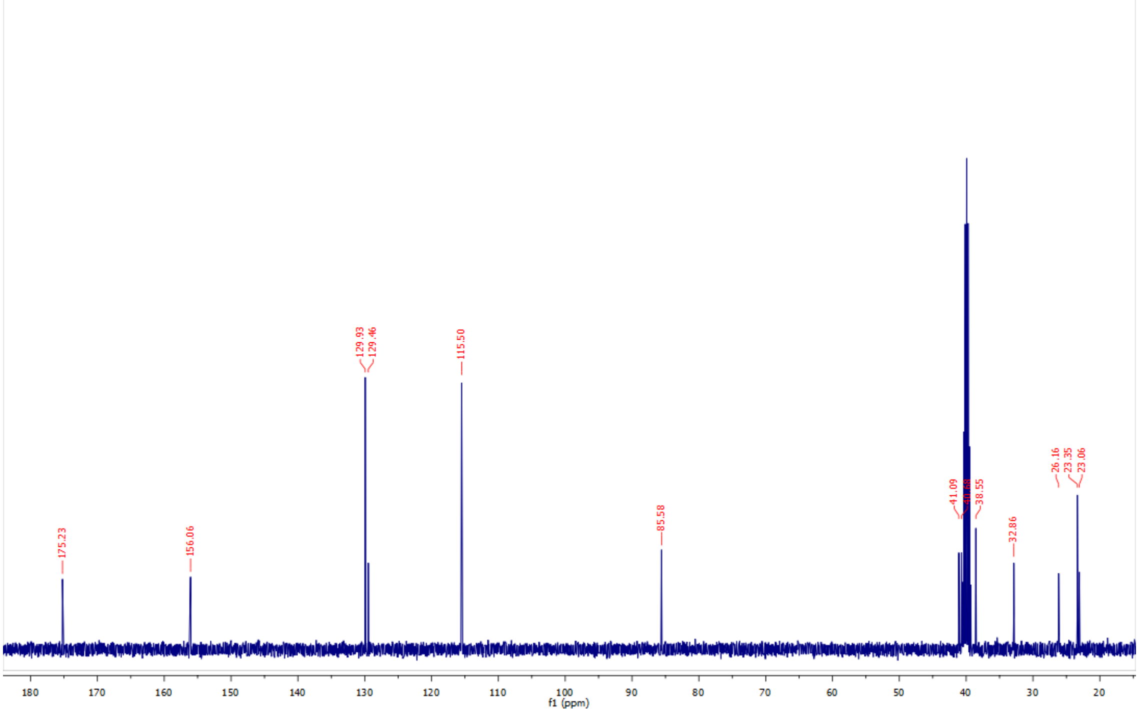

N-[2-(1-Hydroxy-4-oxocyclohexa-2,5-dien-1-yl)ethyl]phthalimide) **(13a)**

White crystals; Yield 67%; R_f = 0.42 (CH:EtOAc = 1:3); mp: 161-162°C; ^{1}H NMR (300 MHz, DMSO-d$_6$): δ = 7.88 – 7.80 (m, 4H, ArH), 6.97 (d, *J* = 10.2 Hz, 2H, H-2/6), 6.10 (d, *J* = 10.2 Hz, 2H, H-3/5), 5.88 (s, 1H, 1-OH), 3.68 – 3.50 (m, 2H, H-8), 2.08 – 1.88 (m, 2H, H-7) ppm; ^{13}C NMR (100 MHz, DMSO-d$_6$): δ = 185.0 (C-4), 167.7 ((CO)N), 152.2 (C-2/6), 134.4 (ArC), 131.7 (ArC), 127.1 (C-3/5), 123.0 (ArC), 67.5 (C-1), 37.8 (C-7), 33.1 (C-8) ppm; HRMS (EI) calcd. for $C_{16}H_{13}NO_4$ $[M]^+$ = 283.0845; Found: 283.0845.

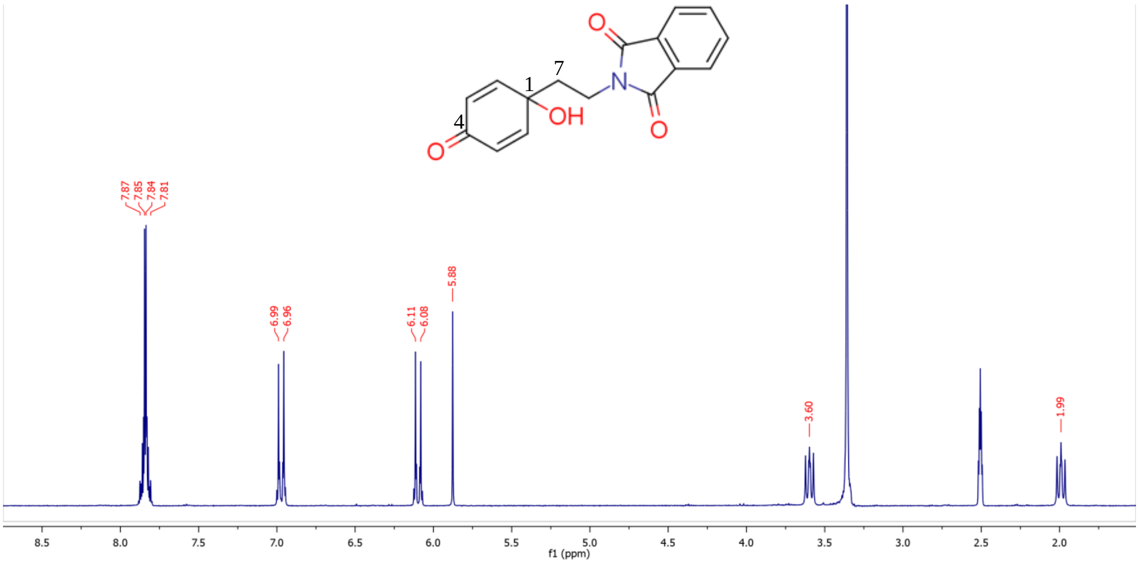

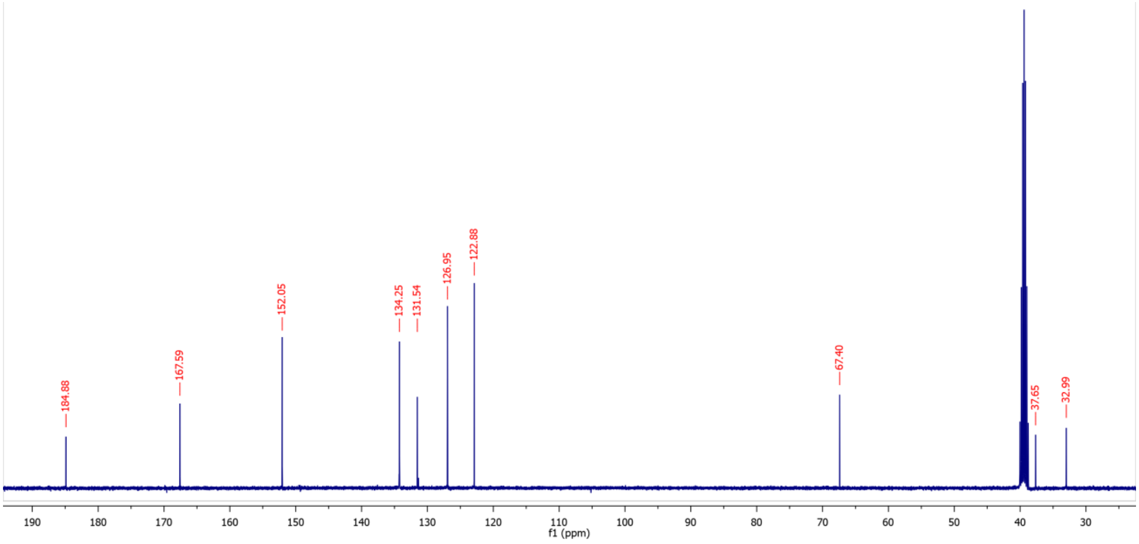

N-[2-(1-Hydroxy-4-oxocyclohexa-2,5-dien-1-yl)ethyl]maleimide **(13b)**

Yellow crystals; Yield 17%; R_f = 0.40 (CH:EtOAc = 1:5); mp: 151-152°C; ^{1}H NMR (300 MHz, DMSO-d$_6$): δ = 6.99 (s, 2H, CH-(CO)N), 6.91 (d, *J* = 10.1 Hz, 2H, H-2/6), 6.08 (d, *J* = 10.1 Hz, 2H, H-3/5), 5.85 (s, 1H, 1-OH), 3.45 – 3.38 (m, 2H, H-8), 1.93 – 1.85 (m, 2H, H-7) ppm; ^{13}C NMR (100 MHz, DMSO-d$_6$): δ = 185.0 (C-4), 170.8 ((CO)N), 152.1 (C-2/6), 134.6 (CH-(CO)N), 127.1 (C-3/5), 67.4 (C-1), 37.9 (C-7), 32.8 (C-8) ppm; HRMS (EI) calcd. for $C_{12}H_{11}NO_4$ $[M]^+$ = 233.0688; Found: 233.0686.

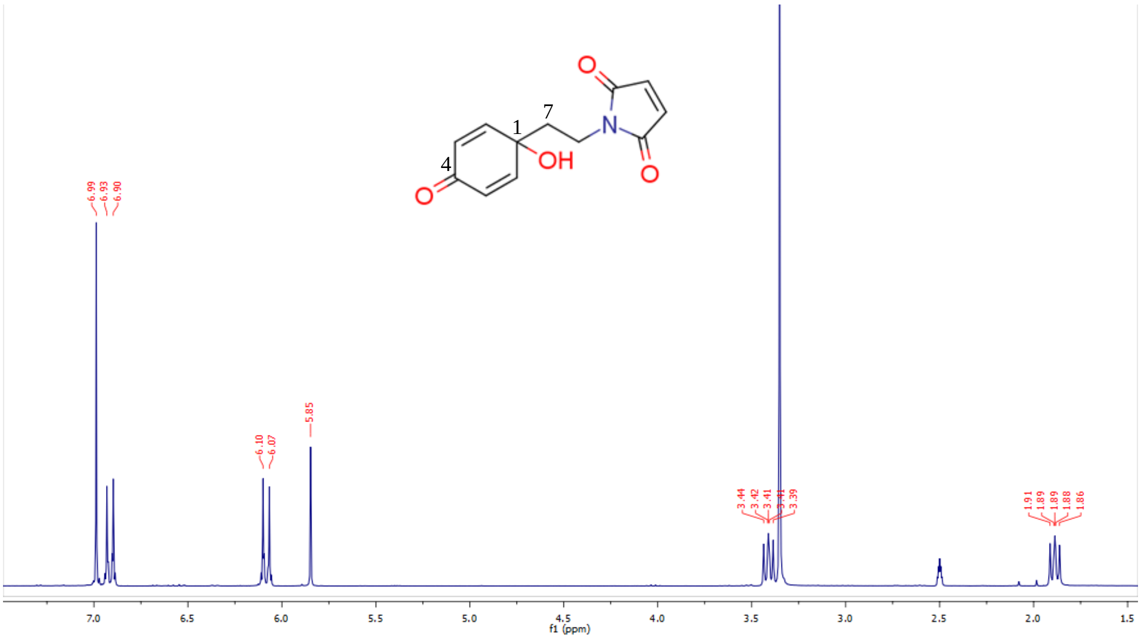

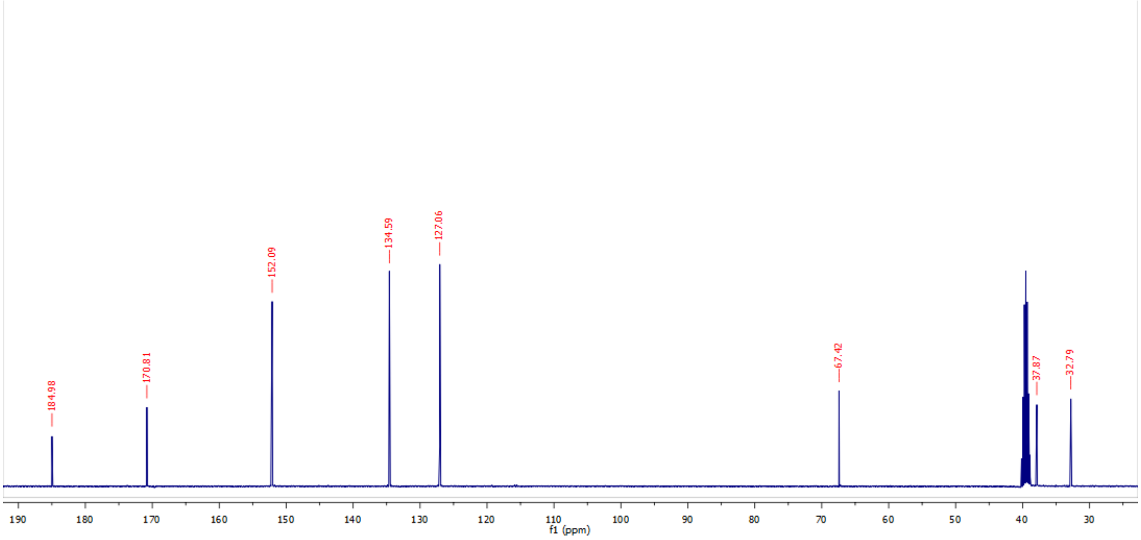

N-[2-(1-Hydroxy-4-oxocyclohexa-2,5-dien-1-yl)ethyl]succinimide **(13c)**

White solid; Yield 55%; R_f = 0.51 ($CHCl_3$:CH_3CN = 1:3); mp: 128-129°C; ^{1}H NMR (400 MHz, DMSO-d_6): δ = 6.93 (d, J = 10.2 Hz, 2H, H-2/6), 6.10 (d, J = 10.2 Hz, 2H, H-3/5), 5.86 (s, 1H, 1-OH), 2.57 (s br, 4H, CH_2-(CO)N), 3.38-3.31 (m, 2H, H-8), 1.89 – 1.77 (m, 2H, H-7) ppm; ^{13}C NMR (100 MHz, DMSO-d_6): δ = 185.5 (C-4), 178.0 ((CO)N), 152.6 (C-2/6), 127.5 (C-3/5), 67.9 (C-1), 37.5 (C-7), 33.9 (C-8), 28.4 (CH_2-(CO)N) ppm; HRMS (EI) calcd. for $C_{12}H_{13}NO_4$ $[M]^+$ = 235.0845; Found: 235.0826.

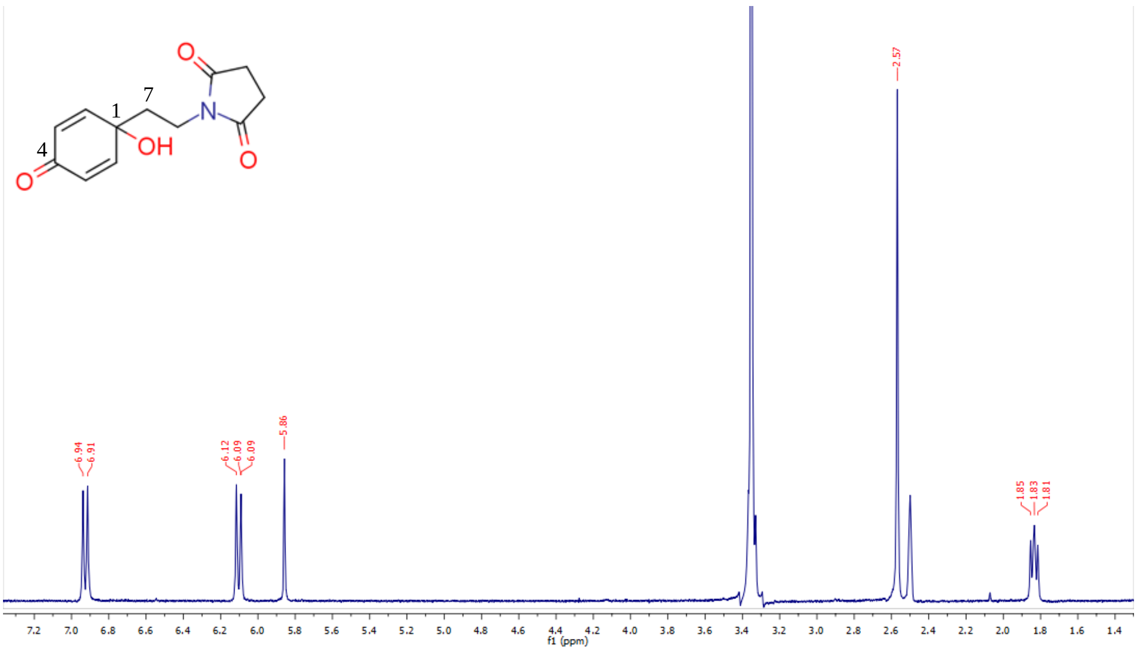

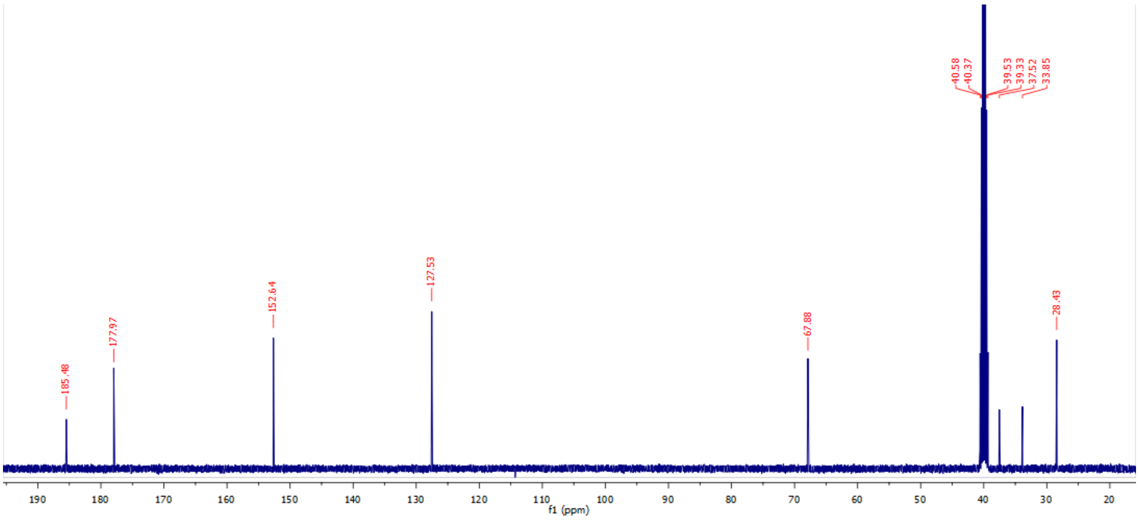

4,5-Dichloro-N-[2-(1-hydroxy-4-oxocyclohexa-2,5-dien-1-yl)ethyl]phthalimide **(13d)**

White crystals; Yield 40%; R_f = 0.22 (CH:EtOAc = 1:1); mp: 213-214°C; ^{1}H NMR (400 MHz, DMSO-d_6): δ = 8.17 (s, 2H, ArH), 6.96 (d, *J* = 10.1 Hz, 2H, H-2/6), 6.10 (d, *J* = 10.1 Hz, 2H, H-3/5), 5.89 (s, 1H, 1-OH), 3.62 – 3.56 (m, 2H, H-8), 2.02 – 1.95 (m, 2H, H-7) ppm; ^{13}C NMR (100 MHz, DMSO-d_6): δ = 185.5 (C-4), 166.4 ((CO)N), 152.6 (C-2/6), 137.7 (C(Cl)=), 132.1 (C=C(CO)N), 127.6 (C-3/5), 125.6 (ArC), 67.9 (C-1), 38.0 (C-7), 34.0 (C-8) ppm; HRMS (EI) calcd. for $C_{16}H_{11}Cl_2NO_4$ $[M]^+$ = 351.0065; Found: 351.0090.

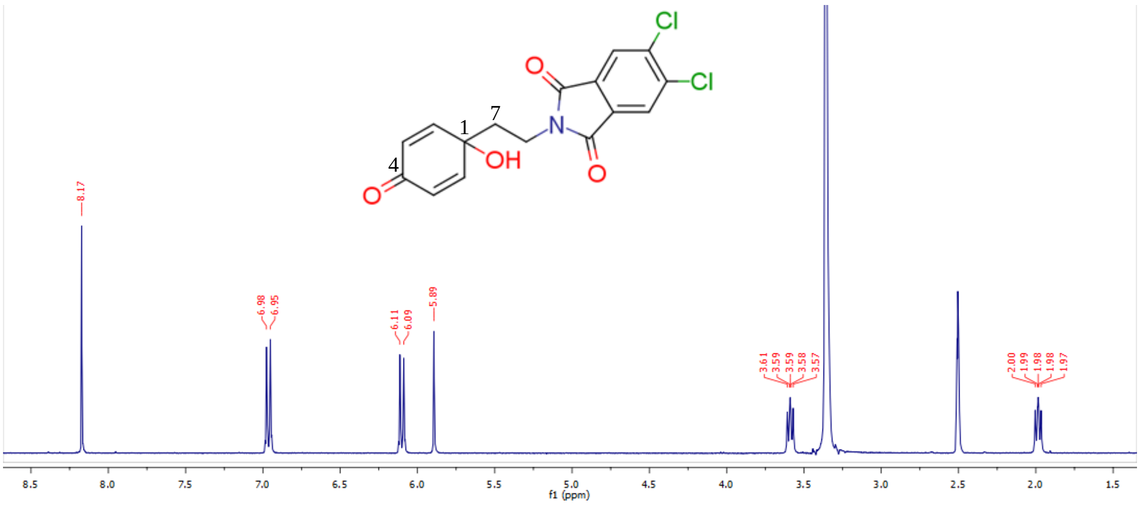

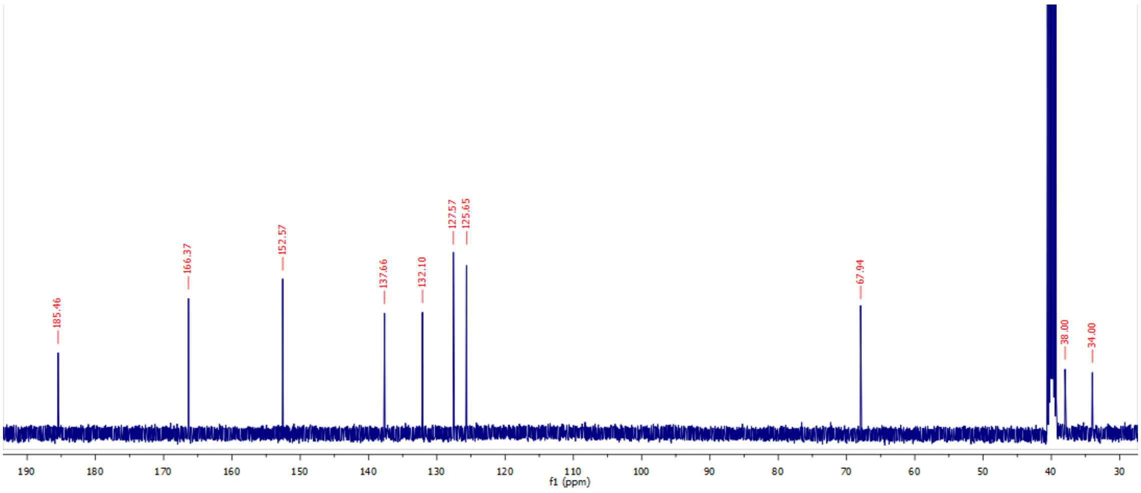

3,4-Dichloro-N-[2-(1-hydroxy-4-oxocyclohexa-2,5-dien-1-yl)ethyl]maleimide **(13e)**

Yellowish crystals; Yield 64%; R_f = 0.30 (CH:EtOAc = 1:1); mp: 168-169°C; ^{1}H NMR (400 MHz, DMSO-d_6): δ = 6.95 (d, J = 10.1 Hz, 2H, H-2/6), 6.11 (d, J = 10.1 Hz, 2H, H-3/5), 5.91 (s br, 1H, 1-OH), 3.53 – 3.46 (m, 2H, H-8), 1.95 – 1.90 (m, 2H, H-7) ppm; ^{13}C NMR (100 MHz, DMSO-d_6): δ = 185.4 (C-4), 163.3 ((CO)N), 152.5 (C-2/6), 132.9 (C(Cl)=), 127.6 (C-3/5), 67.8 (C-1), 37.9 (C-7), 34.8 (C-8) ppm; HRMS (EI) calcd. for $C_{12}H_9Cl_2NO_4$ $[M]^+$ = 300.9909; Found: 300.9914.

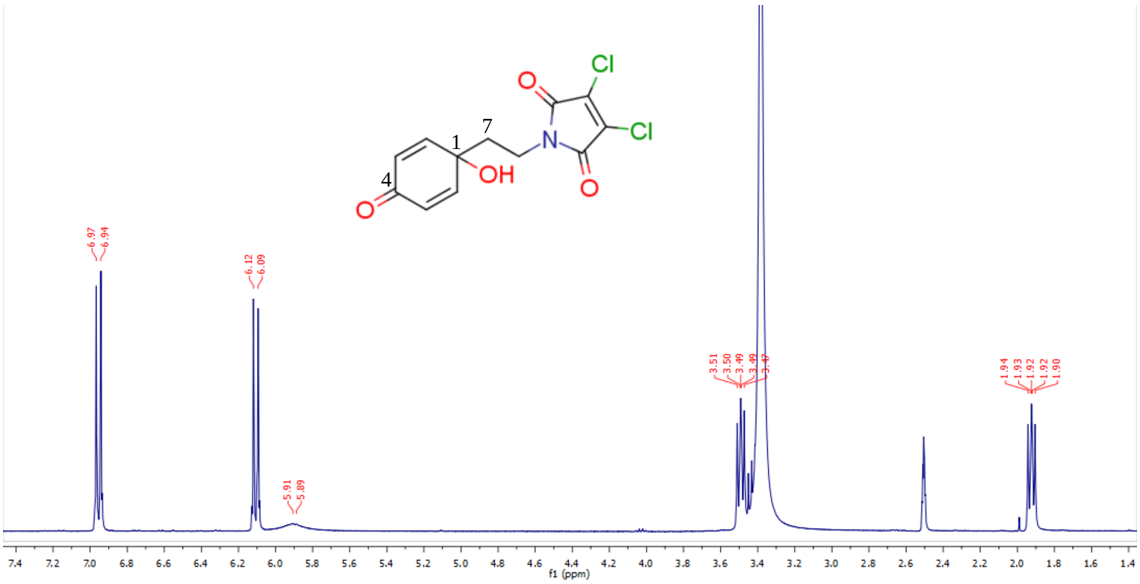

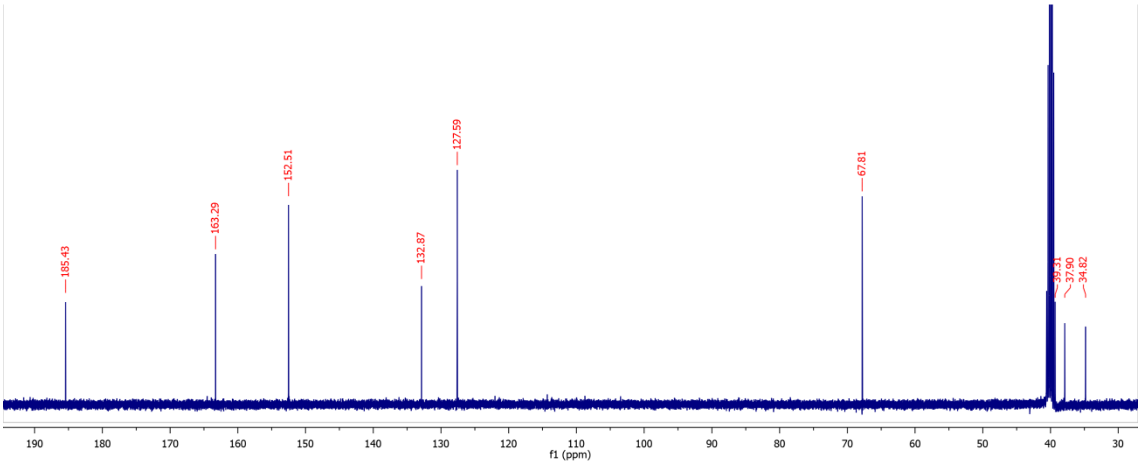

N-[2-(1-Hydroxy-4-oxocyclohexa-2,5-dien-1-yl)ethyl]pyridine-2,3-dicarboxímide **(13f)**

White crystals; Yield 19%; R_f = 0.37 ($CHCl_3$:CH_3CN = 1:1); mp: 167-168°C; ^{1}H NMR (400 MHz, DMSO-d_6): δ = 8.95 (dd, *J* = 5.0, 1.5 Hz, 1H, ArH), 8.27 (dd, *J* = 7.7, 1.5 Hz, 1H, ArH), 7.77 (dd, *J* = 7.7, 5.0 Hz, 1H, ArH), 6.98 (d, *J* = 10.1 Hz, 2H, C-2/6), 6.11 (d, *J* = 10.1, 2H, H-3/5), 5.88 (s, 1H, 1-OH), 3.69 – 3.57 (m, 2H, C-8), 2.05 – 1.94 (m, 2H, H-7) ppm; ^{13}C NMR (100 MHz, DMSO-d_6): δ = 185.5 (C-4), 166.6 ((CO)N), 155.2 (ArC), 152.6 (C-2/6), 152.0 (ArC), 131.6 (ArC), 128.3 (ArC), 127.7 (ArC), 127.6 (C-3/5), 68.0 (C-1), 38.1 (C-7), 33.7 (C-8) ppm; HRMS (EI) calcd. for $C_{15}H_{12}N_2O_4$ $[M]^+$ = 284.0797; Found: 284.0792.

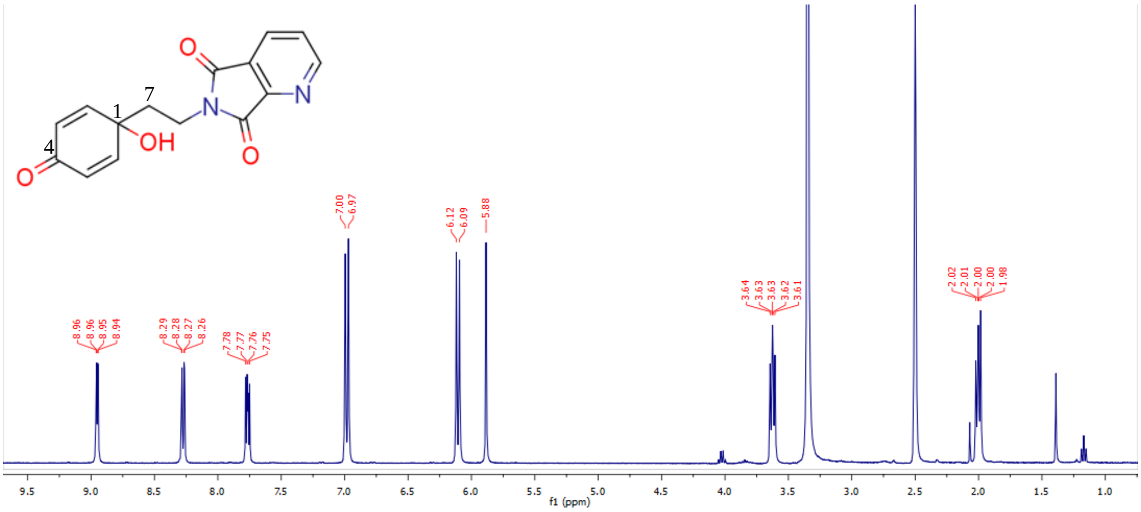

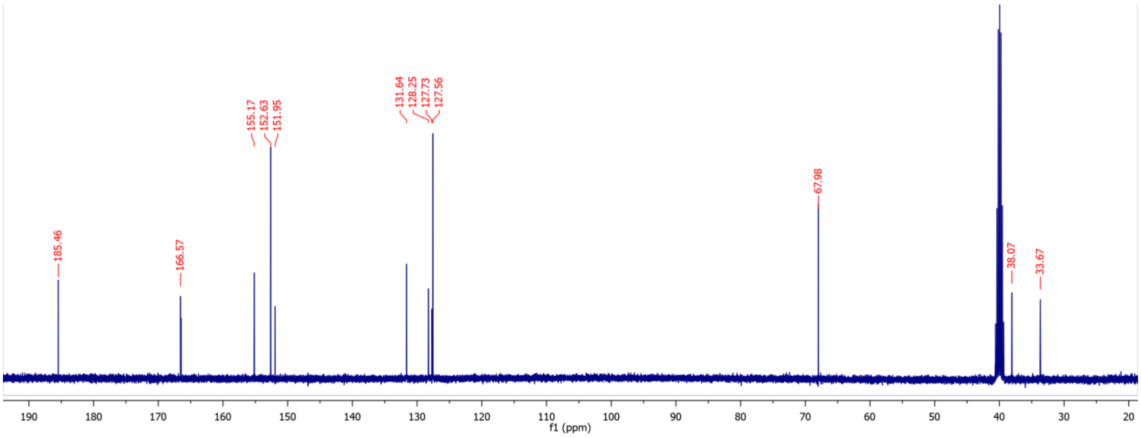

N-[2-(1-Hydroxy-4-oxocyclohexa-2,5-dien-1-yl)ethyl]morpholine-3,5-dione **(13g)**

Yellowish solid; Yield 18%; R_f = 0.33 ($CHCl_3$:EtOAc = 1:5); mp: 142-143°C; ^{1}H NMR (400 MHz, DMSO-d_6): δ = 6.94 (d, *J* = 10.1 Hz, 2H, H-2/6), 6.12 (d, *J* = 10.0 Hz, 2H, H-3/5), 4.36 (s, 4H, CH_2-(CO)N), 3.70 – 3.56 (m, 2H, H-8), 1.89 – 1.78 (m, 2H, H-7) ppm; ^{13}C NMR (100 MHz, DMSO-d_6): δ = 185.5 (C-4), 170.1 ((CO)N), 152.7 (C-2/6), 127.5 (C-3/5), 68.0 (C-1), 67.4 (CH_2-(CO)N), 37.8 (C-7), 33.8 (C-8) ppm; HRMS (EI) calcd. for $C_{12}H_{13}NO_5$ $[M]^+$ = 251.0794; Found: 251.0794.

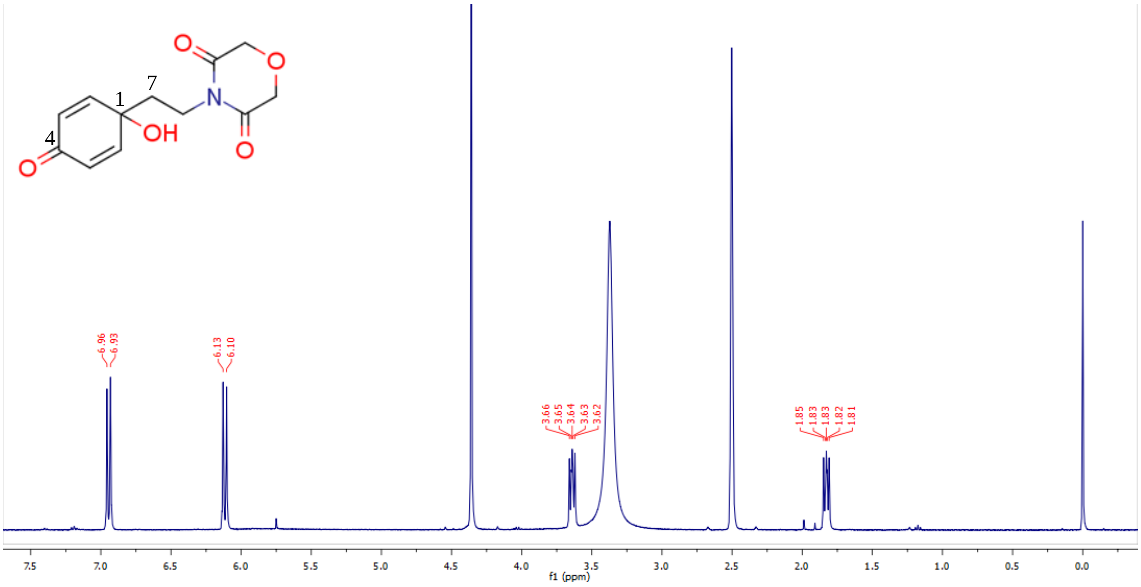

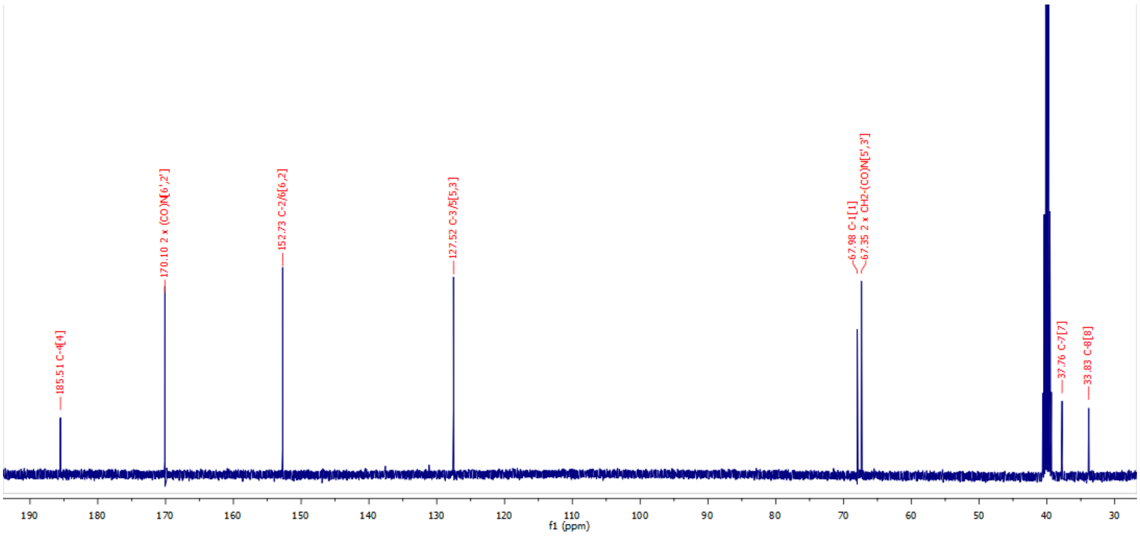

N-[2-(1-Hydroxy-4-oxocyclohexa-2,5-dien-1-yl)ethyl]hexahydrophthalimide **(13h)**

Yellow crystals; Yield 79%; R_f = 0.29 (CH:EtOAc = 1:3); mp: 135-136°C; ^{1}H NMR (400 MHz, DMSO-d_6): δ = 6.94 (d, *J* = 10.1 Hz, 2H, H-2/6), 6.10 (d, *J* = 10.1 Hz, 2H, H-3/5), 5.84 (s, 1H, 1-OH), 3.40 – 3.35 (m, 2H, H-8), 2.93 – 2.82 (m, 2H, CH-(CO)N), 1.85 – 1.80 (m, 2H, H-7), 1.71 (s, 2H, $CH_{2(a)}$-CH), 1.60 – 1.51 (m, 2H, $CH_{2(b)}$-CH), 1.42 – 1.32 (m, 2H, $CH_{2(a)}$-CH_2-CH), 1.31 – 1.21 (m, 2H, $CH_{2(b)}$-CH_2-CH) ppm; ^{13}C NMR (100 MHz, DMSO-d_6): δ = 185.4 (C-4), 179.7 ((CO)N), 152.7 (C-2/6), 127.5 (C-3/5), 67.9 (C-1), 39.3 (CH-(CO)N), 37.6 (C-7), 33.8 (C-8), 23.5 (CH_2-CH), 21.6 (CH_2-CH_2-CH) ppm; HRMS (EI) calcd. for $C_{16}H_{19}NO_4$ $[M]^+$ = 289.1314; Found: 289.1310.

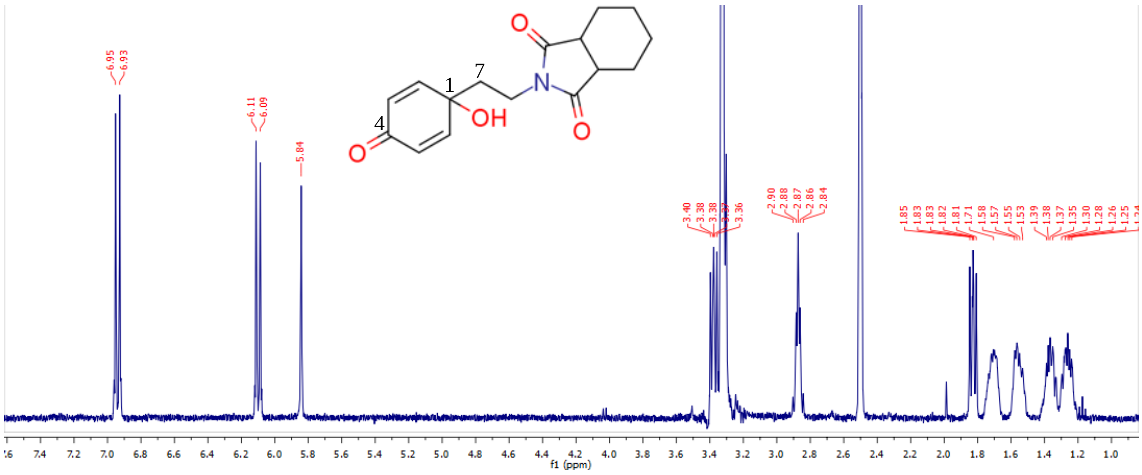

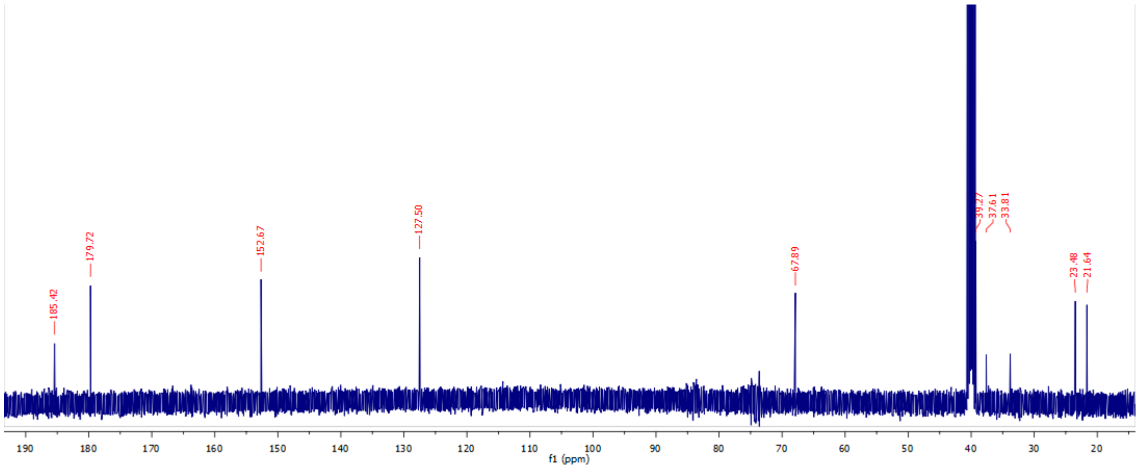

N-[2-(1-Hydroxy-4-oxocyclohexa-2,5-dien-1-yl)ethyl]-3,4,5,6-tetrahydrophthalimide **(13i)**

Orange solid; Yield 88%; R_f = 0.18 (CH:EtOAc = 1:1); mp: 93-94°C; ^{1}H NMR (400 MHz, DMSO-d$_6$): δ = 6.92 (d, *J* = 10.1 Hz, 2H, H-2/6), 6.08 (d, *J* = 10.1 Hz, 2H, H-3/5), 5.85 (s, 1H, 1-OH), 3.42 – 3.36 (m, 2H, H-8), 2.24 – 2.17 (m, 4H, CH_2-C=), 1.86 (dd, *J* = 8.5, 6.8 Hz, 2H, H-7), 1.69 – 1.62 (m, 4H, CH_2-CH_2-C=) ppm; ^{13}C NMR (100 MHz, DMSO-d$_6$): δ = 185.5 (C-4), 170.9 ((CO)N), 152.6 (C-2/6), 141.5 (C=C(CO)), 127.5 (C-3/5), 67.9 (C-1), 38.6 (C-7), 33.0 (C-8), 21.3 (CH_2-CH_2-C=), 19.9 (CH_2-C=) ppm; HRMS (EI) calcd. for $C_{16}H_{17}NO_4$ $[M]^+$ = 287.1158; Found: 287.1160.

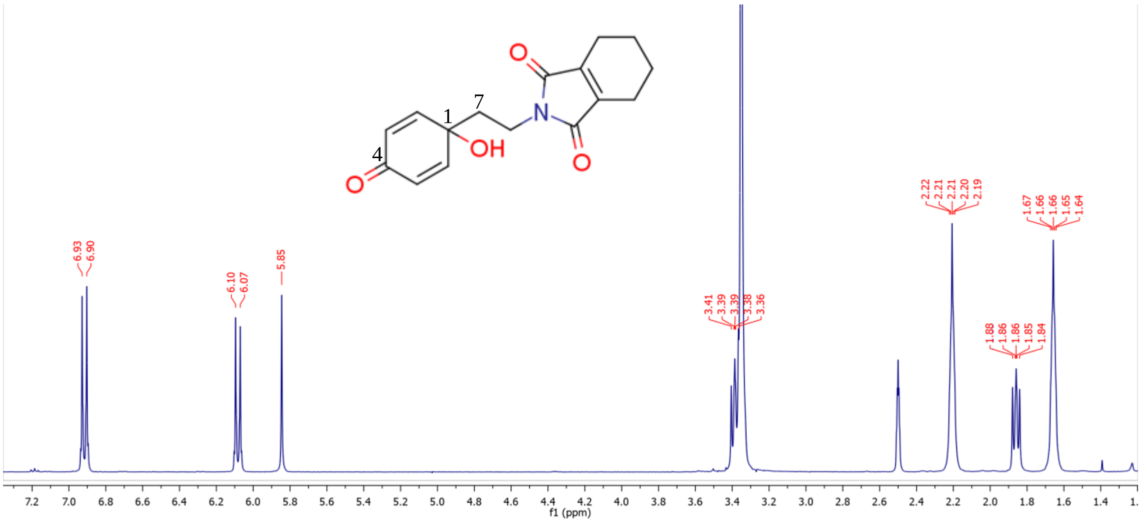

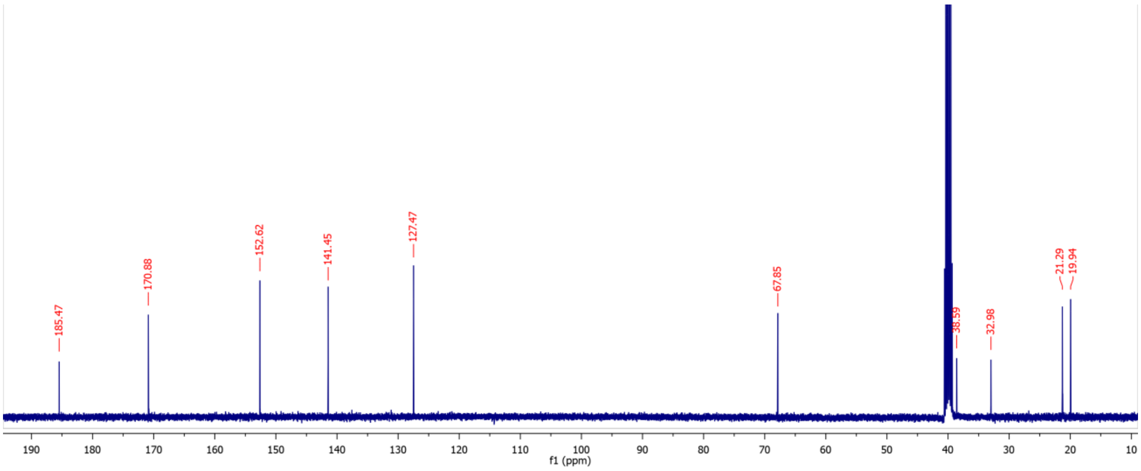

N-[2-(1-Hydroxy-4-oxocyclohexa-2,5-dien-1-yl)ethyl]-1,2,3,6-tetrahydrophthalimide **(13j)**

White crystals; Yield 49%; R_f = 0.28 (CH:EtOAc = 1:1); mp: 145-146°C; ^{1}H NMR (400 MHz, DMSO-d_6): δ = 6.90 (d, *J* = 10.1 Hz, 2H, H-2/6), 6.09 (d, *J* = 10.1 Hz, 2H, H-3/5), 5.87 (s, 1H, 1-OH), 5.85 – 5.82 (m, 2H, CH=CH), 3.31 - 3.36 (m, 2H, H-8), 3.11 – 3.06 (m, 2H, CH-(CO)N), 2.39 – 2.32 (m, 2H, $CH_{2(a)}$-CH), 2.21 – 2.13 (m, 2H, $CH_{2(b)}$-CH), 1.79 (td, *J* = 7.5, 1.5 Hz, 2H, H-7) ppm; ^{13}C NMR (100 MHz, DMSO-d_6): δ = 185.5 (C-4), 180.3 ((CO)N), 152.5 (C-2/6), 128.1 (CH=CH), 127.6 (C-3/5), 67.8 (C-1), 38.9 (CH-(CO)N), 37.8 (C-7), 34.1 (C-8), 23.5 (CH_2-CH) ppm; HRMS (EI) calcd. for $C_{16}H_{17}NO_4$ $[M]^+$ = 287.1158; Found: 287.1154.

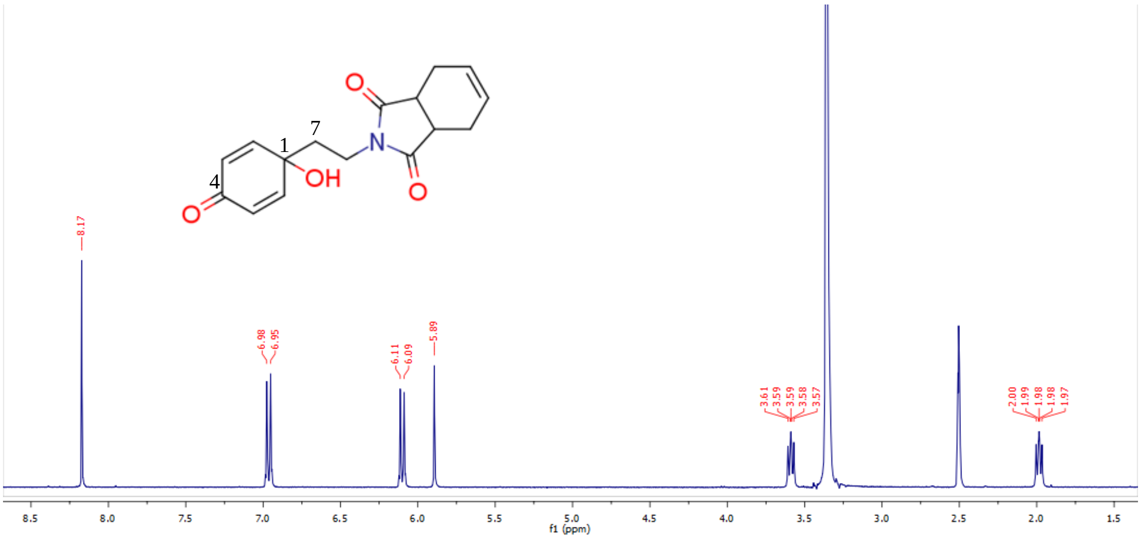

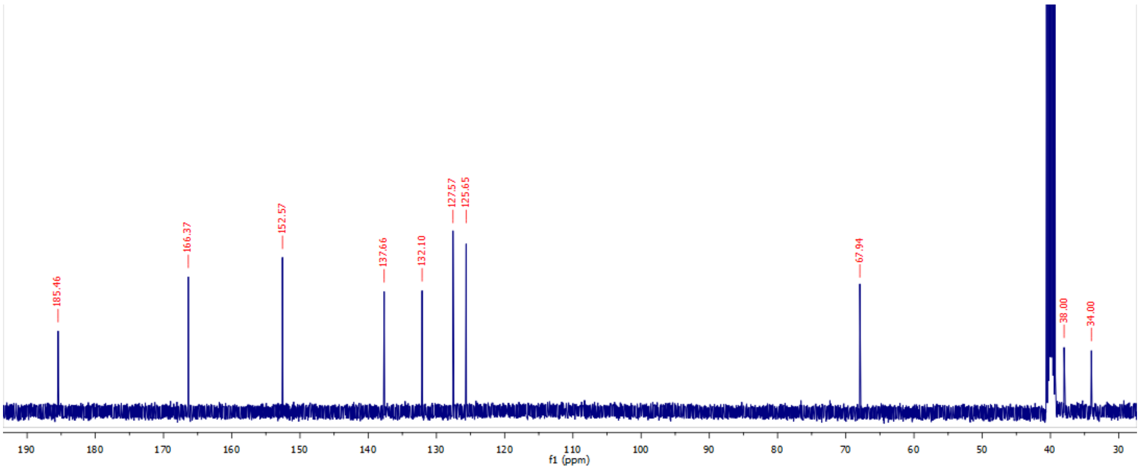

N-[2-(1-Hydroxy-4-oxocyclohexa-2,5-dien-1-yl)ethyl]isoindoline **(14a)**

Brownish solid; Yield 16%; R_f = 0.50 ($CHCl_3$:EtOH = 1:5); mp: 103-104°C; ^1H NMR (400 MHz, DMSO-d_6): δ = 7.24 – 7.12 (m, 4H, ArH), 6.99 (d, *J* = 10.1 Hz, 2H, H-2/6), 6.06 (d, *J* = 10.1 Hz, 2H, H-3/5), 3.81 – 3.77 (m, 4H, CH_2-N), 2.70 – 2.62 (m, 2H, H-8), 1.93 – 1.85 (m, 2H, H-7) ppm; ^{13}C NMR (100 MHz, DMSO-d_6): δ = 185.3 (C-4), 153.2 (C-2/6), 139.8 (ArC), 126.5 (ArC), 126.3 (C-3/5) 122.0 (ArC), 68.0 (C-1), 58.3 (CH_2-N), 50.0 (C-8), 38.6 (C-7) ppm; HRMS (EI) calcd. for $C_{16}H_{17}NO_2$ $[M]^+$ = 255.1259; Found: 255.1251.

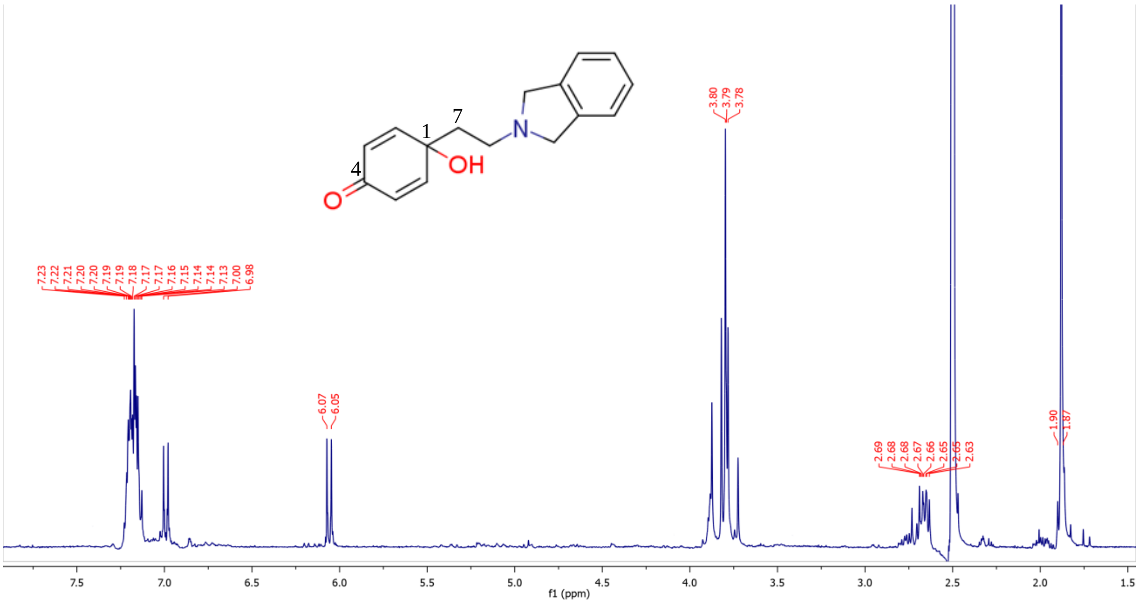

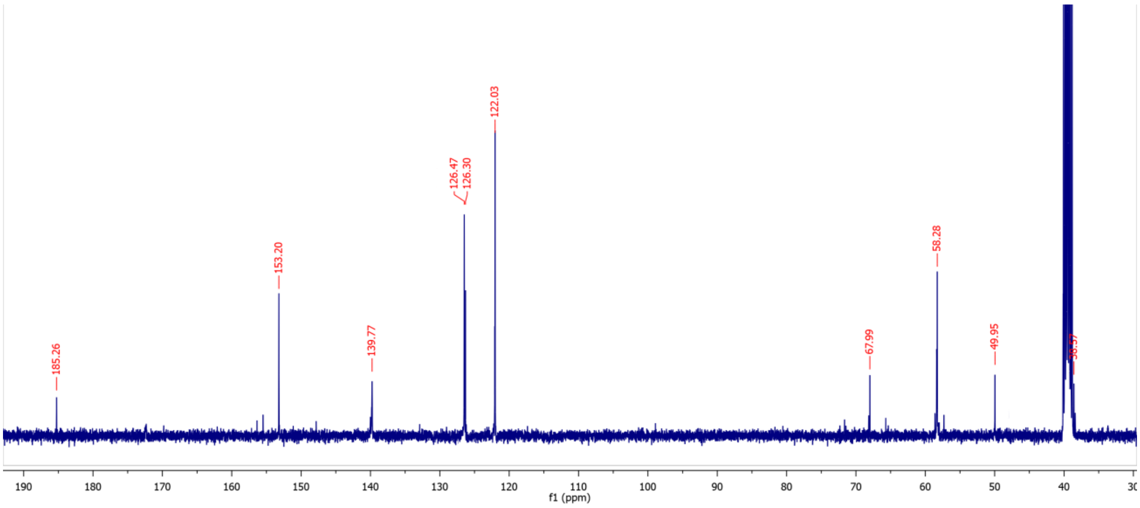

N-[2-(1-Hydroxy-4-oxocyclohexa-2,5-dien-1-yl)ethyl]morpholine **(14c)**

Brownish solid; Yield 28%; R_f = 0.37 (EtOAc:EtOH = 1:1); mp: 98-99°C; ^{1}H NMR (400 MHz, DMSO-d_6): δ = 6.95 (d, J = 10.0 Hz, 2H, H-2/6), 6.04 (d, J = 10.0 Hz, 2H, H-3/5), 3.56 - 3.46 (m, 4H, CH_2-O), 2.32 - 2.26 (m, 4H, CH_2-N), 2.25 – 2.20 (m, 2H, H-8), 1.79 (t, J = 7.6 Hz, 2H, H-7) ppm; ^{13}C NMR (100 MHz, DMSO-d_6): δ = 185.8 (C-4), 153.8 (C-2/6), 126.8 (C-3/5), 68.5 (C-1), 66.6 (CH_2-O), 53.7 (CH_2-N), 53.4 (C-8), 37.0 (C-7) ppm; HRMS (EI) calcd. for $C_{12}H_{17}NO_3$ $[M]^+$ = 223.1208; Found: 223.1201.

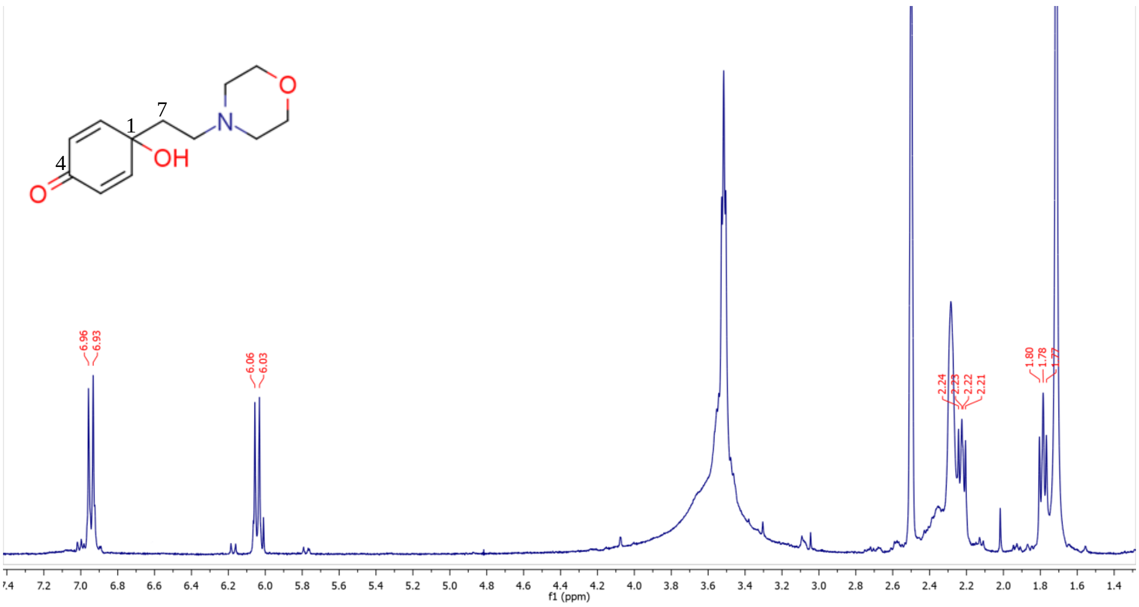

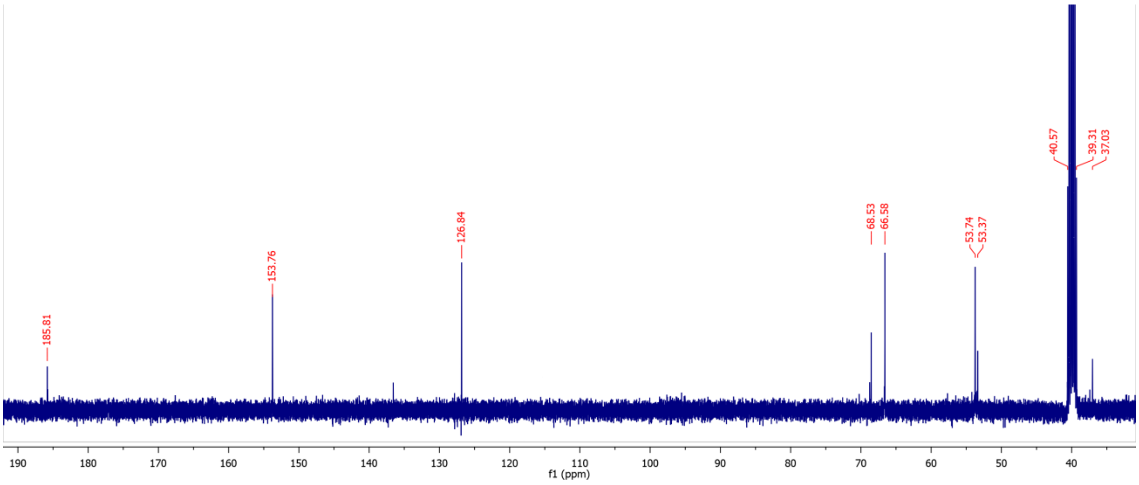

3-Hydroxy-N-[2-(1-hydroxy-4-oxocyclohexa-2,5-dien-1-yl)ethyl]octahydroisoindole-1-one **(15)**

Beige solid; Yield 65%; R_f = 0.14 (EtOAc); mp: 127-128°C; ^{1}H NMR (400 MHz, DMSO-d$_6$): δ = 6.97 – 6.91 (m, 2H, H-2/6), 6.08 (d, J = 11.0 Hz, 2H, H-3/5), 5.90 (d, J = 6.6 Hz, 1H, 9'-OH), 5.82 (s, 1H, 1-OH), 4.55 (d, J = 6.6 Hz, 1H, H-9'), 3.39 – 3.31 (m, 1H, H-8$_{(a)}$), 3.05 – 2.96 (m, 1H, H-8$_{(b)}$), 2.63 – 2.56 (m, 1H, H-3'), 2.06 – 1.98 (m, 1H, H-8'), 1.89 – 1.82 (m, 1H, H-7$_{(a)}$), 1.83 – 1.77 (m, 1H, H-4'$_{(a)}$), 1.80 – 1.72 (m, 1H, H-7$_{(b)}$), 1.72 – 1.66 (m, 1H, H-7'$_{(a)}$), 1.49 – 1.35 (m, 3H, H-4'$_{(b)}$/5'$_{(a)}$/6'$_{(a)}$), 1.19 – 1.06 (m, 1H, H-6'$_{(b)}$), 0.95 – 0.89 (m, 1H, H-5'$_{(b)}$), 0.91 – 0.83 (m, 1H, H-7'$_{(b)}$) ppm; ^{13}C NMR (100 MHz, DMSO-d$_6$): δ = 185.6 (C-4), 175.2 (C-2'), 153.2 (C-2/6), 127.3 (C-3/5), 85.6 (C-9'), 68.1 (C-1), 40.8 (C-8'), 38.5 (C-3'), 38.1 (C-7), 35.2 (C-8), 26.3 (C-7'), 23.3 (C-6'), 23.2 (C-4'), 23.1 (C-5') ppm; HRMS (EI) calcd. for $C_{16}H_{21}NO_4$ $[M]^+$ = 291.1471; Found: 291.1469.

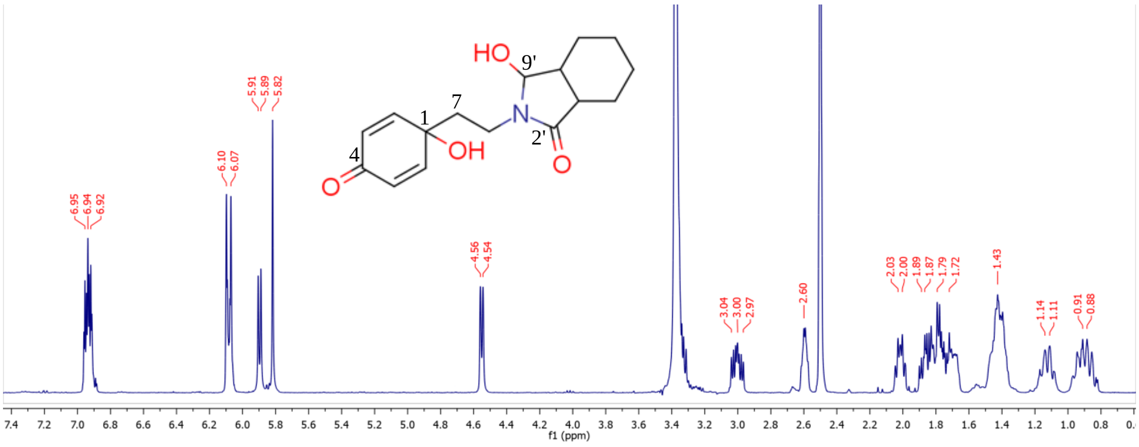

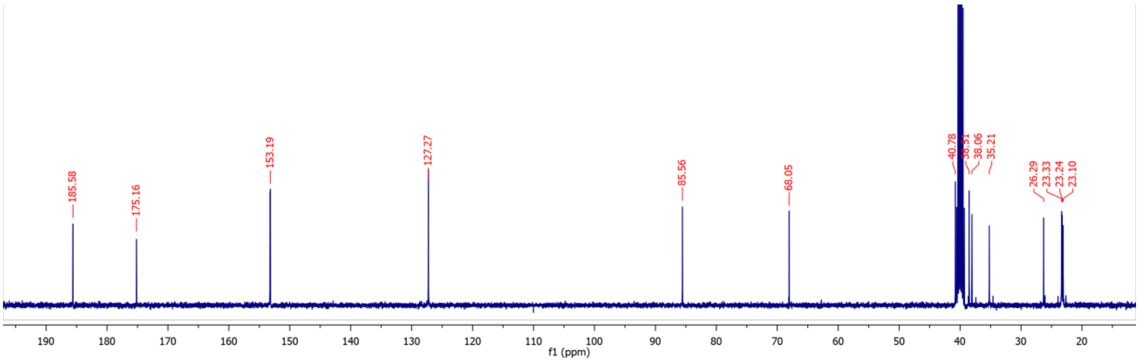

Supplement: Supplementary file 1 [file molecules-23-02902-s001.pdf]
